# Epigenetic Dysregulation of *KCNK9* Imprinting and Triple-Negative Breast Cancer

**DOI:** 10.3390/cancers13236031

**Published:** 2021-11-30

**Authors:** David A. Skaar, Eric C. Dietze, Jackelyn A. Alva-Ornelas, David Ann, Dustin E. Schones, Terry Hyslop, Christopher Sistrunk, Carola Zalles, Adrian Ambrose, Kendall Kennedy, Ombeni Idassi, Gustavo Miranda Carboni, Michael N. Gould, Randy L. Jirtle, Victoria L. Seewaldt

**Affiliations:** 1 Department of Biological Sciences, North Carolina State University, Raleigh, NC 27695, USA; daskaar@ncsu.edu; 2 Beckman Research Institute, Department of Population Sciences, City of Hope, Duarte, CA 91010, USA; edietze@coh.org (E.C.D.); jalvao@coh.org (J.A.A.-O.); dann@coh.org (D.A.); dsschones@coh.org (D.E.S.); csistrunk@coh.org (C.S.); aambrose@coh.org (A.A.); kkennedy@coh.org (K.K.); oidassi@coh.org (O.I.); 3 Department of Biostatistics, School of Medicine, Duke University, Durham, NC 27710, USA; terry.hyslop@duke.edu; 4 Department of Pathology, Mercy Hospital, Miami, FL 33133, USA; Czalles@baptisthealth.net; 5 Laboratory of Oncology, Department of Oncology, School of Medicine, University of Tennessee Health Science, Memphis, TN 38163, USA; gmirand1@uthsc.edu; 6 McArdle Laboratory for Cancer Research, University of Wisconsin-Madison, Madison, WI 53706, USA; gould@oncology.wisc.edu

**Keywords:** triple negative breast cancer, *KCNK9*, epigenetics, imprinting

## Abstract

**Simple Summary:**

Genomic imprinting is an inherited form of parent-of-origin specific epigenetic gene regulation that is dysregulated by poor prenatal nutrition and environmental toxins. Here, we showed that *KCNK9* is imprinted in breast tissue and identified the differentially methylated region (DMR) controlling its imprint status. Hypomethylation at the DMR, coupled with biallelic expression of *KCNK9*, occurred in 63% of triple-negative breast cancers (TNBC). The association between hypomethylation and TNBC status was highly significant in African-Americans (*p* = 0.006), but not in Caucasians (*p* = 0.70). The high frequency of *KCNK9* DMR hypomethylation in TNBC and non-cancerous breast tissue from high-risk women provides evidence that hypomethylation of the *KNCK9* DMR/TASK3 overexpression may provide a new target for prevention of TNBC.

**Abstract:**

Genomic imprinting is an inherited form of parent-of-origin specific epigenetic gene regulation that is dysregulated by poor prenatal nutrition and environmental toxins. *KCNK9* encodes for TASK3, a pH-regulated potassium channel membrane protein that is overexpressed in 40% of breast cancer. However, *KCNK9* gene amplification accounts for increased expression in <10% of these breast cancers. Here, we showed that *KCNK9* is imprinted in breast tissue and identified a differentially methylated region (DMR) controlling its imprint status. Hypomethylation at the DMR, coupled with biallelic expression of *KCNK9*, occurred in 63% of triple-negative breast cancers (TNBC). The association between hypomethylation and TNBC status was highly significant in African-Americans (*p* = 0.006), but not in Caucasians (*p* = 0.70). *KCNK9* hypomethylation was also found in non-cancerous tissue from 77% of women at high-risk of developing breast cancer. Functional studies demonstrated that the *KCNK9* gene product, TASK3, regulates mitochondrial membrane potential and apoptosis-sensitivity. In TNBC cells and non-cancerous mammary epithelial cells from high-risk women, hypomethylation of the *KCNK9* DMR predicts for increased TASK3 expression and mitochondrial membrane potential (*p* < 0.001). This is the first identification of the *KCNK9* DMR in mammary epithelial cells and demonstration that its hypomethylation in breast cancer is associated with increases in both mitochondrial membrane potential and apoptosis resistance. The high frequency of hypomethylation of the *KCNK9* DMR in TNBC and non-cancerous breast tissue from high-risk women provides evidence that hypomethylation of the *KNCK9* DMR/TASK3 overexpression may serve as a marker of risk and a target for prevention of TNBC, particularly in African American women.

## 1. Introduction

Epigenetic adaptations in response to in utero nutritional and environmental factors are hypothesized to play an important role in developmental plasticity and human disease susceptibility [1,2,3]. Diet-derived methyl donors and co-factors are necessary for the synthesis of S-adenosylmethionine (SAM), the methyl group donor for DNA methylation. Thus, environmental factors that alter early nutrition and/or SAM production can potentially influence adult disease risk by altering CpG methylation at critically important, epigenetically labile regulatory regions [4,5].

Genomic imprinting is an inherited form of parent-of-origin dependent epigenetic gene regulation that renders autosomal genes functionally haploid in a species, developmental stage, and tissue dependent manner [6,7]. There is evidence that epigenetic modifications in the genome link environmental exposures to adult disease susceptibility [5,8,9,10,11], including cancer [12,13,14,15]. Moreover, imprinting can be dysregulated not only in somatic cells, but also in germ cells, potentially affecting offspring never subject to the parental exposure [2,8]. Since imprinted genes are frequently clustered and coordinately regulated by differentially methylated regions (DMRs), changes in a single DMR can disrupt the expression of more than one imprinted gene [16,17]. Disease susceptibility due to epigenetic deregulation also has specific windows of vulnerability, including embryogenesis, puberty, pregnancy, and old age [5,18,19,20].

In a computational model, *KCNK9* was predicted to be regulated by imprinting [21]. *KCNK9* and its gene product, TASK3, is of interest for human health studies, as overexpression is strongly tied to cancer. *KCNK9* is maternally expressed in the human brain, as well as in the mouse brain [21,22]. The *KCNK9* gene encodes for the pH sensitive potassium channel protein, TASK3. TASK3 is present at the plasma membrane and regulates membrane depolarization in response to acidosis via inhibition of the background potassium-current [23,24]. Inactivation of the expressed maternal copy of *KCNK9* results in Birk-Barel syndrome [25]. Overexpression of TASK3 in cell lines promotes tumor formation and hypoxia-resistance [23,24,26]. Blocking the TASK3 channel protein, either chemically or by mutation, reduces cell proliferation and increases apoptosis, by unknown mechanisms [26,27]. However, TASK3 has been observed to also be localized in the mitochondria, in addition to the plasma membrane [28,29,30,31], and inhibition of TASK3 function has been shown to lead to mitochondrial dysfunction [29,30,31].

TASK3 is overexpressed in >40% of breast cancers, but genomic amplification of *KCNK9* only accounts for TASK overexpression in <10% of breast cancers [24]. *KCNK9* is known to be epigenetically regulated. Consequently, we hypothesized that overexpression of TASK3 protein (in the absence of *KCNK9* duplication) could be due to epigenetic dysregulation, specifically the loss of parental silencing methylation or imprinting.

In this study, we investigated the imprinting status of *KCNK9* in normal and malignant mammary epithelial cells. We demonstrated that it is monoallelically expressed and identified an associated DMR and regulatory DMR chromatin structure. In both cancer cells and mammary epithelial cells from high-risk women, we observed hypomethylation of the *KCNK9* DMR. Hypomethylation of the *KCNK9* DMR increased TASK3 protein expression and resulted in increased mitochondrial membrane potential and apoptosis-resistance. DMR hypomethylation, and an increase in mitochondrial membrane potential, was observed most frequently in triple negative breast cancer (TNBC).

## 2. Materials and Methods

DNA extraction: DNA was extracted from blood with the PAX gene blood DNA kit (Qiagen, Germantown, MD, USA), according to the manufacturer’s instructions. DNA was extracted from breast epithelial cells collected by Random Periareolar Fine Needle Aspiration (RPFNA). This DNA was isolated using standard procedures [32].

Methylation analysis: CpG methylation was measured by analysis of bisulfite converted DNA. See Appendix A Materials and Methods for details.

Nucleosome occupancy and methylome sequencing (NOMe-Seq): Nucleosome occupancy in conjunction with in vivo DNA methylation was measured as described previously [33]. In vitro methylated DNA was bisulfite converted using EpiTect Bisulfite Kits (Qiagen) according to manufacturer’s instructions and amplified using primers *KCNK9*-US0f and *KCNK9*-US4r, which avoid both CpG and GpC dinucleotides. Primer sequences are listed in Appendix A Materials and Methods. Amplicons were cloned and sequenced as described above.

Monoallelic expression analysis: Determination of mono- and biallelic gene expression was performed by cDNA sequencing. Tissues were genotyped at rs2615374 using Applied Biosystems components and equipment (Foster City, CA, USA), according to the manufacturer’s standard protocols as described in Appendix A Materials and Methods. Primer sequences are listed in Appendix A Materials and Methods.

Cloning human *KCNK9*: To clone *KCNK9,* 1 μg of human brain total RNA (Takara Bio USA, Mountain View, CA, USA) was transcribed into cDNA using the Superscript III First Strand Kit (Invitrogen, Waltham, MA, USA). *KCNK9* was amplified by PCR with BamHI ends from the cDNA and subcloned into pCR2.1 (Life Technologies, Carlsbad, CA, USA). *KCNK9* was then digested from pCR2.1 using BamHI and inserted into pLXSN (Takara Bio, Mountain View, CA, USA). Primer sequences are listed in Appendix A Materials and Methods.

LXSN transduction vector construction: A point mutation was generated in pLXSN *KCNK9* using the QuickChange II XL Site-Directed Mutagenesis kit (Stratagene, Santa Clara, CA, USA) to generate the dominant negative *KCNK9*-G95E. Retrovirus particles were generated by co-transfecting pLXSN *KCNK9* or *KCNK9*-G95E with VSV-G into the GP-293 packaging cell line (Takara Bio, Mountain View, CA, USA) as previously described [34]. Primer sequences for site directed mutagenesis are listed in Appendix A Materials and Methods.

FLAG-tagged *KCNK9* construction: *KCNK9* was cloned into pCr2.1 as described above. *KCNK9* was then digested from pCR2.1 using BamHI and inserted into pCMV 3TAG 1 (Agilent Technologies, Santa Clara, CA, USA).

Stable expression of TASK3 protein (*KCNK9* gene product): Using previously published methods [34], cell lines were transduced with empty LXSN, LXSN containing wildtype *KCNK9*, or LXSN containing *KCNK9*-G95E (dominant negative). Transduced cells were selected with G418 (Life Technologies): MDA231 using 2.0 µg/mL, DKAT using 0.50 µg/mL, SUM225 using 0.10 µg/mL, and HEK293 using 1.0 µg/mL. Expression of TASK3 was confirmed by SDS-PAGE and western analysis.

Transient expression of TASK3 protein (*KCNK9* gene product): MCF10A cells were transiently transfected with FLAG-tagged empty plasmid or FLAG-Tagged *KCNK9* plasmid for 24 h using X-treme GENE HP DNA (Roche, Indianapolis, IN, USA) according to the manufacturer’s instructions. Expression of the *KCNK9* protein product, TASK3, in the cells was confirmed by immunofluorescence. Experiments were performed in triplicate.

Cell Lines: Cell lines were grown at 37 °C and 5% CO_2_ in a humidified chamber. See Appendix A Materials and Methods for details.

SDS-PAGE and western analysis: SDS-PAGE was performed as previously published [35]. Primary antibodies include 1/1000 *KCNK9*, ARP35260_P050 (Aviva Systems Biology, San Diego, CA, USA); 1/1000 goat GAPDH, sc-20357 (Santa Cruz Biotechnology, Santa Cruz, CA, USA); 1/500 VDAC1, sc-390996 (Santa Cruz Biotechnology). Secondary antibodies were sc-2313 (anti-rabbit) and sc-2020 (anti-goat) (Santa Cruz Biotechnology), used at a dilution of 1/20,000.

5-Aza-2′-deoxycytidine treatment: MCF10A (data shown) and HMEC15 (data not shown) cells were treated with 1.0 or 2.5 µM of 5-aza-2′-deoxycytidine (Sigma Aldrich, St. Louis, MO, USA) or vehicle control for up to 72 h, with fresh medium added after 24 h. Cells were harvested every 24 h for determining DNA methylation and ΔΨ_M_. Expression of TASK3 was detected via western blot. Experiments were performed in triplicate. Total RNA was also extracted for *KCNK9* mRNA quantitation and cell growth was measured using an MTT assay. See Appendix A Materials and Methods for details.

Apoptosis and measurement of ΔΨ_M_: Apoptosis was assessed by measuring intracellular caspase-3 activity. ΔΨ_M_ in cell lines was detected using JC-1 (Life Technologies). ΔΨm was also determined in aspirated RPFNA cells from patients. The cells were collected as described below. An aspirate was removed from the subject and was analyzed immediately after collection. See Appendix A Materials and Methods for details.

Mitochondrial localization of TASK3: Transient expression of FLAG-tagged TASK3 in MCF10A cells was detected by immunofluorescence using anti-FLAG antibody (Sigma Aldrich) according to the manufacturer’s instructions. The expression of FLAG-tagged TASK3 in the mitochondria was assessed by SDS-PAGE and western analysis of purified mitochondria (see below).

Isolation of mitochondria: High purity mitochondria were isolated using a Qproteome mitochondrial isolation kit, according to the manufacturer’s instructions (Qiagen).

Human subjects and tissue collection: Tissue collection protocols were approved by the Human Subjects Committee and the Institutional Review Board at Duke University. High-risk women, or women with cancer, were sequentially recruited by a research coordinator in order of presentation. All women provided informed consent to participate in this study. Demographic data were collected by patient interview. Definition of high-risk is described in detail in Appendix A Materials and Methods. All tissues underwent pathology review by a pathologist who was blinded to the results of this study.

RPFNA of breast epithelial cells and Masood Cytology Score: RPFNA was performed by our published methods [32,36]. Masood Cytology Score and epithelial cell count for duplicate RPFNA samples were assigned by a sample blinded, single dedicated cytopathologist as previously reported [36].

Statistical analysis: Assessment of the association of breast cancer subtype with methylation status was performed using Fisher’s exact test. Stratified contingency tables and Fisher’s exact tests were used to assess the association of breast cancer subtype with methylation status by race. Association was estimated for total hypomethylation, partial hypomethylation, normal, and hypermethylated status versus ER+, HER2+, and TNBC (and also categorized as hypomethylated total versus not, and TNBC yes versus not). The association of Masood Cytology Index Score and R/G ratio versus methylation status in high-risk women was completed using linear models with robust standard errors to control for the correlation of multiple measures per patient. Least squares means are reported with paired comparisons across methylation status levels as well as 95% confidence intervals of mean differences. Adjustment of confidence intervals and p-values for the two comparisons to normal methylation status was based on a Bonferroni adjustment [37]. Statistical analysis was completed in SAS v9.4 (SAS Institute, Cary, NC, USA) and in Stata v12 (StataCorp LP, College Station, TX, USA).

## 3. Results

### 3.1. KCNK9 Is Imprinted in Mammary Epithelial Cells

#### 3.1.1. *KCNK9* Exhibits Monoallelic Expression in Breast Tissue

**Figure 1:**
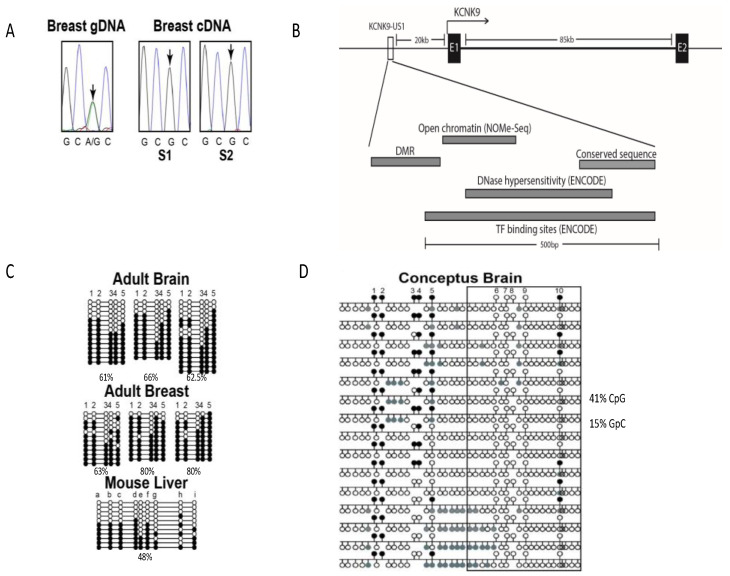
*KCNK9* is imprinted in human breast tissue. (**A**) Sequencing of *KCNK9* coding single nucleotide polymorphism (SNP) rs2615374 from breast tissue. Individuals S1 and S2 were heterozygous for this SNP (A/G), but only one allele (G) was expressed. The position of rs2615374 used to determine allelic expression is indicated by the arrows. (**B**) Exon 1 (E1), exon 2 (E2). Bars indicate the regions of differential methylation (DMR), open chromatin (NOMe-Seq) determined in this study, regions of cross species conserved sequence (human GRCh37/hg19 compared to mouse GRCm38/mm 10), DNase hypersensitivity (ENCODE), and transcription factor binding (ENCODE). Transcription factors binding in this region include NRSF, GATA3, and AP-2. (**C**) Sequencing of cloned methyl-PCR products from the upstream DMR (*KCNK9*-US1 DMR) in adult human brain and breast, and the orthologous *KCNK9* upstream sequence in mouse liver, open circle, unmethylated cytosine; black filled circle, methylated cytosine in the differentially methylated region (DMR). (**D**) NOMe-Seq of cloned methyl-PCR products from *KCNK9*-US1 DMR in conceptus brain, showing an inverse relation between CpG methylation and open chromatin structure. Upper circles—CpG sites (in vivo methylation: open circle, unmethylated cytosine; black filled circle, methylated cytosine). Lower circles—GpC sites (in vitro methylation, chromatin structure dependent: open circle, unmethylated cytosine, closed chromatin; gray filled circle, methylated cytosine, open chromatin). Boxed region—area of transcription factor binding from ENCODE data (Figure 1B).

Monoallelic expression of *KCNK9* in normal mammary epithelial cells was tested using the single nucleotide polymorphism, rs2615374; this was the same polymorphism used to identify monoallelic expression in the brain [21]. Using matched DNA/RNA samples extracted from adult breast tissue, monoallelic expression was observed in two samples heterozygous for rs2615374 (Figure 1A).

#### 3.1.2. Identification of the *KCNK9* Imprint DMR

Analysis of the canonical CpG island covering the *KCNK9* promoter region, transcription factor binding sites, transcription start site, and first exon identified no allele-specific methylation (Table A1). Expanding the search by a bioinformatic approach, using ENCODE data and cross-species sequence conservation, we identified a *KCNK9* upstream region (*KCNK9*-US1) that had characteristics of an imprint regulatory sequence (Figure 1B). Approximately 20 kb upstream of the *KCNK9* start site is a 500 bp region (chr8:140,732,400-140,732,870 GRCh37/hg19) containing areas of strong cross-species conservation, DNase hypersensitivity, and binding of multiple transcription factors [38,39,40]. Immediately adjacent to the conserved sequence is a cluster of CpG sites, but the sequence containing these dinucleotides has no cross-species conservation [39]. Quantitative methylation analysis by Sequenom MassArray identified intermediate methylation (20–70%) for five consecutive CpG sites within this adjacent region in brain, breast, liver, and testis from eight different adults (Table 1). These differentially methylated CpG sites are not within the ENCODE defined DNase hypersensitive region, nor the majority of the transcription factor binding regions [38]. Sequencing of PCR clones shows continuity of methylated or unmethylated cytosines for this region in DNA from the adult brain, with the adult breast showing a higher average methylation level (Figure 1C).

While the putative *KCNK9*-US1 DMR has no sequence similarity to mouse, human and mouse have comparable numbers of CpG sites and spacing of these sites [40]. Humans have 10 CpG sites in 385 bp with 53% GC content, for an observed:expected CpG ratio of 0.37. Mice have 12 CpG sites in 291 bp with a 51% GC content, for an observed:expected CpG ratio of 0.64. Clone sequencing of this region in mouse liver identified a DMR comparable to that observed in humans, with five CpG sites showing strong contiguous methylation (sites a–d, and f) (Figure 1C).

#### 3.1.3. Methylation of the *KCNK9* DMR Regulates Chromatin Structure

The ENCODE data suggested open regulatory chromatin near the *KCNK9*-US1 DMR, based on observed DNase hypersensitivity and transcription factor binding (Figure 1B). NOMe-seq was used to determine the relationship between CpG methylation and chromatin structure at the DNase hypersensitive and transcription factor binding sites [33,41]. Chromatin structure dependent in vitro methylation by bacterial GpC methyltransferase identified 10 consecutive GpC dinucleotides over ~130 bp as consistently methylated or demethylated in the conceptus brain (Figure 1D). Clone sequencing showed an inverse relationship between this region of differential open/closed chromatin and methylation at DMR CpG sites 3 and 4 (Figure 1D). This strong correlation was observed in conceptus brain tissue, where *KCNK9* mRNA expression is highest, with a lesser amount of open chromatin seen in conceptus kidney and liver (data not shown).

#### 3.1.4. Methylation Status of the *KCNK9* DMR Does Not Correlate with Age

**Figure 2:**
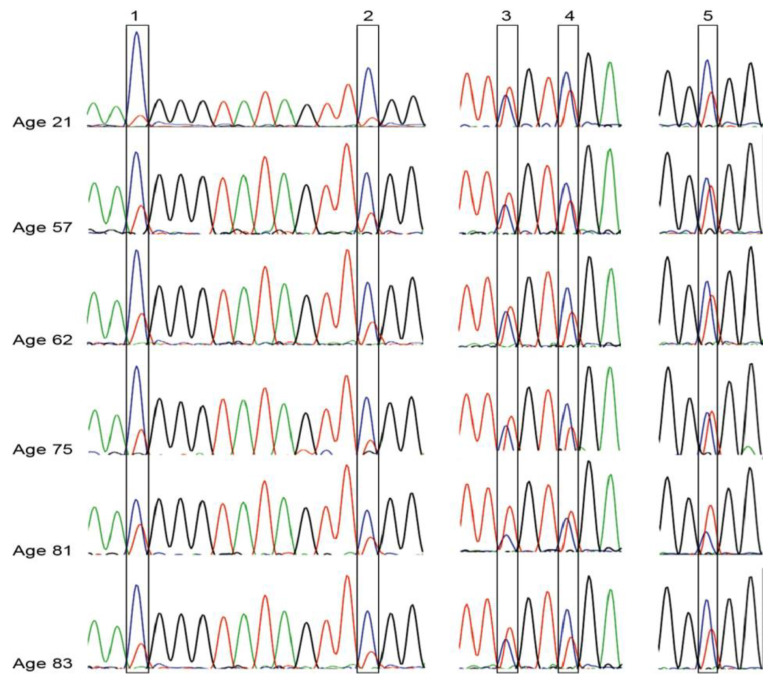
Representative DNA methylation of *KCNK9*-US1 DMR in non-cancerous breast tissue. Commercially obtained breast genomic DNA (Biochain, Newark, CA, USA) was obtained for analysis. The results are shown from six women, ranging in age from 21 to 83 years old.

We used bisulfite sequencing to determine (1) the consistency of *KCNK9*-US1 DMR methylation in individuals without breast cancer and (2) whether *KCNK9*-US1 DMR hypomethylation increased with increasing age. Genomic DNA from six women with no breast cancer history, ages 21 to 83 years, showed consistent methylation for each of the five CpG sites for five individuals, while one 81-year-old individual had marginal hypomethylation at sites 3 to 5 (Figure 2).

### 3.2. Hypomethylaiton of the KCNK9 DMR Is Observed in Invasive Breast Cancer; Hypomethylation of the KCNK9 DMR Increases TASK3 Protein Expression

#### 3.2.1. *KCNK9* DMR Analysis and Invasive Breast Cancer

**Figure 3:**
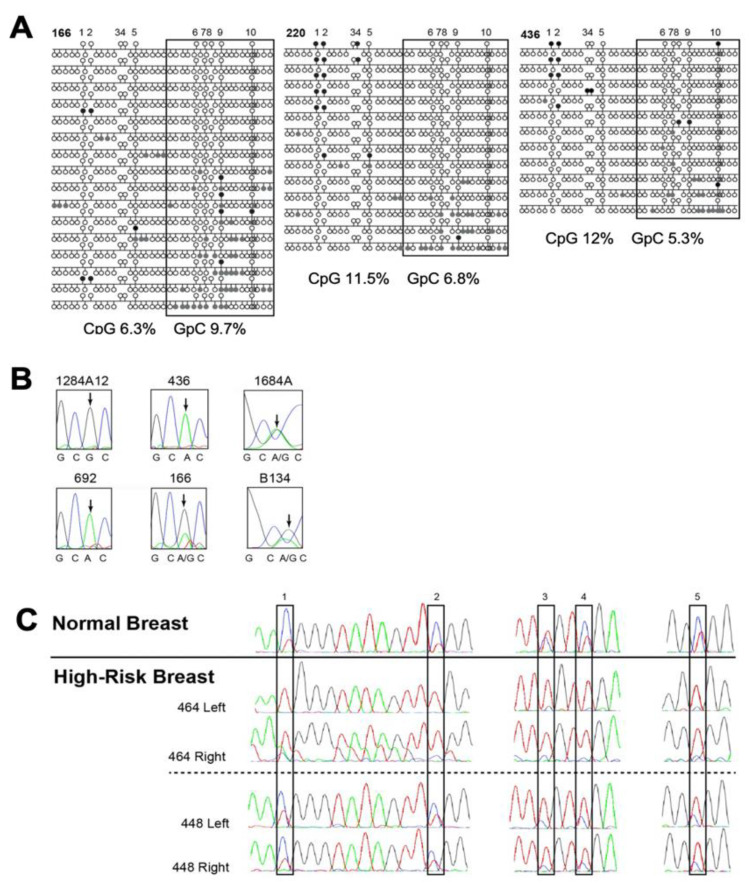
DNA methylation of *KCNK9*-US1 DMR in breast cancer and microdissected mammary epithelial cells aspirates from women at high risk for breast cancer. (**A**) NOMe-Seq of the cloned *KCNK9*-US1 DMR from breast cancer cores (166, 220, and 436). Upper circles—CpG sites (in vivo methylation: open circle, unmethylated cytosine; black filled circle, methylated cytosine). Lower circles—GpC sites (in vitro methylation, chromatin structure dependent: open circle, unmethylated cytosine, closed chromatin; gray filled circle, methylated cytosine, open chromatin). Boxed region—area of transcription factor binding from ENCODE data. (**B**) Sequences of coding SNP rs2615374 (A/G) in cDNA from tumor cores (1284A12, 436, 1684A, 692, and 166) and normal epithelial cells (B134) heterozygous for the SNP. The position of rs2615374 used to determine allelic expression is indicated by the arrows. (**C**) Hypomethylation at CpG sites 1 to 5 (boxes) in the *KCNK9*-US1 DMR in DNA from non-cancerous mammary epithelial cell aspirates from women who are at high risk for developing breast cancer. Differentially methylated region (DMR).

Analysis of *KCNK9* DMR methylation in invasive cancer biopsy specimens indicated that the most significant differences in methylation between brain and invasive cancer biopsies were for sites 3 to 5. Sites 3 to 5 were the most highly correlated to chromatin structure by NOMe-seq. NOMe-seq of three invasive cancers with DMR hypomethylation indicated open chromatin was linked to DMR hypomethylation (Figure 3A). The open chromatin position in tumor cores (GRCh37/hg19 Chr8:~140732560–140732690) is shifted from that observed in the conceptus brain (Chr8:~140732450–140732630), but is within the ENCODE defined region containing transcription factor binding sites (Figure 1B). Expression analysis by cDNA sequencing of rs2615374 in heterozygous core and epithelial samples showed expression of both alleles, i.e., LOI, in 36% (4/11) of the breast tumors informative for rs2615374 (Figure 3B). As discussed below, methylation was variable in non-cancerous breast tissue from high-risk women, with some individuals showing hypomethylation at sites 3–5, and others showing hypomethylation at sites 1–5 (for a representative analysis of two women, see Figure 3C).

#### 3.2.2. Chemical Demethylation of the *KCNK9* DMR Increases Expression of the *KCNK9* Gene Product, TASK3

**Figure 4:**
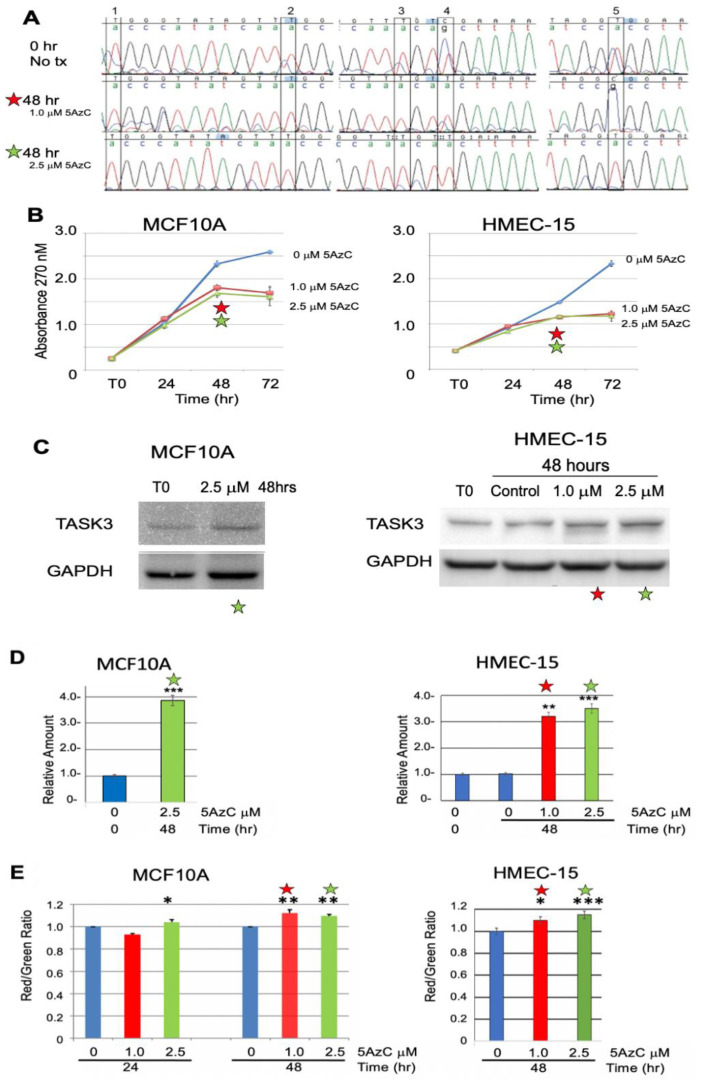
TASK3 expression and mitochondrial membrane potential as a function of *KCNK9*-US1 DMR demethylation in MCF10A cells and HMEC-15 normal cell strain. The optimal treatment conditions are 48 h for either 1.0 (red star) or 2.5 µM (green star) 5-aza-2′-deoxycytidine (5AzC) (**A**) Effect of 1.0 and 2.5 µM 5AzC on the DNA methylation of CpG sites 1–5 (boxes) in the *KCNK9*-US1 DMR in MCF10A. (**B**) Cell proliferation (viability) was tested using the MTT assay at 0–72 h; MCF10A cells and HMEC-15 normal cell strain were treated with 0 (blue line), 1.0 (red line), and 2.5 µM (green line) 5-AzC. Experiments were performed in triplicate. (**C**) Expression of TASK3 protein analyzed by western blotting at 0 and 48 h in MCF10A after treatment with 2.5 µM 5AzC and HMEC-15 normal cell strain after treatment with 1.0 and 2.5 µM 5AzC. GAPDH is used as the loading control. (**D**) Relative TASK3 expression in MCF10A after treatment with 2.5 µM 5AzC for 0 and 48 h and HMEC-15 normal breast cell strain after treatment with 1.0 and 2.5 µM 5AzC for 0 and 48 h. TASK3 expression was normalized to 0 h treatment (Control). Expression is for the western blots performed in triplicate for panel Figure 4C. Significant levels relative to 0 h control: *** *p* < 0.001. (**E**) JC-1 R/G ratio in MCF10A cells treated with 0, 1.0, and 2.5 µM 5AzC for 0, 24, and 48 h normalized to 0 h treatment (Control). JC-1 R/G ratio in HMEC-15 normal cell strain was treated with 0, 1.0, and 2.5 µM 5AzC for 0 and 48 h normalized to 0 h treatment (Control). Significant levels relative to 0 h control: * *p* < 0.05, ** *p* < 0.01, *** *p* < 0.001. Experiments were performed in triplicate. The uncropped blots and molecular weight markers are shown in Supplementary Materials.

Treatment of TASK3-low expressing MCF10A cells for 48 hrs with 1.0 and 2.5 µM of the demethylating agent 5-aza-2′-deoxycytidine (5AzC) resulted in *KCNK9*-US1 DMR demethylation at both concentrations (Figure 4A). There was no significant difference in cell viability at 24 h in MCF10A cells or normal mammary epithelial cell strain HMEC-15 treated with either 1.0 or 2.5 µM 5AzC (Figure 4B). At 48 h there was a decrease in cell proliferation (versus untreated control cells) after treatment of MCF10A cells and HMEC15 with either 1.0 or 2.5 µM 5AzC (Figure 4B). After treatment of MCF10A cells with 2.5 µM 5AzC for 48 h, there was a 3.2-fold increase in TASK3 protein; after treatment of HMEC-15 with 0, 1.0, or 2.5 µM 5AzC there was, respectively, a 3.2 and 3.4-fold increase in TASK3 protein relative to T0 control (Figure 4C,D; 4D is a quantitation of results in 4C, including triplicate blots not shown in this figure). There was a statistically significant increase in mitochondrial membrane potential as measured by JC1 R/G ratio after 24 and 48 h treatment of MCF10A cells with 1.0 and 2.5 µM 5AzC; similar results were observed for HMEC-15 strain treated with 1.0 and 2.5 µM 5AzC for 48 h (Figure 4E).

### 3.3. Exogenous Expression of TASK3 Increases Mitochondrial Expression of TASK3, ΔΨ_M_, and Promotes Resistance to Staurosporine-Induced Apopotosis

**Figure 5:**
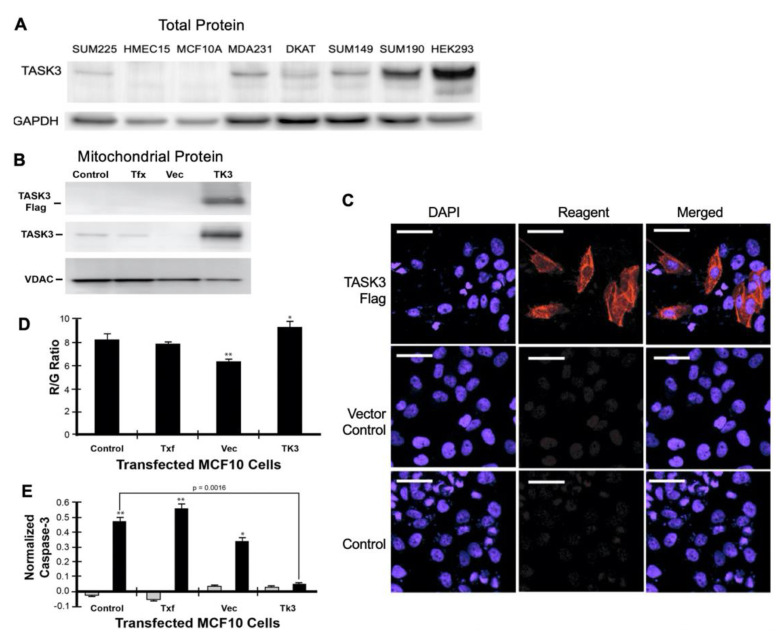
TASK3 expression and localization in breast cancer cell lines and mammary epithelial cell lines and cell strains. (**A**) Endogenous total TASK3 protein expression in human breast cell lines. TASK3 exhibited relatively increased expression in MDA231, DKAT, SUM149, SUM190, and HEK293 cells, but had lower expression in SUM225, HMEC15, and MCF10A cells. GAPDH serves as a loading control (*n* = 3 experiments). (**B**) Transient overexpression of TASK3-FLAG tag in the breast cancer cell line MCF10A results in expression of the exogenous construct in the mitochondria, as shown by western blotting. VDAC was used as a loading control for the mitochondrial fraction. Control untreated cells (Control), cells treated with transfection reagent alone (Txf), cells transfected with empty FLAG tag plasmid (Vec), and cells transfected with TASK3-FLAG tag plasmid. (**C**) Expression of the TASK3-FLAG construct by immunofluorescence. Control untreated cells (Control), cells transfected with empty FLAG tag plasmid (Vector Control), and cells transfected with TASK3-FLAG tag plasmid (TASK3-FLAG). TASK expression was detected by immunofluorescence using anti-FLAG tag antibody (Sigma-Aldrich, St. Louis, MO, USA). TASK3 transfected cells (TASK3-FLAG) were compared with those transfected with an empty vector control. (**D**) JC-1 Red/Green Ratio (R/G Ratio) in untreated MCF10A cells (Control), cells treated with transfection reagent alone (Txf), cells transfected with empty FLAG tag plasmid (Vec), and cells transfected with TASK3-FLAG tag plasmid (TK3). The R/G Ratio is a measure of mitochondrial membrane potential (ΔΨm), (*n* = 3 experiments). Significant levels relative to control: * *p* < 0.05, ** *p* < 0.01. (**E**) Caspase-3 activity, a measure of apoptosis initiation, normalized to DNA content in untreated MCF10A cells (Control); cells treated with transfection reagent alone (Txf); cells transfected with empty FLAG tag plasmid (Vec); and cells transfected with TASK3-FLAG tag plasmid (TK3). Cells were exposed for 4 h to either 0.0 µg/mL (grey bar) or 0.50 µg/mL (black bar) of staurosporine. Significant levels relative to cells not exposed to staurosporine: * *p* < 0.05, ** *p* < 0.01. The uncropped blots and molecular weight markers are shown in Supplementary Materials.

TASK3 protein expression was compared in breast cancer cell lines. The SUM225 cell line, HMEC-15 strain, and non-cancerous immortalized MCF10A cell line had lower expression of TASK3 protein relative to the breast cancer cell lines MDA231, DKAT, SUM149, SUM190, and HEK293 (Figure 5A).

Transient transfection using a FLAG-tagged construct was used to overexpress TASK3 (Figure 5B) in the TASK3-low expressing MCF10A cells (Figure 5A). The overexpressed FLAG-tagged TASK3 localized to the mitochondria as demonstrated by western analysis (Figure 5B). Immunohistochemistry demonstration of the FLAG-tagged construct is shown in Figure 5C.

**Figure 6:**
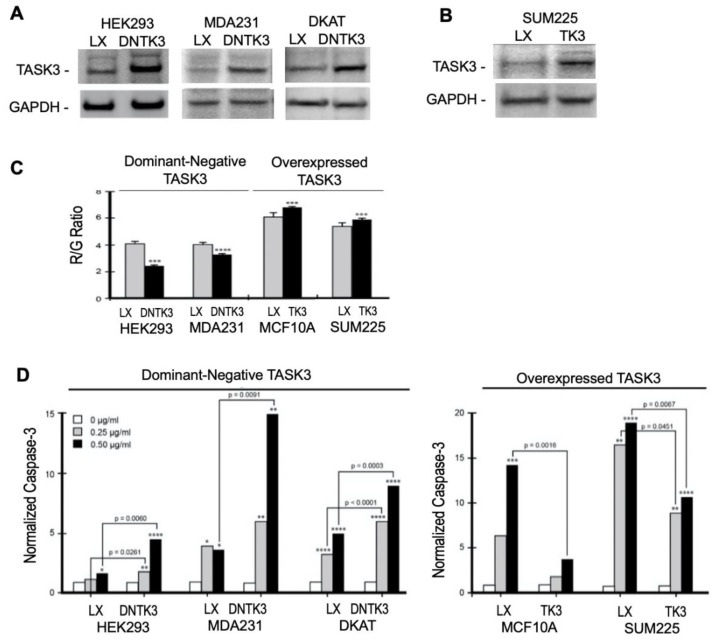
TASK3 expression regulates mitochondrial membrane potential and sensitivity to staurosporine-induced apoptosis. (**A**) Expression of the dominant negative TASK3-G95E construct in TASK3-expressing cell lines HEK293, MDA231, and DKAT. TASK3 was detected by SDS-PAGE and western analysis in LSXN vector controls (LX) and cells transduced with the dominant negative construct (DNTK3). GAPDH serves as a loading control (*n* = 3 experiments). (**B**) Overexpression of TASK3 in SUM225 cells. TASK3 was detected in vector control cells (LX) and cells transduced with TASK3 (TK3) by SDS-PAGE and western analysis. GAPDH serves as a loading control (*n* = 3 experiments). (**C**) JC-1 R/G ratio in LX transfected breast cancer cell lines, and cell lines transfected with either the dominant negative TASK3-G95E (DNTK3) or the TASK3 (TK3) construct (*n* = 4 experiments). Significant levels relative to LX control: *** *p* < 0.001, **** *p* < 0.0001. (**D**) TASK3 expression and apoptosis sensitivity in breast cancer cell lines. Cells were exposed for 4 h to 0.0 µg/mL (white bar), 0.25 µg/mL (grey bar), and 0.50 µg/mL (black bar) of staurosporine. Normalized caspase-3 activity, as a measure of apoptosis, in HEK293, MDA231, and DKAT cell lines expressing the dominant-negative TASK3-G95E construct (DNTK3). Normalized caspase-3 activity was also determined in MCF10A and SUM225 cells overexpressing TASK3 (TK3). Caspase-3 expression in these cell lines was also determined in their respective LXSN vector controls (LX) (*n* = 3 experiments). Significant levels relative to exposed cells: * *p* < 0.05, ** *p* < 0.01, *** *p* < 0.001, **** *p* < 0.0001. The uncropped blots and molecular weight markers are shown in Supplementary Materials.

We tested whether TASK3 expression had an impact on mitochondrial membrane potential (ΔΨ_M_) and apoptosis-sensitivity using a knock-in/knock-out approach (Figure 5D,E and Figure 6). Overexpression of wild-type TASK3 significantly increased ΔΨ_M_ relative to controls in the TASK3-low expressing cells, MCF10A (*p* = 0.0003), and SUM225 (*p* = 0.0037) (Figure 6C). These observations are also consistent with ΔΨ_M_ after treatment of the TASK3-low expressing MCF10A cells with 5-aza-2′-deoxycytidine (Figure 4C).

The LXSN retroviral vector was used to (1) stably express the dominant–negative TASK3-G95E construct in the TASK3-expressing cells, HEK293, MDA231, and DKAT (Figure 6A) and to (2) overexpress wild-type TASK3 in TASK3-low expressing cell line SUM225 (Figure 6B). To ensure that our results were neither cell line- nor construct-dependent, we also tested the FLAG-tagged TASK3 construct expressed in MCF10A cells above in Figure 5. The voltage sensitive dye JC-1 was used to test ΔΨ_M_ relative to TASK3 expression and function. In TASK3-G95E-expressing cells, ΔΨ_M_ was significantly decreased relative to controls in HEK293 (*p* = 0.0076) and MDA231 (*p* < 0.0001) (Figure 6C). These results show that TASK3 expression regulates ΔΨ_M_.

Apoptosis sensitivity was tested by treatment with staurosporine. Staurosporine is known to promote apoptosis and is used as an apoptosis-control. Expression of TASK3-G95E significantly increased apoptosis-sensitivity of HEK293 (*p* = 0.006), MDA231 (*p* = 0.0091), and DKAT (*p* = 0.0003) cells (Figure 6D), while overexpression of TASK3 in MCF10A (*p* = 0.0016) and SUM225 (*p* = 0.0067) cells resulted in apoptosis-resistance relative to controls, as measured by caspase-3 activity, an indicator of the early stages of apoptosis (Figure 5E and Figure 6D). These results are consistent with a study by Nagy et al. who showed that, in melanoma, TASK3 regulated apoptosis and mitochondrial function [30].

### 3.4. Hypomethylation of the KCNK9 DMR Is Most Frequently Observed in African-American Women with TNBC versus Caucasians with TNBC

#### 3.4.1. High-Risk Sample Set

In Table 2 and Table 3, we performed detailed methylation sequencing in biopsies obtained in a highly annotated group of high-risk women. Table 3 includes subjects from Table 2; subjects were only counted once. All women were followed by Dr. Victoria Seewaldt while at Duke University and were part of her high-risk cohort. High-risk was defined as (1) greater than 20% lifetime risk for breast cancer or (2) the presence of a deleterious germline mutation (e.g., mutation in BRCA1, BRCA2, etc.).

#### 3.4.2. Hypomethylation of the *KCNK9* DMR Was Most Frequently Observed TNBC

We tested for hypomethylation at the *KCNK9*-US1 DMR in 53 primary human breast cancers (Table 2). TNBCs had the highest frequency of DMR hypomethylation. There was not a significant association between breast cancer subtype and DMR methylation status, with hypomethylation present in 27% of HER2+ cancer, 39% of ER+ cancer, and 63% of TNBC (*p* = 0.11), with borderline significant association in TNBC, *p* = 0.06.

#### 3.4.3. *KCNK9* DMR Hypomethylation Is Observed in Our Dataset More Frequently in High-Risk African-American Women with TNBC

Hypomethylation at the *KCNK9* DMR in TNBC tumors was (1) much more frequently observed in African-Americans with TNBC than in European-American with TNBC and 2) was highly significant in African-Americans, but not in European-Americans (Table 2).

In African-Americans, hypomethylation at the *KCNK9* DMR was seen in 20%, 40%, and 92% of ER+, Her2+, and TNBC, respectively (*p* = 0.006). In European-Americans, hypomethylation at the *KCNK9* DMR was seen in 46%, 17%, and 27% of ER+, Her2+, and TNBC, respectively (*p* = 0.42) (Table 2).

We also attempted to test for hypomethylation of *KCNK9* DMR imprinting in a methylation dataset from cBioportal.org, generated from Illumina 450 chip analysis of CpG sites (1500 bp upstream of the transition start site of *KCNK9*). Unfortunately, the *KCNK9* imprint DMR lies significantly upstream of this region, and we were not able to test whether hypomethylation of *KCNK9* DMR methylation occurred at a higher frequency in African-American women in this expanded dataset.

### 3.5. Hypomethylation of the KCNK9 DMR Is Observed in Both TNBC and Non-Cancerous Breast Tissue, but Not in WBCs

#### Hypomethylation of the *KCNK9* DMR Methylation Is Observed in Non-Cancerous Tissue

To determine the utility of *KCNK9*-US1 DMR methylation as an early breast cancer diagnostic, we tested whether hypomethylation was detectable prior to cancer diagnosis in high-risk women in morphologically normal breast tissue, or WBCs in women with invasive breast cancer. Hypomethylation of the *KCNK9*-US1 DMR methylation was assessed in matched WBCs and non-cancerous breast tissue from 15 high-risk women and 18 women with invasive cancer (Table 3). Of the 14 women for whom RPFNA DNA was available for both breasts, 14% (2/14) of the women had *KCNK9*-US1 DMR partial or full hypomethylation in both samples; 57% (8/14) showed partial or full DMR hypomethylation in one or both breast samples (Table 3). Methylation was variable, with some individuals showing hypomethylation at sites 3–5, and others showing hypomethylation at sites 1–5 (Figure 3C). The observed hypomethylation of the *KCNK9*-US1 DMR in breast tissue, but not in WBC, is consistent with epigenetic alterations at the *KCNK9* locus occurring after embryonic implantation and the establishment of three fetal germ layers (i.e., mesoderm, ectoderm, and endoderm).

### 3.6. KCNK9 DMR Hypomethylation Is Not Associated with the Degree of Cytologic Abnormality, but Is Associated with Increased Mitochondrial Membrane Potential

The relationships between *KCNK9*-US1 DMR methylation and ΔΨ_M_ (measured by JC-1 R/G ratio) or Masood Score (a measure of cytologic abnormality) [36] were investigated in aspirated human mammary epithelial cells. Quantitative DNA methylation analysis was performed and *KCNK9*-US1 DMR methylation was classified as methylation (75–125% methylation), partial hypomethylation (20–74%), or hypomethylation (0–20% methylation). Mammary epithelial cell cytology was classified by the Masood Score [36].

**Figure 7:**
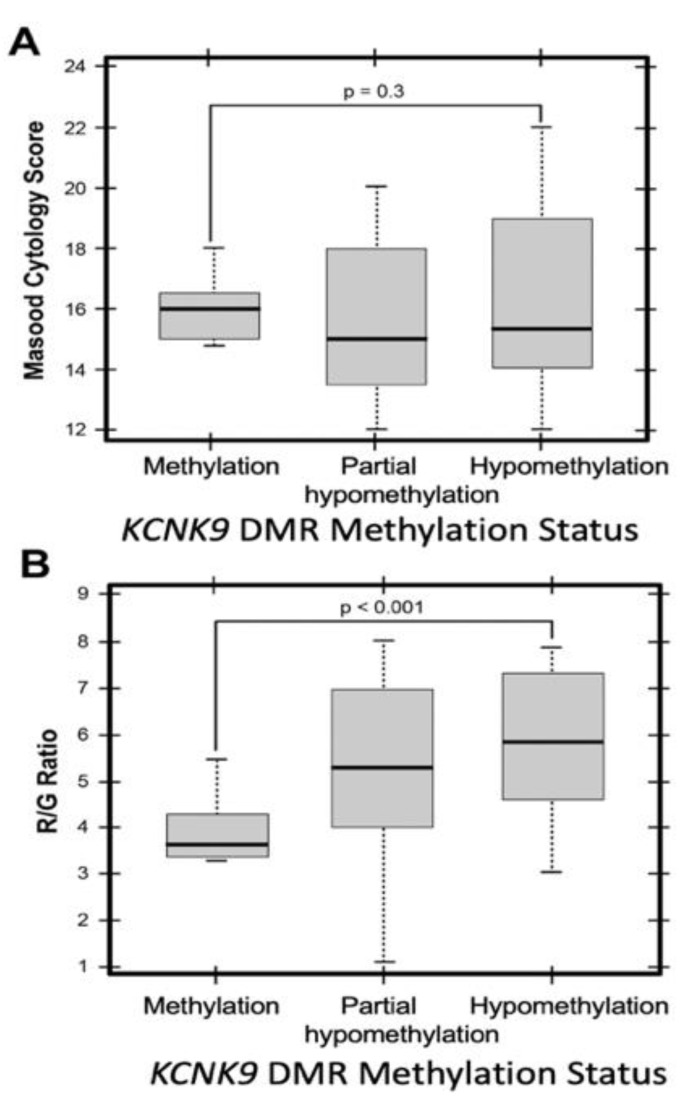
*KCNK9*-US1 DMR methylation in breast epithelial cells aspirated from women at high risk for developing breast cancer. (**A**) Box plot of Masood Cytology Index Score versus *KCNK9*-US1 DMR methylation status. (**B**) Box plot of R/G ratio versus *KCNK9*-US1 DMR methylation status: methylation, partial hypomethylation, hypomethylation.

When samples were grouped by methylation status, *KCNK9*-US1 DMR methylation was not significantly associated with the Masood Score (*p* = 0.3) (Figure 7A). In contrast, ΔΨ_M_ was significantly higher in mammary epithelial cells with a hypomethylated *KCNK9*-US1 DMR than in those where it was methylated (*p* < 0.001) (Figure 7B). Thus, hypomethylation of the *KCNK9*-US1 DMR predicts ΔΨ_M_ independent of the degree of cytologic abnormality.

## 4. Discussion

Loss of normal imprinting (1) occurs due to poor prenatal nutrition or exposure to heavy metals (e.g., cadmium, lead, arsenic) and (2) is linked to obesity, autism, and cancer [1,2,5,8,9,10,11,12,13,14,15]. The *KCNK9* gene-product TASK3 is a pH-regulated, potassium channel membrane protein that we, and others [29,30,31], show regulates mitochondrial membrane potential and apoptosis. This study provides the first demonstration that *KCNK9* is imprinted and monoallelically expressed in mammary epithelial cells. It also identifies a DMR that likely regulates imprinting at this locus in human breast tissue. In addition to the DMR identification, we demonstrated, by NOMe-Seq, a region of differential chromatin structure related to the methylation status of the DMR. The relationship between DNA methylation and condensed chromatin structure is consistent with a model in which methylation at the DMR silences gene expression by impacting chromatin accessibility and preventing transcription factor binding. This model also supports an epigenetic mechanism for *KCNK9*/TASK3 overexpression in breast cancer.

Hypomethylation of the *KCNK9* DMR was observed more frequently in African-American women with TNBC (*p* = 0.006) and less frequently in European-American women with TNBC (*p* = 0.70). Hypomethylation of the *KCNK9* DMR was observed concurrently in TNBC and normal-appearing adjacent breast tissue. Abnormal *KCNK9* imprinting was associated with increased mitochondrial membrane potential in live TNBC cells and non-cancerous mammary epithelial cells from high-risk women (*p* < 0.001). These results are consistent with a study by Nagy et al. who showed that, in melanoma, TASK3 regulates apoptosis and mitochondrial function [30]. The finding that *KCNK9*-US1 DMR hypomethylation occurs preferentially in African-Americans suggests that *KCNK9*/TASK3 may provide a new target for prevention of TNBC.

While 50% of Ashkenazi European women with TNBC have a germline mutation of *BRCA1*, only 20% of African-American women with TNBC have a *BRCA1* mutation [42]. This indicates that other mechanism(s) beyond germline mutation of *BRCA1* are responsible for the etiology of TNBC in African-American women. African-American women experience disparities in income, access to care, and an unequal burden of environmental exposures [43]. Given that imprinting is dysregulated by poor nutrition and environmental toxicants, our findings provide a potential mechanistic link between disparities and TNBC in African-American women who do not have germline *BRCA1* mutations. A limitation of this study is that it was conducted in a single institution in a restricted number of women; multi-institutional testing with an expanded test set and validation set is required to validate *KCNK9*/TASK3 as a potential risk biomarker.

The hypomethylation at the *KCNK9* DMR was observed in both non-cancerous and cancerous breast tissue, but it is rare in the WBC of at-risk individuals. These findings indicate that epigenetic alterations occurring at the *KCNK9* locus do not typically form at the time of fertilization and implantation. In contrast, they are consistent with alterations occurring in later epigenetically vulnerable developmental windows. These windows include during tissue differentiation, early childhood, or puberty. Identification of hypomethylation of the *KCNK9* DMR in both breasts of at-risk individuals is indicative of a relatively early developmental change with large spatial distribution. Hypomethylation seen in only one breast would indicate epigenetic alteration occurring later in development, in a more specific location or cell type. The epialleles identified here are quantifiable markers for association studies between the environmental factors and the critical exposure timing that contribute to breast cancer risk.

The DMR methylation and NOMe-Seq chromatin data provide intriguing targets for future studies to better understand the origins and progression of TNBC. This will include further investigation of DMR methylation, chromatin structure, transcription factor binding, and gene/protein expression in this aggressive form of breast cancer. As TNBC has rapid-onset, aggressive growth, and resistance to treatment, patient survival could be improved through better classification of risk status, early detection, and better treatment targets. Furthermore, since genomic imprinting is dysregulated by poor nutrition and exposure to environmental toxicants, the results here provide support for the importance of good nutrition and a healthy environment in the prevention of TNBC.

Other recent work into the epigenetic regulation of *KCNK9* has identified long distance cis-interactions between the promoter CpG island of *KCNK9*, which was found to be hypomethylated, and the PEG13 DMR [44]. As mentioned previously, parent-of-origin specific methylation in the *KCNK9* CpG island was not detected (Table A1), so any parental-specific regulation by this interaction would be due solely to the PEG13 DMR. Thus, it will be important to determine how CpG methylation and chromatin structure in the *KCNK9* promoter influence long-range interactions with PEG13. Furthermore, it will be of interest to determine whether interactions between the *KCNK9*-US1 DMR and the *KCNK9* promoter are involved in gene regulation. Such increased understanding of the epigenetic regulation of *KCNK9* will be of great value in determining the role that expression of this gene has in the development and progression of cancers and in developing new treatment methods.

Our studies highlight the importance of therapeutically targeting TASK3. While there has been a recent emphasis on precision medicine, it is unclear whether the benefits of precision medicine will impact women of color. Here we identify a target that has specific promise for African-American women. TASK3 can be targeted by natural products such as hydroxy-α-sanshool (active agent in Szechuan peppers) [45]. Most promising, a recent study showed that antibodies targeting the *KCNK9* protein inhibited 410.4 cell breast cancer cell metastasis in the mouse [46]. The high frequency of abnormal *KCNK9* imprinting in both TNBC and adjacent non-cancerous breast tissue provides evidence that *KNCK9* may serve both as a target for precision therapy and chemoprevention of TNBC in African-American women.

## 5. Conclusions

TASK3 is overexpressed in >40% of breast cancers, but genomic amplification of *KCNK9* is reported to occur in <10% of breast cancers [24], indicating that epigenetic mechanisms may play a key role in TASK3 overexpression. Herein, we show that *KCNK9* is imprinted and monoallelically expressed in mammary epithelial cells. We identify (1) a DMR that likely regulates imprinting at this locus and (2) by NOMe-Seq a region of differential chromatin structure that is determined by methylation status of the DMR. The relationship between DNA methylation and condensed chromatin structure is consistent with a model in which methylation at the DMR silences gene expression by impacting chromatin accessibility and preventing transcription factor binding.

Less than 20% of African-American women with TNBC have a *BRCA1* mutation [42]. This indicates that other mechanism(s) beyond germline mutation of *BRCA1* may be responsible for the etiology of TNBC in African-American women. Given that imprinting is dysregulated by poor nutrition and environmental toxins, our findings provide a potential mechanistic link between disparities and TNBC in African-American women who do not have germline *BRCA1* mutations. Hypomethylation at the DMR, coupled with biallelic expression of *KCNK9*, occurred in 75% of TNBC. The association between hypomethylation and TNBC status was highly significant in African-Americans (*p* = 0.006), but not in Caucasians (*p* = 0.70). *KCNK9* hypomethylation was also found in non-cancerous tissue from women at high risk of developing breast cancer. The high frequency of abnormal *KCNK9* imprinting in both TNBC and adjacent non-cancerous breast tissue provides evidence that *KNCK9* has the potential to serve both as a target for precision therapy and chemoprevention of TNBC in African-American women.

## Figures and Tables

**Table 1:** Methylation of the 5 consecutive CpG sites in the *KCNK9*-US1 DMR.

Individual—Tissue Type	CpG Site 1	CpG Site 2	CpG Sites 3 and 4	CpG Site 5
Ind1—Brain	0.66	0.7	0.32	0.34
Ind2—Brain	0.68	0.84	0.34	0.45
Ind3—Breast	0.58	0.57	0.33	0.35
Ind4—Breast	0.3	0.5	0.23	0.2
Ind5—Breast	0.55	0.66	0.27	0.3
Ind6—Liver	0.42	0.48	0.57	0.5
Ind7—Liver	0.41	0.46	0.48	0.34
Ind8—Testis	0.67	0.74	0.5	0.44

Values indicate the proportion of methylated cytosines, as measured by Sequenom MassArray. The value for sites 3 and 4 is an average of both cytosines, as they are in the same cleavage fragment.

**Table 2:** Association between breast cancer subtype and *KCNK9*-US1 DMR methylation in mammary epithelial cells and white blood cells.

Patient Number	Age (Years)	Race	BRCA Mutation	Cancer Subtype	Methylation MEC	Methylation WBC
1	39	C	0	TNBC	+	+
2	41	AA	0	TNBC	−	ND
3	34	AA	0	TNBC	−	+
4	47	AA	0	TNBC	−	ND
5	42	AA	ND	TNBC	−	ND
6	52	C	1	TNBC	−	+
7	38	AA	1	TNBC	−	+
8	49	C	ND	TNBC	−	ND
9	52	C	ND	TNBC	−	+
10	52	AA	0	TNBC	+	ND
11	43	AA	0	TNBC	−	+
12	49	C	ND	TNBC	+	ND
13	42	AA	ND	TNBC	−	ND
14	46	C	ND	TNBC	+/−	ND
15	50	AA	ND	TNBC	−	ND
16	50	AA	ND	TNBC	−	ND
17	38	C	1	TNBC	+/−	+
18	39	C	0	TNBC	+	+
19	55	C	ND	TNBC	+/−	++
20	41	AA	0	TNBC	−	ND
21	47	AA	0	TNBC	−	ND
22	42	AA	ND	TNBC	−	ND
23	45	C	0	TNBC	+	ND
24	36	C	0	TNBC	+/−	ND
25	36	C	ND	HER2+	+	+/−
26	40	C	ND	HER2+	+	+
27	42	C	0	HER2+	−	+
28	49	AA	0	HER2+	+	+
29	42	AA	ND	HER2+	+	ND
30	50	C	ND	HER2+	+/−	ND
31	53	AA	ND	HER2+	+/−	ND
32	39	C	ND	HER2+	+	+
33	36	C	ND	HER2+	+	+/−
34	49	AA	ND	HER2+	−	ND
35	47	AA	ND	HER2+	−	ND
36	47	C	ND	ER+	++	+
37	35	L/C	2	ER+	+	+
38	52	C	ND	ER+	−	++
39	34	AA	ND	ER+	++	+
40	42	AA	ND	ER+	+	ND
41	51	AA	ND	ER+	−	+/−
42	48	C	ND	ER+	++	ND
43	35	C	0	ER+	+	+
44	48	C	ND	ER+	−	ND
45	36	C	ND	ER+	−	ND
46	28	C	ND	ER+	+	ND
47	50	AA	ND	ER+	+	ND
48	53	C	ND	ER+	−	ND
49	47	C	ND	ER+	++	+
50	35	C	ND	ER+	++	ND
51	42	AA	ND	ER+	+	ND
52	49	C	ND	ER+	−	ND
53	53	C	ND	ER+	−	ND

(AA) African-American/Black, (C) European-American/White/Caucasian, (L) Latina, (0) No *BRCA1/2* mutation, (1) *BRCA1* mutation, (2) *BRCA2* mutation, (ND) Not Determined, (TNBC) Triple-negative breast cancer, (ER+) Estrogen receptor positive breast cancer, (HER2+) HER2/NEU overexpressing breast cancer, (DMR) Differentially methylated region, (−) Hypomethylation of *KCNK9* DMR methylation (0–20% methylation), (+/−) Partial hypomethylation of *KCNK9* DMR methylation (20–74% methylation), (+) Normal *KCNK9* DMR methylation (75–125% methylation), (++) Hypermethylation of *KCNK9* DMR (>125% methylation), (MEC) Microdissected mammary epithelial cells from breast cancer biopsy specimens, (WBC) White blood cells. Subjects are grouped by breast cancer subtype. Different colored backgrounds are used to group data.

**Table 3:** Analysis of *KCNK9*-US1 DMR methylation in mammary epithelial cells and white blood cells obtained from women ranging in age from 36 to 53.

#	Age (Years)	Race	BRCA Mt	Mutation	Cancer Subtype	Masood R-MEC	Masood L-MEC	Methylation R-MEC	Methylation L-MEC	Methylation WBC
1	47	C	ND		ER+	13	13	++	++	+
2	53	C	ND		NO	12	14	+	+	++
3	39	C	ND		HER2+	20	18	+	+/−	+
4	52	C	1	IVS5-11T > G	TNBC	15	22	+	−	+
5	43	AA	ND		NO	16	14	+	−	+
6	38	C	1	M1775R	TNBC	15	12	+/−	+/−	+
7	52	C	ND		NO	19	15	−	−	++
8	52	C	ND		NO	11	13	ND	+	+
9	39	C	0		TNBC	16	15	+	ND	+
10	34	L/C	2	6872del4	ER+	21	15	+	ND	+
11	52	C	ND		TNBC	16	ND	+/−	ND	++
12	36	C	ND		HER2+	18	18	+	ND	+/−
13	55	A	2UV	G2961S	NO	ND	15	ND	+/−	+/−
14	55	C	1	exon 22 del 510 bp	NO	14	13	ND	−	+/−
15	51	AA	ND		ER+	14	ND	−	ND	+/−
16	49	AA	0		TNBC	18	ND	−	ND	+/−
17	34	AA	0		TNBC	14	23	ND	−	+
18	43	AA	0		TNBC	14	20	ND	−	+
19	46	C	0		NO	9	15	ND	ND	++
20	48	C	ND		ER+	16	ND	ND	ND	++
21	50	C	ND		TNBC	14	ND	ND	ND	++
22	40	C	ND		NO	12	13	ND	ND	+
23	53	C	ND		NO	10	13	ND	ND	+
24	51	C	ND		NO	9	9	ND	ND	+
25	51	C	ND		NO	13	11	ND	ND	+
26	39	AA	ND		NO	ND	9	ND	ND	+
27	49	AA	ND		HER2+	17	18	ND	ND	+
28	47	AA	ND		HER2+	18	ND	ND	ND	+
29	49	C	ND		ER+	23	ND	ND	ND	+
30	40	C	ND		NO	16	15	ND	ND	+
31	41	AA	ND		NO	14	13	ND	ND	+
32	27	C	0	Rad50/R365Q (1094G > A)	TNBC	15	ND	ND	ND	+/−
33	45	AA	ND		NO	13	ND	ND	ND	−
34	35	C	ND		ER+	15	15	+	++	ND
35	42	C	0		NO	12	11	+	−	ND
36	47	C	1	Missing	NO	14	13	−	+/−	ND
37	51	C	1	Missing	NO	12	14	−	−	ND
38	41	AA	0		TNBC	16	17	−	−	ND
39	47	AA	0		TNBC	15	19	−	−	ND
40	42	AA	ND		TNBC	18	16	−	−	ND
41	45	C	ND		TNBC	19	19		+	ND
42	36	C	ND		TNBC	16	ND	+/−	ND	ND
43	42	AA	ND		ER+	16	20	+	+/−	ND
44	49	C	ND		ER+	16	20	−	ND	ND
45	53	C	0		ER+	16	23	−	ND	ND

Number of samples available for analysis: 1–7: MEC from both breasts and WBC; 8–18: MEC from one breast and WBC; 19–33: WBC only; 34–45: MEC only, from one or both breasts. (AA) African-American, (C) Caucasian, (L) Latina, (A) Asian, (0) No *BRCA1/2* mutation, (1) *BRCA1* mutation, (2) *BRCA2* mutation, (ND) Not Determined, (TNBC) Triple-negative breast cancer, (ER+) Estrogen receptor positive breast cancer, (HER2+) HER2/NEU overexpressing breast cancer, (NO) No cancer, (R) Right breast, (L) Left breast, (DMR) Differentially methylated region, (−) Hypomethylation of *KCNK9*-US1 DMR (0–20% methylation), (+/−) Partial hypomethylation of *KCNK9*-US1 DMR (20–74% methylation), (+) methylation of the *KCNK9*-US1 DMR (75–125% methylation), (++) Hypermethylation of the *KCNK9*-US1 DMR (>125% methylation), (MEC) Microdissected mammary epithelial cells from breast cancer biopsy, (WBC) White blood cells. Different colored backgrounds are used to group data.

## Data Availability

Data are contained within this article and in Supplementary Materials.

## Supplementary Materials

The following are available online at https://www.mdpi.com/article/10.3390/cancers13236031/s1, The uncropped blots and molecular weight markers are shown in Supplementary Materials.

## Appendix A

### Materials and Methods

Methylation Primers: Primers used for analysis of the KCNK9-US1 upstream methylation site were KCNK9-US1f, KCNK9-US1r, KCNK9-US2f, and KCNK9-US2r, which were used for DMR screening. Clone amplification and sequencing were performed using KCNK9-US0 and KCNK9-US4. All primers were synthesized by Sigma–Aldrich (St. Louis, MO, USA). Primer sequences were:

KCNK9-US1f: 5′-AGGAAGAGAGATATTTAAATGGTAAGGGTAGAG-3′;
KCNK9-US1r:5′-CAGTAATACGACTCACTATAGGGAGAAGGCTAACCCCCAAAAAACTAAT- AA-3′;
KCNK9-US2f: 5′-AGGAAGAGAGGGTTTGGGTTAAGATTTTTAAGAT-3′;
KCNK9-US2r:5′-CAGTAATACGACTCACTATAGGGAGAAGGCTAAAAATTCTTCCTTCCTTC- CA-3′;
KCNK9-US0: 5′-TATGGGAAAGGCTAAGGGAA-3′;
KCNK9-US4: 5′-AAATTCTTCCTTCCTTCCACCTTTA-3′.

Primers for Sequencing of KCNK9 Amplicons. Primers were synthesized by Sigma–Aldrich. Primer sequences were:

TM1R: 5′-ATGCAGTCGCTATAGCCTGACATGGTCAAGAACCTGAGGAC-3′;
KCDX-1F: 5′-CCTTCTACTTTGCGATCACGGTCATCACCAC-3′;
KCDX-3F: 5′-TCATCACCACCATAGGTTATGGGCACGCTGCACC-3′;
KCDX-3R: 5′-CCAGGATATACATAAAGCTAAAGGCCACGTAGAGC-3′;
KC-S2: 5′-CAAGGCCTTCTGCATGTTCTA-3′.

KCNK9 Cloning Primers: (IDT, Coralville, IA, USA) with BamHI ends were:

F: 5′-GGATCCGCCATGAAGAGGCAGAACGTG-3′
R: 5′-GGATCCCACGGACGCCTCTCTTGAATG-3′.

KCNK9-G95E site-directed mutagenesis primers (IDT) were:

F: 5′-CACCACCATAGAGTATGGGCACGC-3′
R: 5′-GCGTGCCCATACTCTATGGTGGTG-3′.

KCNK9 mRNA quantitation primers (Sigma–Aldrich) were:

KCNK9-F: 5′-GCTCCTTCTACTTTGCGATCACG-3′;
KCNK9-R: 5′-CTGGAACATGACCAGTGTCAGC-3′;
18S rRNA-F: 5′-GTAACCCGTTGAACCCCATT-3′;
18S rRNA-R: 5′-CCATCCAATCGGTAGTAGCG-3′.

Methylation analysis: CpG methylation was measured by analysis of bisulfite converted DNA. Sodium bisulfite modification of DNA was performed using EpiTect Bisulfite Kits (Qiagen) according to the manufacturer’s instructions, converting unmethylated cytosines to uracil. Quantitative methylation for DMR identification was measured by Sequenom MassARRAY with EpiTYPER analysis software (Sequenom, San Diego, CA, USA). Sequenom EpiDesigner software was used for primer design, incorporating a T7 promoter site to forward primers, and tags to the reverse primers, to balance the annealing temperatures. PCR was performed using HotStarTaq (Qiagen). PCR products were processed using Sequenom MassCLEAVE reagents to deactivate unincorporated dNTPS, transcribe the amplicons from the T7 promoters, and cleave the transcripts with RNAse A. Incorporation of dTTP in the transcription reactions restricts RNase A cleavage to cytosines. The fragmented transcripts were spotted onto SpectroCHIPS for mass spectrometry analysis on the MassARRAY instrument. EpiTYPER software identified fragments based on predicted mass, calculating ratios for fragments of different mass resulting from conversion/non-conversion of cytosines at CpG sites, reporting this ratio as the methylation fraction.

Semiquantitative methylation analysis in screening patient samples for methylation changes was completed using dye terminator sequencing (BigDye, Applied Biosystems, ThermoFisher Scientific, Waltham, MA, United States) on amplicons of the DMR region. For CpG sites 3 and 4, signal intensities of the overlapping C and T peaks were compared to classify the methylation level: C intensity at 75–125% of the T signal was designated “full methylation” (+), C intensity ranging from undetectable to 20% of the T signal was designated “full hypomethylated” (−), peak intensity from 20–74% was designated “partial hypomethylation” (+/−), and peak intensity greater than 125% was designated “hypermethylated” (++).

Contiguous and allele specific methylation were determined by sequencing the clone’s bisulfite DNA PCR products. Amplicons were cloned using a TOPO-TA cloning kit, with the pCR4 sequencing vector and Top 10 chemically competent cells (Life Technologies). Single transformed colonies were chosen and the inserts amplified and sequenced by BigDye 3.1 sequencing reagents (Applied Biosystems).

DNA extraction: Total DNA was isolated from RPFNA samples and enzymatically dispersed normal mammary epithelial cell samples by washing and resuspending the cells in 500 µL 10 mM Tris (pH 7.4), 10 mM NaCl, 3 mM MgCl_2_, and 0.1 mM EDTA [47,48]. The cells were then incubated overnight with 50 µL proteinase K (Qiagen) at 55 °C, followed by phenol-chloroform extraction and ammonium acetate/isopropanol precipitation of the DNA with glycoblue coprecipitant (Life Technologies).

Total DNA was isolated from conceptus tissues using buffer ATL, proteinase K, and RNase A (Qiagen), followed by phenol–chloroform extraction and ethanol precipitation.

NOMe-Seq: For in vitro methylation, tissues were homogenized in a nuclei homogenization buffer (approximately 50 mg tissue in 500 µL 10 mM Tris (pH 7.4), 10 mM NaCl, 3 mM MgCl_2_, and 0.1 mM EDTA) plus protease inhibitors (Protease Inhibitor Cocktail and PMSF, Sigma–Aldrich). Samples were kept on ice for 10 min, Igepal (Sigma–Aldrich) was added to 1%, samples were vortexed, and nuclei collected by centrifugation. Nuclei were then resuspended in 500 µL M.cviPI reaction buffer and incubated with 100 U M.cviPI at 37 °C for 15 min (New England Biolabs, Ipswich, MA, USA). Then, 50 µL proteinase K (Qiagen) was added and samples incubated overnight at 55 °C. Samples were extracted with phenol:chloroform:isoamyl alcohol and DNA precipitated with ammonium acetate and isopropanol.

Monoallelic expression analysis: Determination of mono- and biallelic gene expression was performed by cDNA sequencing. Commercially obtained breast genomic DNA (Biochain, Newark, CA, USA) was obtained for analysis. Tissues were genotyped at rs2615374 using Applied Biosystems components and equipment, according to the manufacturer’s standard protocols; TaqMan Made to Order Assay C_2776566_20, TaqMan Genotyping Master Mix, and amplification and analysis were performed on an Applied Biosystems 7900HT Fast Real-Time PCR System (Applied Biosystems). Informative samples heterozygous at rs2615374 were selected for expression analysis. Commercially purified, matched DNAs and RNAs from normal breast tissue were purchased from Biochain (Hayward, CA, USA). RNAs from tissues were purified using RNA-Stat 60 (Tel-Test, Inc., Friendswood, TX, USA), according to the manufacturer’s instructions. Reverse transcription was performed using Quantitect-RT kits (Qiagen), according to the manufacturer’s modified protocol for gene specific primers, using primer TM1R at a final concentration of 0.7 µM. TM1R is based on M1R from Luedi et al. [21], using the M1R transcript specific sequence with the addition of a non-genomic sequence as an amplification tag. Using primers specific to this non-genomic tag controls against amplification of contaminating genomic DNA or aberrantly primed anti-sense RNA.

*KCNK9* cDNA was amplified from brain tissue with a single round of amplification using TM1R and M1F primers. Amplification from breast tissue required two rounds of amplification with nested primers, using the TwistDX recombinase amplification kit (TwistDX, Ltd., Cambridge, UK). The first round used primers TM1R and KCDX-1F primers, and the second, primers KCDX-3F and KCDX-3R. All primer pairs used span the ~85 kb single intron of *KCNK9* (Figure 1B).

Sequencing of the amplicons was performed using ABI BigDye 3.1 (Applied Biosystems), primers M1R and M1F for brain cDNA, and primers KCDX-3R and KC-S2 for breast cDNA.

Cell Lines: Cell lines were obtained from either the American Type Culture Collection (Manassas, VA, USA) or the Duke Cell Culture Facility (Durham, NC, USA). Cells were validated by short tandem repeat DNA profile analysis and used within 15 passages. MDA231 and HEK293 cells were grown in Dulbecco’s modified eagle medium (DMEM) and the media was supplemented with 10% fetal bovine serum. SUM149 and SUM225 cells were grown in F12 media supplemented with 5% fetal bovine serum, 10 mM HEPES, 1 µg/mL of hydrocortisone (Sigma–Aldrich), and 5 µg/mL of human insulin. SUM190 cells were grown in the same media without fetal bovine serum. HMEC15 cells (Lonza, Walkersville, MD, USA) were immortalized with human telomerase. The hTERT immortalized HMEC15 and DKAT cells were grown in HuMEC media supplemented with bovine pituitary extract and HuMEC supplement. MCF10A cells were grown in F12/DMEM media supplemented with 5% horse serum, 20 ng/mL EGF, 10 µg/mL insulin, 0.5 µg/mL hydrocortisone, and 100 ng/mL cholera toxin (Sigma–Aldrich). All media and supplements were from GIBCO (Life Technologies) unless otherwise noted. All cell lines were tested for mycoplasma prior to the initiation of these studies.

SDS-PAGE and western analysis: SDS-PAGE was performed using Novex precast 4–12% Bis-TRIS gels and the MES running buffer system (Life Technologies). Separated proteins were transferred to a PVDF membrane (BioRad, Hercules, CA, USA), the membrane blocked with 5% BSA in 0.1% TTBS and then incubated with the primary antibody indicated in blocking solution overnight at 4 °C. The membrane was then washed with 0.1% TTBS, incubated for 1 h with secondary antibody, rewashed with 0.1% TTBS, and imaged with a Kodak IS 2000MM (Carestream, Norwalk, CT, USA) using Pierce SuperSignal Dura reagent (Thermo Fisher).

*KCNK9* mRNA quantitation: The HMEC15 cell line was seeded at a density of 4.2 × 10^4^ cells/well in a six-well plate with complete media. After 24 h, cells were treated with 1.0 or 2.5 µM of 5-aza-2′-deoxycytidine or vehicle for up to 72 h. After treatment, cells were harvested for real-time qPCR analysis. Total RNA was isolated using the RNeasy Mini Kit (Qiagen) and included an on column DNase digestion treatment according to the manufacturer’s protocol. Total RNA was converted to cDNA using the qPCRBIO cDNA synthesis kit (PCR Biosystems, Wayne, PA, USA) according to the manufacturer’s protocol. QPCR was performed using the Apex qPCR Green Master Mix (Apex Bioresearch) on the QuantStudio 5 Real-Time PCR system (Applied Biosystems). Experiments were performed three times.

MTT assay: Cells were seeded at a density of 2.5 × 10^3^ cells/well (five wells/treatment) in a 96-well plate with complete media. After 24 h, cells were treated with 1.0 or 2.5 µM of 5-aza-2′-deoxycytidine (Sigma–Aldrich) or vehicle control for up to 72 h with fresh medium added after 24 h. After treatment, cell supernatant was replaced with MTT (.5 mg/mL, Invitrogen) and incubated for an additional 4 h at 37 °C. MTT was replaced with DMSO and incubated for 20 min at room temperature on a shaker. Absorbance was measured at 560 nm and 750 nm using a microplate reader. Experiments were performed three times.

Apoptosis Assays: Cells were plated on day one and allowed to attach and resume growth. On day zero, cells were treated with staurosporine (0.25 µg/mL or 0.50 µg/mL) (Sigma–Aldrich), or an equal volume of dimethylsulfoxide (DMSO), and incubated for 4 h at 37 °C. Subsequently, the media was removed, and the cells were incubated for 30 min with 300 µL lysis buffer (AnaSpec, Fremont, CA, USA). Cells were then scraped and the supernatant collected and centrifuged at 2500× *g* for 10 min at 4 °C. The supernatant was assayed for caspase-3 activity by mixing 170 µL of supernatant with 57 µL of caspase-3 substrate solution (AnaSpec), and then incubating the mixture for 1 h at room temperature with shaking. Following incubation, fluorescence was measured with a Shimadzu 1501 fluorimeter (Shimadzu USA, Columbia, MD, USA) at λ_ex_ = 490 nm and λ_em_ = 520 nm with a 20 nm bandwidth. Experiments were performed in triplicate.

ΔΨ_M_ analysis of cell lines and live RPFNA cytology: The relationships between *KCNK9*-US1 DMR methylation and ΔΨ_M_ (measured by JC-1 R/G ratio) or Masood Score (a measure of cytologic abnormality) [36] were investigated in aspirated human TNBC cells from seven women and non-cancerous mammary epithelial cells from ten high-risk women. Seven women, including TNBC and high-risk women, were BRCA1 mutation carriers; bilateral breast tissue was available from 59% (10/17) of these women (Table A2). Quantitative DNA methylation analysis was performed and *KCNK9*-US1 DMR methylation was classified as methylation (75–125% methylation), partial hypomethylation (20–74%), or hypomethylation (0–20% methylation). Mammary epithelial cell cytology was classified by the Masood Score [36]. One-way ANOVA was used to determine the association between *KCNK9*-US1 DMR methylation status, and the continuous variables of ΔΨ_M_ and Masood Score.

On day -1, cells were seeded in a black 96 well microtiter plate at 20,000 cells per well in triplicate. On day 0, the media was removed and replaced with media containing 0.5 ug/mL JC-1 (ThermoFisher), and then the cells were incubated in the dark for 30 min at 37 °C. Next, the JC-1 containing media was removed, the cells were washed three times with warm PBS, and 200 mL of warm PBS was added to each well. Epithelial cells were identified by morphology. Red (λ_ex_ = 525 nm, λ_em_ = 580–640 nm) and green (λ_ex_ = 490 nm, λ_em_ = 510–570 nm) fluorescence were measured, and the ratio of red to green fluorescence was determined as a measure of ΔΨ_M_.

Human subjects and tissue collection: Informed consent was obtained from women at high risk for breast cancer who were scheduled for a breast surgical procedure in the operating room. RPFNA samples were obtained from the patient and transferred from the operating room to the laboratory within 5 min and immediately processed for analysis. RPFNA cytology samples were washed with phosphate buffered saline (PBS) and resuspended in 1 mL DMEM. Then, they were incubated with either JC-1 (final concentration: 1 µg/mL) or vehicle control at 37 °C for 30 min. After incubation, the cells were centrifuged, and the staining solutions were decanted. Each sample was washed twice with warm PBS and resuspended in 200 µL of warm PBS. The cell suspension was transferred to a black flat-bottom 96-well plate and measured as above. Epithelial cells were identified by morphology. DAPI (4′,6-diamidino-2-phenylindole) was used as a control and its fluorescence was measured (λ_ex_ = 365 nm, λ_em_ = 410–460 nm).

High risk was defined by (1) known BRCA mutation carrier, (2) a Gail Model Risk of >1.66% over five years, or (3) a strong family history of breast and/or ovarian cancer. A strong family history of breast and/or ovarian cancer risk was defined as having at least one of the following: (a) two or more first-degree relatives with breast cancer before the age of 50 years, (b) one first degree relative with bilateral breast cancer or ovarian cancer, (c) two or more first-degree relatives with breast cancer, (d) one first degree relative and two or more second or third degree relatives with breast cancer, (e) one first-degree relative with breast cancer and one or more relatives with ovarian cancer, (f) two second or third degree relatives with either breast cancer and one or more with ovarian cancer, (g) one second or third degree relative with breast cancer and two or more with ovarian cancer, or (h) three or more second or third degree relatives with breast cancer.

To control for hormonal effects on mammary cell proliferation, menstruating women were aspirated between days 1 and 12 of their cycle. The breast was anesthetized with 5 mL of 1% lidocaine, immediately adjacent to the areola, at ~3 and 9 o’clock positions. Eight to ten aspirations were performed per breast for random sampling of epithelial cells. After the aspiration, cold packs were applied to the breasts for 10 min, and both breasts were bound in kerlex gauze for 12 to 24 h. Epithelial cells were pooled and placed in modified CytoLyt (Cytyc Co., Boxborough, MA, USA) with 1% formalin for 24 h. Cells from the right and left breast were processed independently, so as to obtain one specimen per aspirated breast. Epithelial cells were split into two samples, with half designated for cytology and half designated for DNA extraction.

Cytology preparations were also given a semiquantitative index score through evaluation by the Masood cytology index. As previously described, cells were given a score of one to four points for each of six morphologic characteristics that include cell arrangement, pleomorphism, number of myoepithelial cells, anisonucleosis, nucleoli, and chromatin clumping.

**Table A1:** Methylation levels for CpG sites in the *KCNK9* promoter CpG island.

KCNK9 Promoter TF-Binding Site	Sample	C Kd	C Lv	C Br	Ad Brst	Ad Lv	Ad Br
Chr8:140717586-140718423	CpG sites	35	37	37	35	37	37
Forward – ATTTAGGTGACACTATAGAAATTTTAGTTAAGGAAGGGATGGAGA	Mean	0.069	0.093	0.08	0.062	0.074	0.058
Reverse – CAGTAATACGACTCACTATAGGGAGAAGGCTCATCTCAAAAATCCTTCCAATACTC	SD	0.044	0.054	0.078	0.064	0.10	0.059
	Median	0.06	0.09	0.05	0.04	0.04	0.04
	Range	0–0.15	0–0.23	0–0.32	0–0.34	0–0.58	0–0.29
KCNK9 CpG island Promoter-Exon 1	Sample	C Kd	C Lv	C Br	Ad Brst	Ad Lv	Ad Br
Chr8:140715033-140715433	CpG sites	4	4	ND	ND	ND	ND
Reverse – CAGTAATACGACTCACTATAGGGAGAAGGCTTACAAAATCACCAACTCCAACTACC	SD	0.012	0.052	ND	ND	ND	ND
	Median	0.005	0	ND	ND	ND	ND
	Range	0–0.03	0–0.11	ND	ND	ND	ND
KCNK9 CpG island Exon 1-Intron 1	Sample	C Kd	C Lv	C Br	Ad Brst	Ad Lv	Ad Br
Chr 8: 140714363-140714967	CpG sites	14	14	14	ND	ND	ND
Forward – ATTTAGGTGACACTATAGAAGTTTTAGAATTGGAATTTAGGGGAA	Mean	0.029	0.043	0.034	ND	ND	ND
Reverse – CAGTAATACGACTCACTATAGGGAGAAGGCTTCATCACCACCATAAATAAAAACTAAA	SD	0.021	0.054	0.036	ND	ND	ND
	Median	0.029	0.043	0.034	ND	ND	ND
	Range	0–0.07	0–0.2	0–0.12	ND	ND	ND
Reverse – CAGTAATACGACTCACTATAGGGAGAAGGCTTACAAAATCACCAACTCCAACTACC	SD	0.012	0.052	ND	ND	ND	ND

Methylation levels were measured by Sequenom MassArray with a possible value range from 0.0 to 1.0 (i.e., hypomethylated to hypermethylated); primers and amplicon positions are listed (GRCh37/hg19). Measurements were made in conceptus kidney (C Kd), liver (C Lv), brain (C Br) and adult breast (Ad Brst), liver (Ad Lv), and brain (Ad Br) tissues. The number of CpG sites listed per amplicon were those readable by MassArray analysis, with mean, standard deviation, median, and range of methylation given for each tissue. (ND) Not Determined.

**Table A2:** Association between Masood Cytology Index, R/G ratio, and *KCNK9*-US1 DMR methylation in mammary epithelial cells.

Number	Age (Years)	Race	BRCA Mut.	Mutation	Cancer Subtype	Masood R-MEC	Masood L-MEC	Methylation R-MEC	Methylation L-MEC	Methylation WBC
1	47	C	ND		ER+	13	13	++	++	+
2	53	C	ND		NO	12	14	+	+	++
3	39	C	ND		HER2+	20	18	+	+/−	+
4	52	C	1	IVS5-11T > G	TNBC	15	22	+	−	+
5	43	AA	ND		NO	16	14	+	−	+
6	38	C	1	M1775R	TNBC	15	12	+/−	+/−	+
7	52	C	ND		NO	19	15	−	−	++
8	52	C	ND		NO	11	13	ND	+	+
9	39	C	0		TNBC	16	15	+	ND	+
10	34	H	2	6872del4	ER+	21	15	+	ND	+
11	52	C	ND		TNBC	16	ND	+/−	ND	++
12	36	C	ND		HER2+	18	18	+	ND	+/−
13	55	A	2UV	G2961S	NO	ND	15	ND	+/−	+/−
14	55	C	1	exon 22 del 510 bp	NO	14	13	ND	−	+/−
15	51	AA	ND		ER+	14	ND	−	ND	+/−
16	49	AA	0		TNBC	18	ND	−	ND	+/−
17	34	AA	0		TNBC	14	23	ND	−	+
18	43	AA	0		TNBC	14	20	ND	−	+
19	46	C	0		NO	9	15	ND	ND	++
20	48	C	ND		ER+	16	ND	ND	ND	++
21	50	C	ND		TNBC	14	ND	ND	ND	++
22	40	C	ND		NO	12	13	ND	ND	+
23	53	C	ND		NO	10	13	ND	ND	+
24	51	C	ND		NO	9	9	ND	ND	+
25	51	C	ND		NO	13	11	ND	ND	+
26	39	AA	ND		NO	ND	9	ND	ND	+
27	49	AA	ND		HER2+	17	18	ND	ND	+
28	47	AA	ND		HER2+	18	ND	ND	ND	+
29	49	C	ND		ER+	23	ND	ND	ND	+
30	40	C	ND		NO	16	15	ND	ND	+
31	41	AA	ND		NO	14	13	ND	ND	+
32	27	C	0	Rad50-R365Q (1094G > A)	TNBC	15	ND	ND	ND	+/−
33	45	AA	ND		NO	13	ND	ND	ND	−
34	35	C	ND		ER+	15	15	+	++	ND
35	42	C	0		NO	12	11	+	−	ND
36	47	C	1	Missing	NO	14	13	−	+/−	ND
37	51	C	1	Missing	NO	12	14	−	−	ND
38	41	AA	0		TNBC	16	17	−	−	ND
39	47	AA	0		TNBC	15	19	−	−	ND
40	42	AA	ND		TNBC	18	16	−	−	ND
41	45	C	ND		TNBC	19	19		+	ND
42	36	C	ND		TNBC	16	ND	+/−	ND	ND
43	42	AA	ND		ER+	16	20	+	+/−	ND
44	49	C	ND		ER+	16	20	−	ND	ND
45	53	C	0		ER+	16	23	−	ND	ND

(AA) African-American, (C) Caucasian, (0) No BRCA1/2 mutation, (1) *BRCA1* mutation, (2) *BRCA2* mutation, (ND) Not determined, (TNBC) Triple-negative breast cancer, (ER+) Estrogen receptor positive breast cancer, (HER2+) HER2/NEU overexpressing breast cancer, (NO) No cancer, (R) Right breast, (L) Left breast, (DMR) Differentially methylated region, (−) Full hypomethylation of *KCNK9* DMR (0–20% methylation), (+/−) Partial hypomethylation *KCNK9* DMR (20–74% methylation), (+) Normal *KCNK9* DMR methylation (75–125% methylation), (++) Hypermethylation of *KCNK9* DMR (>125% methylation), (MEC) Mammary epithelial cells from breast RPFNA, (R/G Ratio) JC-1 red/green fluorescent ratio.
