# Supplementary material for: Epigenetic Dysregulation of KCNK9 Imprinting and Triple-Negative Breast Cancer
Source: Cancers (Basel). 2021 Nov 30;13(23):6031. doi: 10.3390/cancers13236031 (PMC8656495; doi:10.3390/cancers13236031)

Figure S1. Figure 4C – MCF10A – TASK 3

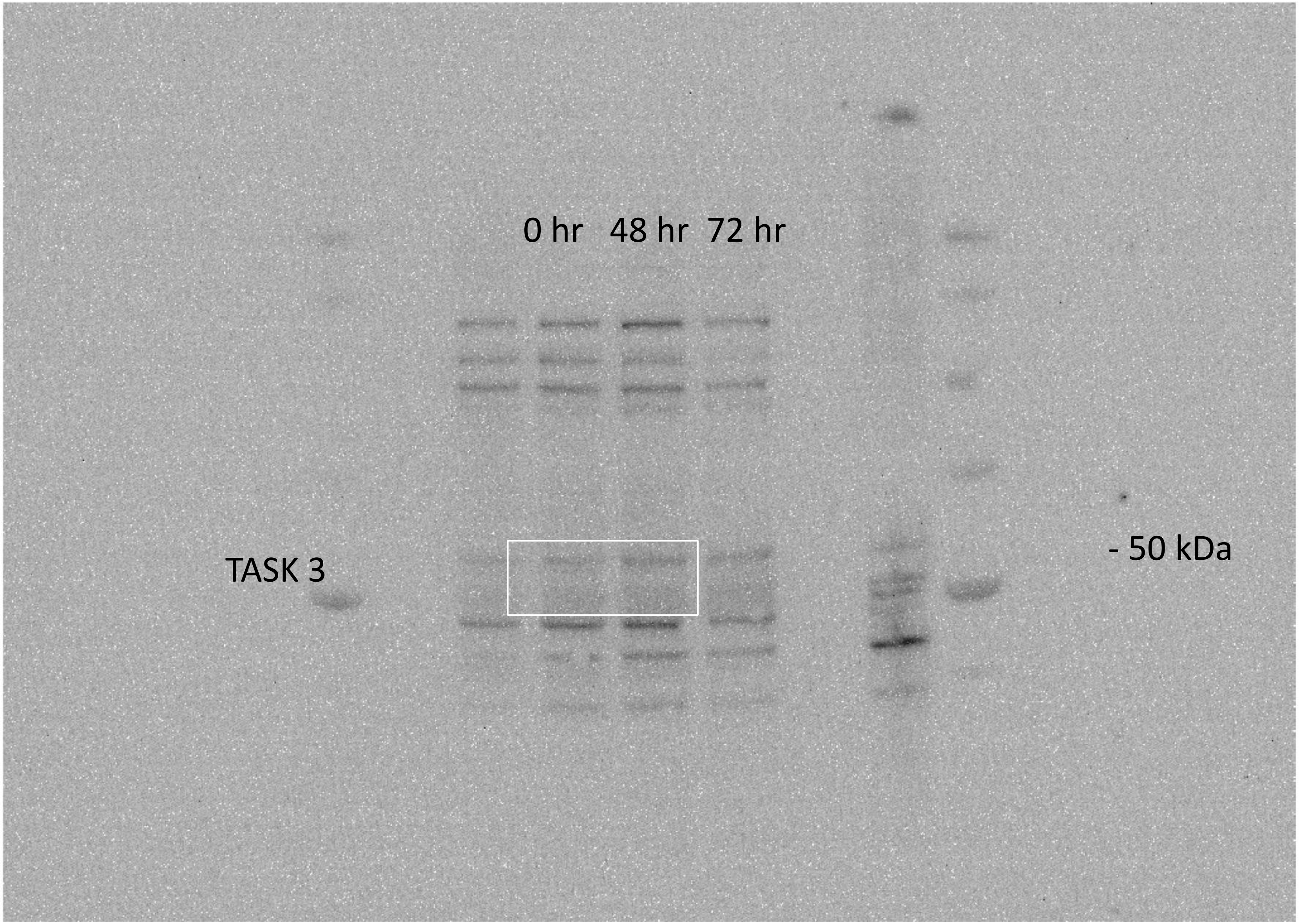

Figure S2. Figure 4C – MCF10A – GAPDH

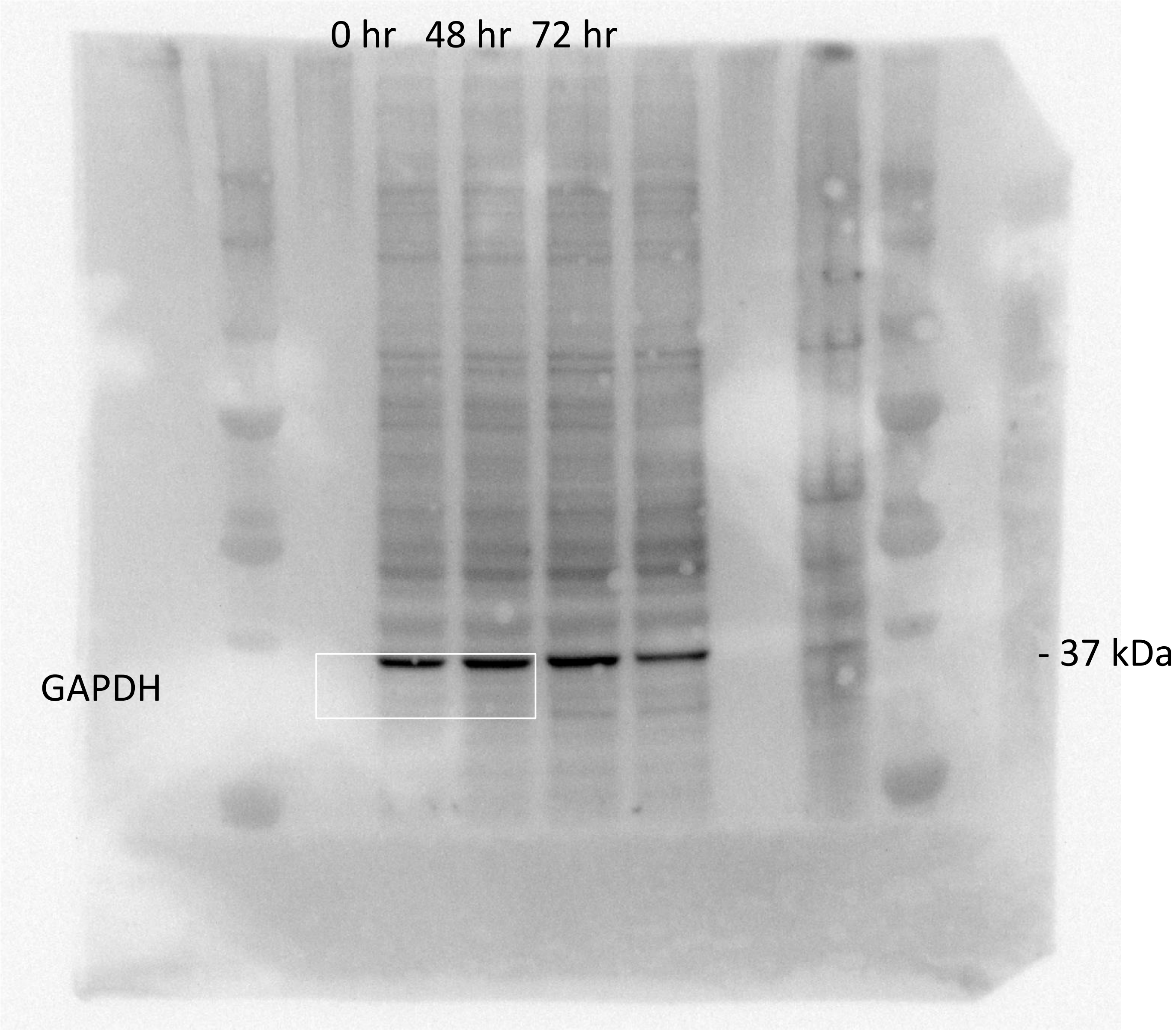

Figure S3. Figure 4C – HMEC15 Set 1 – Expt 2 – Uncropped TASK3 western

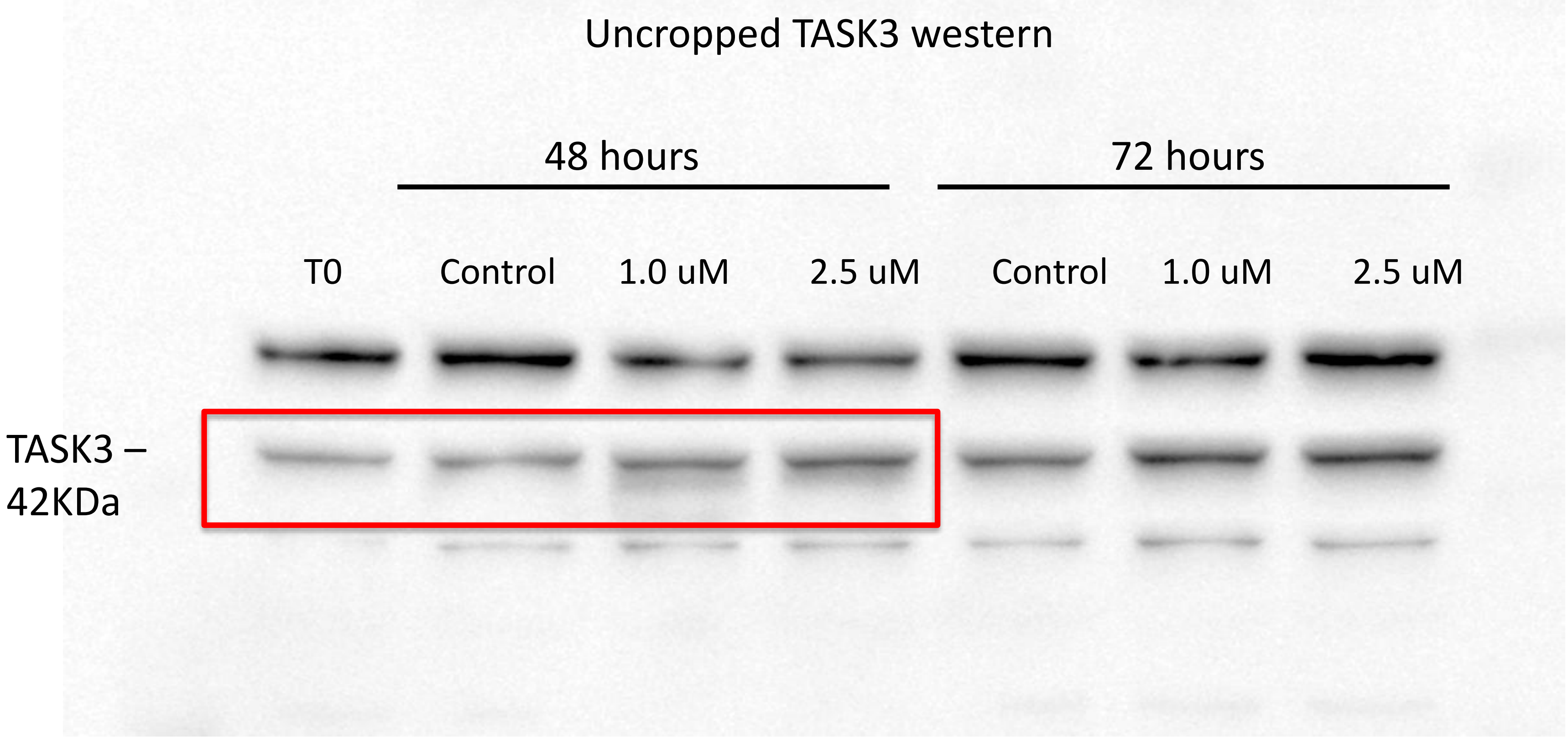

Figure S4. Figure 4C – HMEC15 Set 1 – Expt 2 – Uncropped TASK3 with ladder

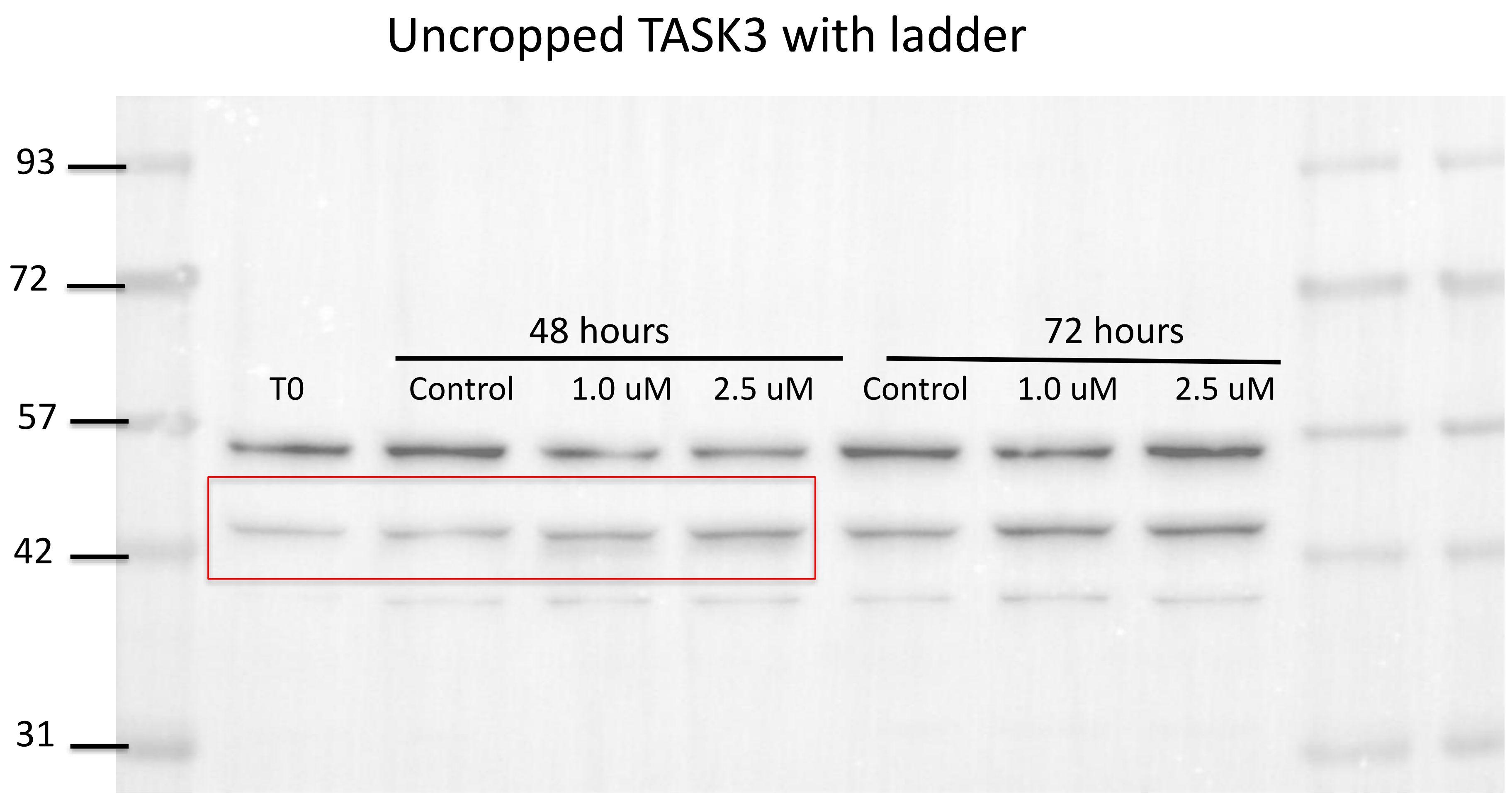

Figure S5. Figure 4C – HMEC15 – set 1 – loading control – Expt 2

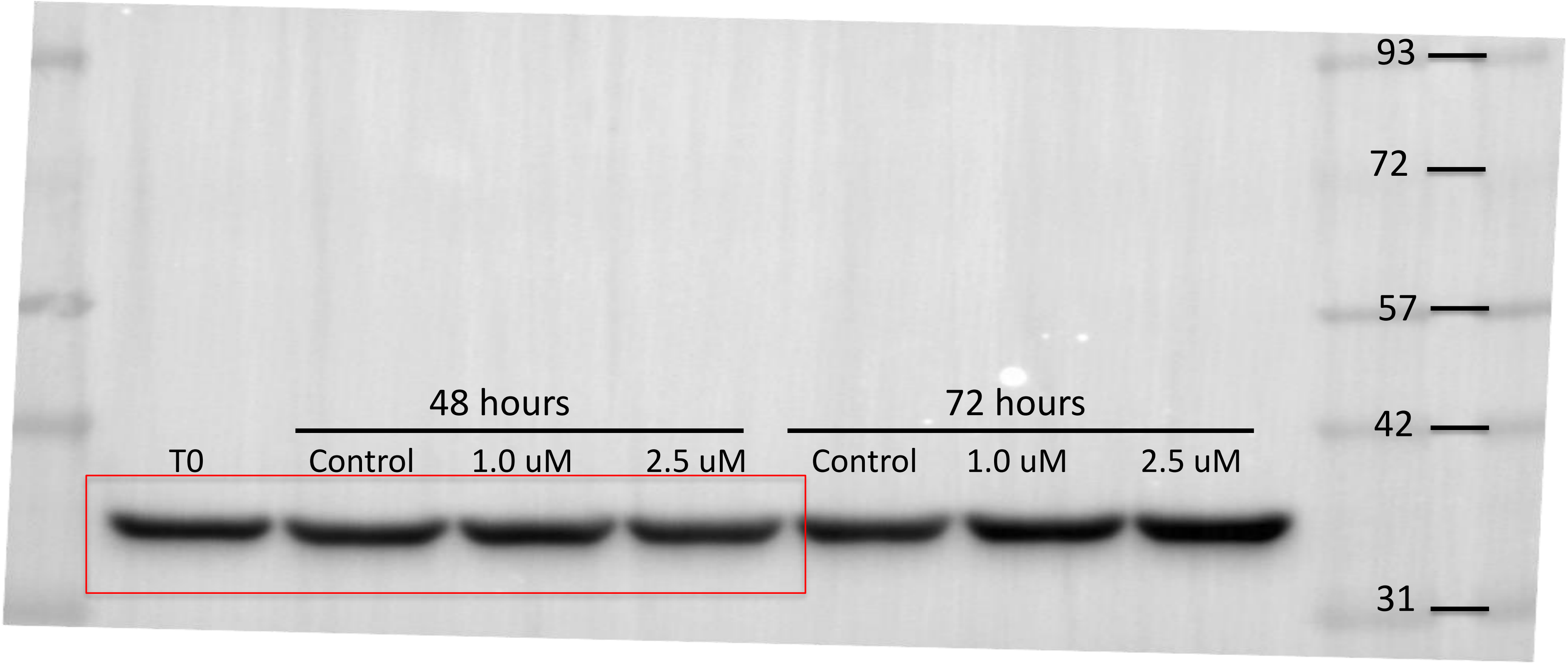

Figure S6. Figure 4C – HMEC15 – set 2 – Expt 1 – Uncropped TASK3 western

Uncropped TASK3 western

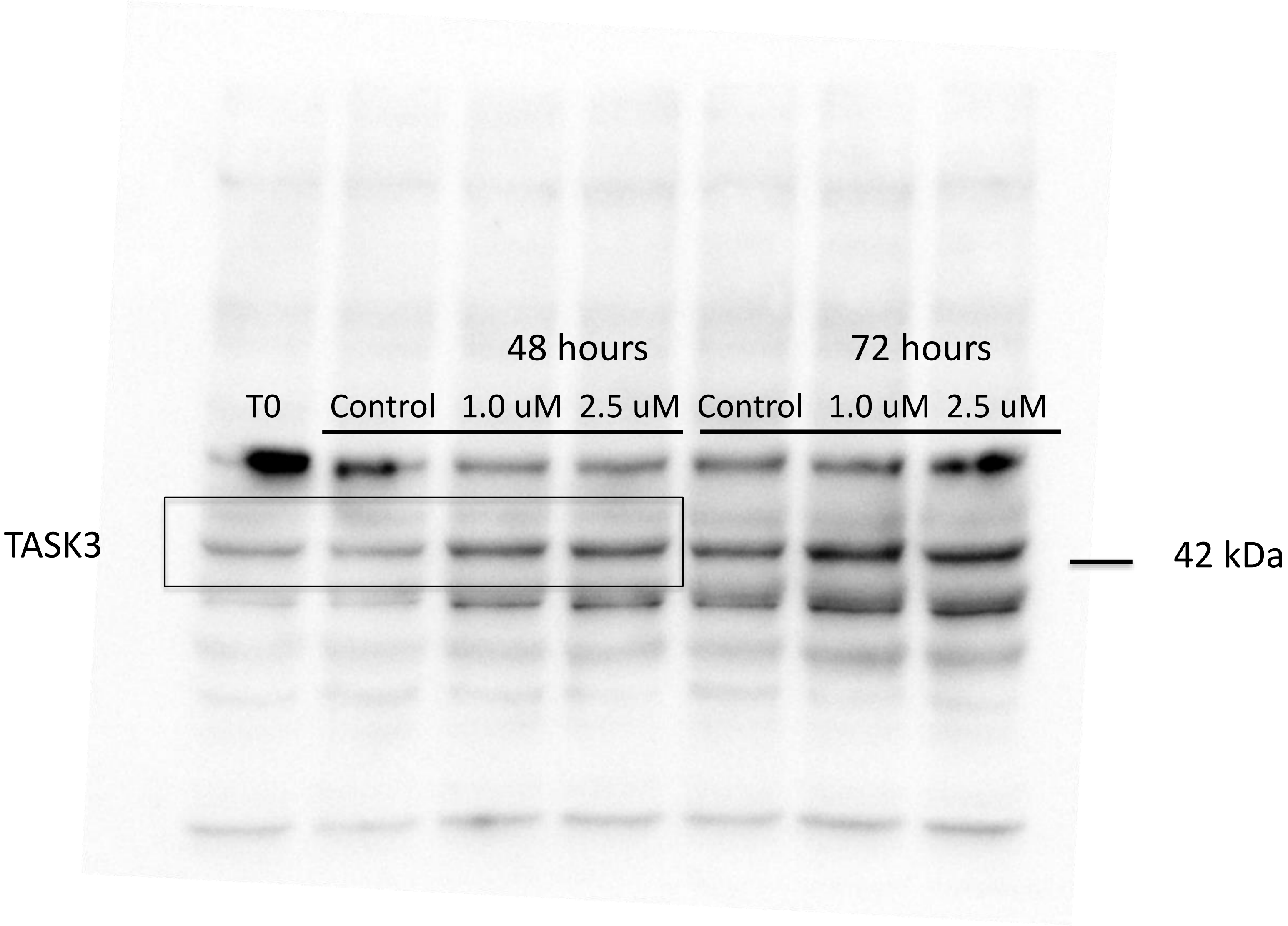

Figure S7. Figure 4C – HMEC15 – set 2 – Expt 1 – Uncropped TASK3 with ladder

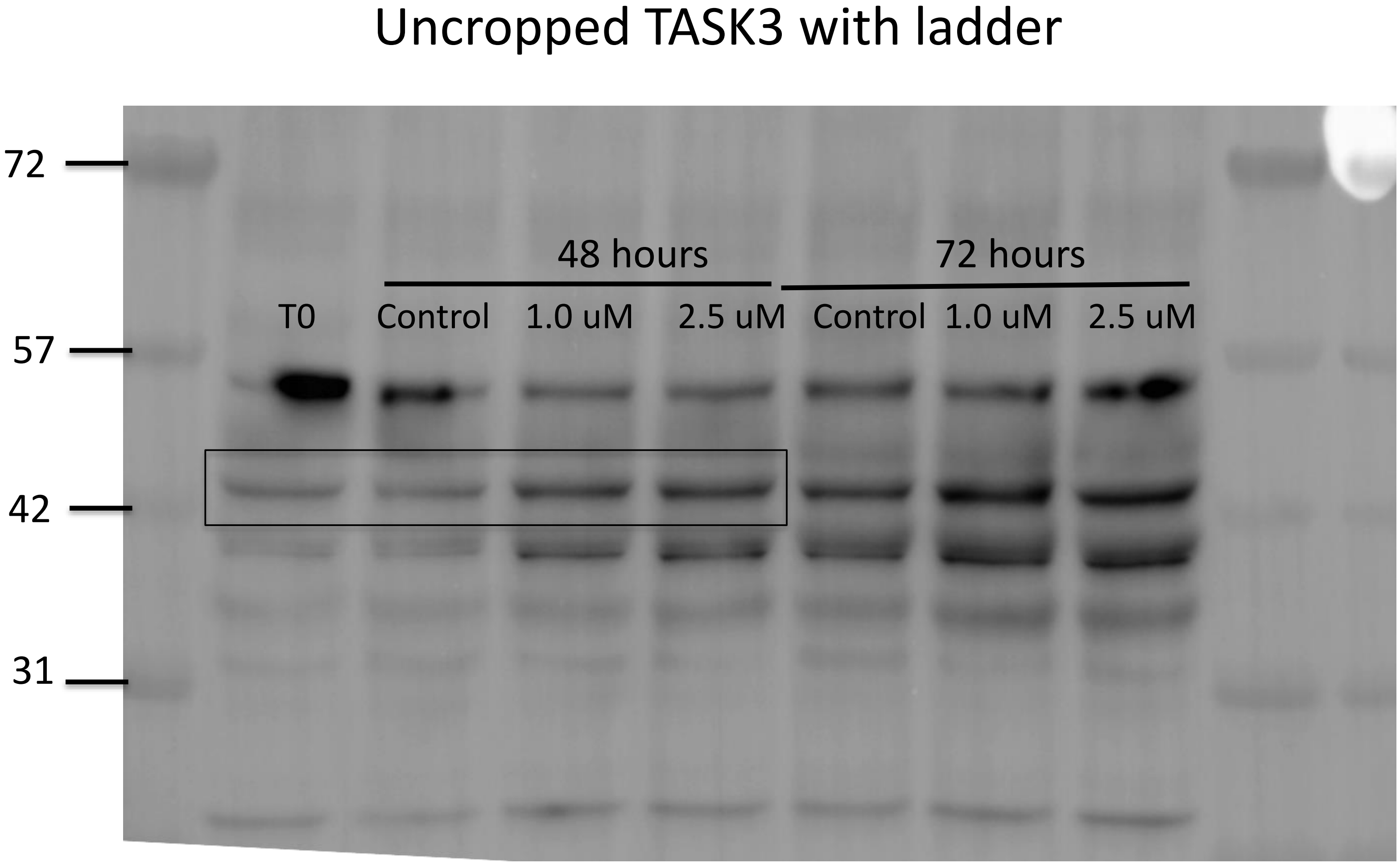

Figure S8. Figure 4C – HMEC15 – set 2 – Expt 1 – Uncropped GAPDH

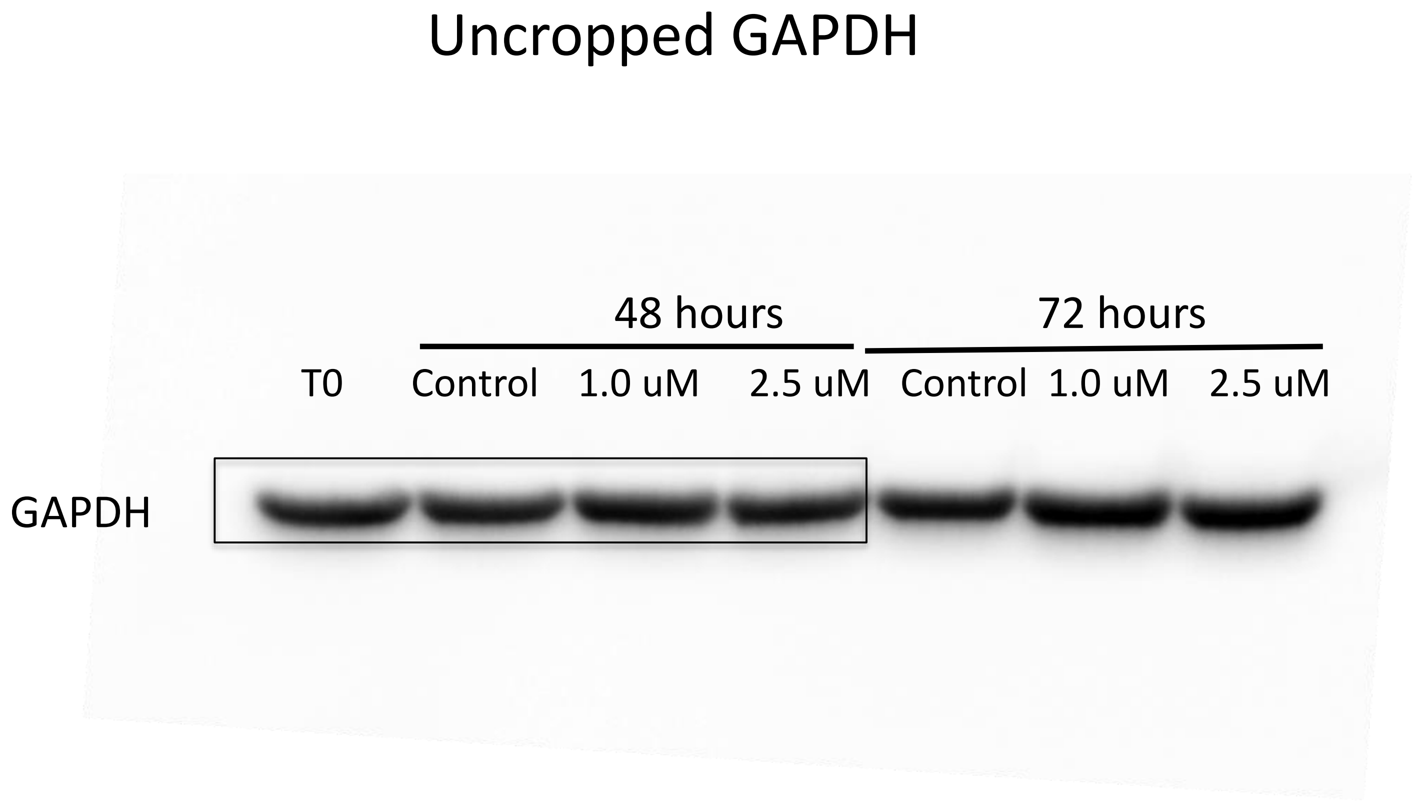

Figure S9. Figure 4C – HMEC15 – set 2 – Exp 1 – Uncropped GAPDH

Uncropped GAPDH

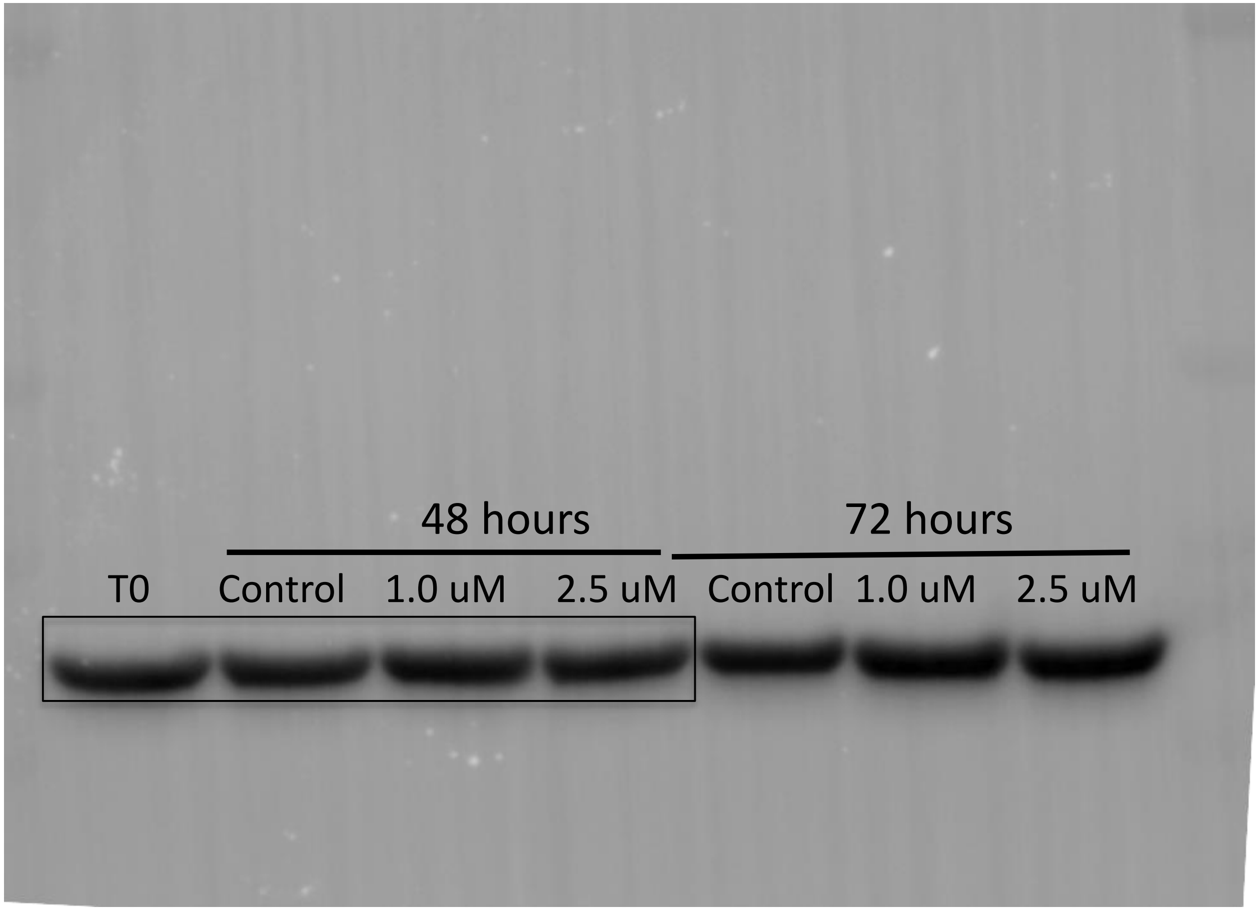

Figure S10. Figure 4C – HMEC15 – set 3 – Exp 3 – TASK3 original blot

TASK3 original blot

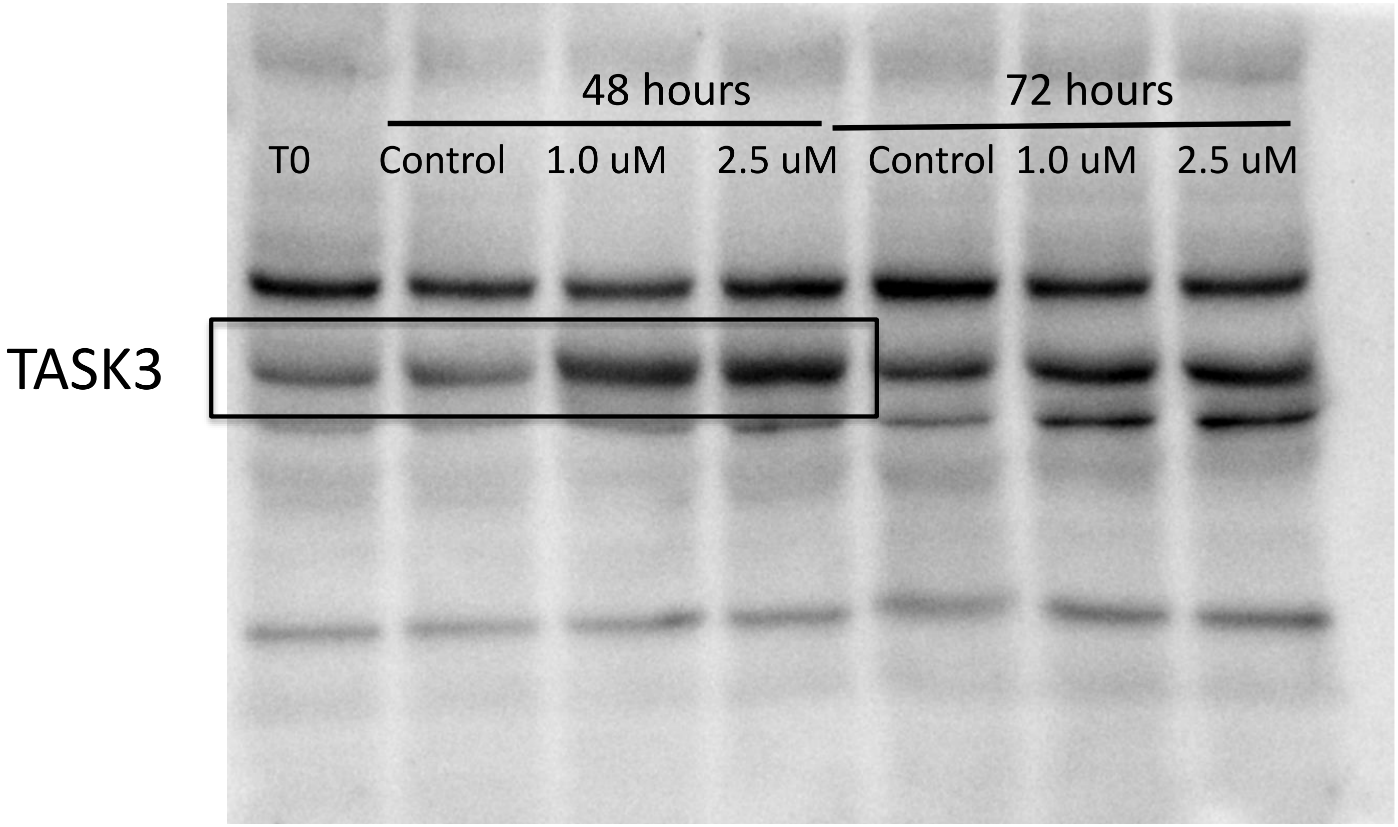

Figure S11. Figure 4C – HMEC15 – set 3 – Ext 3 – HMEC15 treated with 5-AzC for up to 48 hours, TASK3

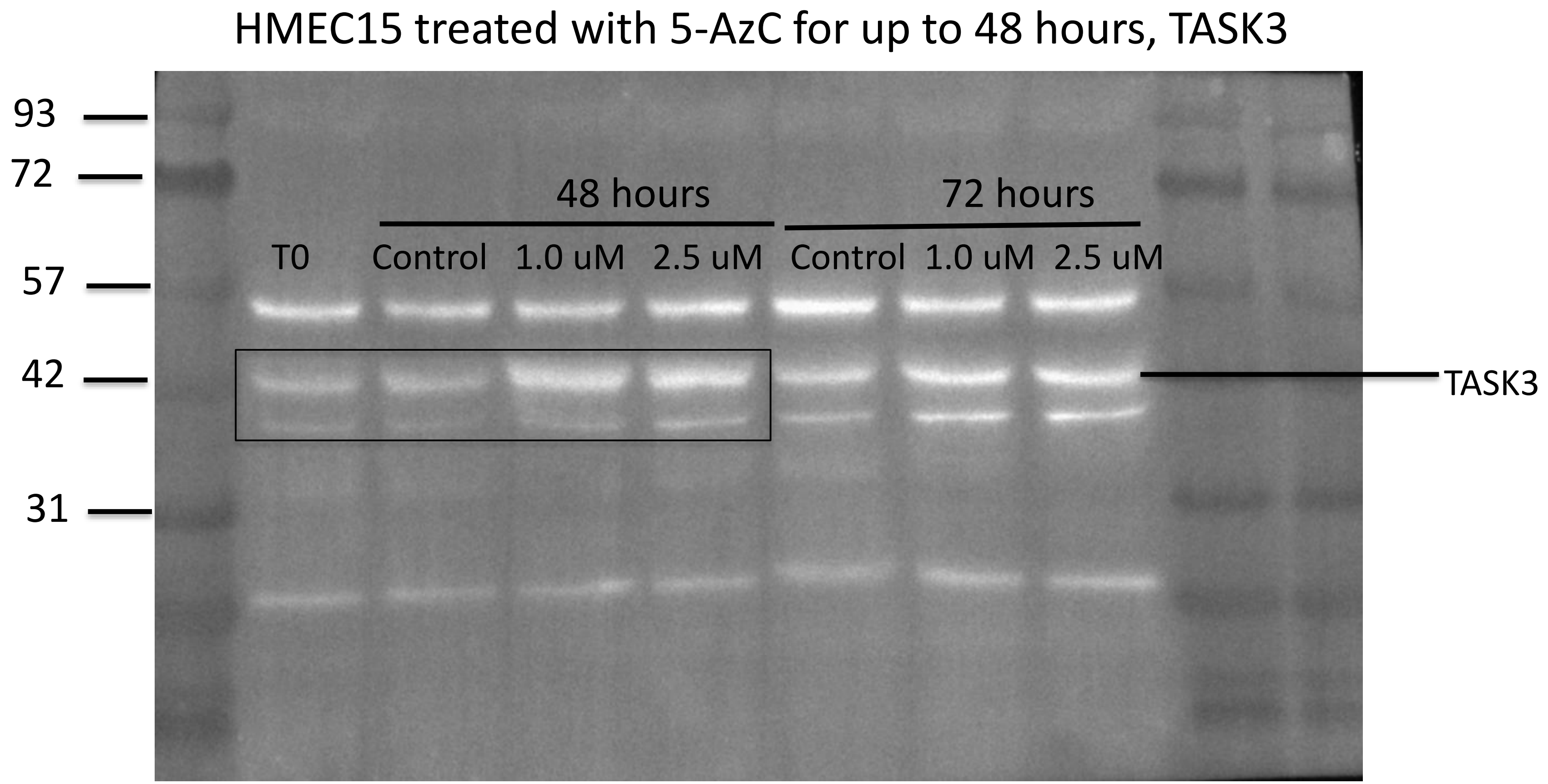

Figure S12. Figure 4C – HMEC15 – set 3 – Ext 3 – HMEC15 treated with 5-AzC for up to 48 hours, GAPDH

HMEC15 treated with 5-AzC for up to 48 hours, GAPDH

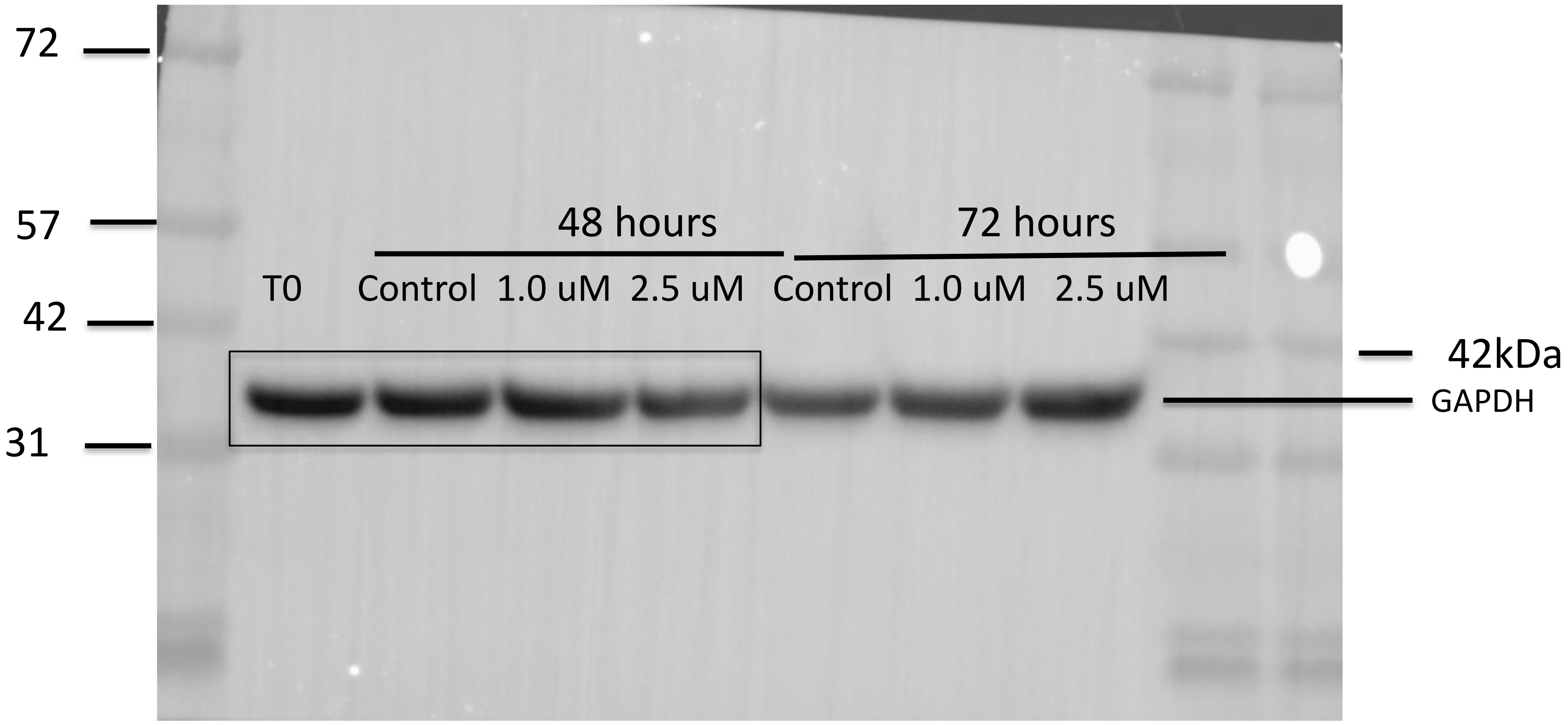

Figure S13. Figure 4C– HMEC15 – set 3 – Expt 3 – GAPDH original blot

GAPDH original blot

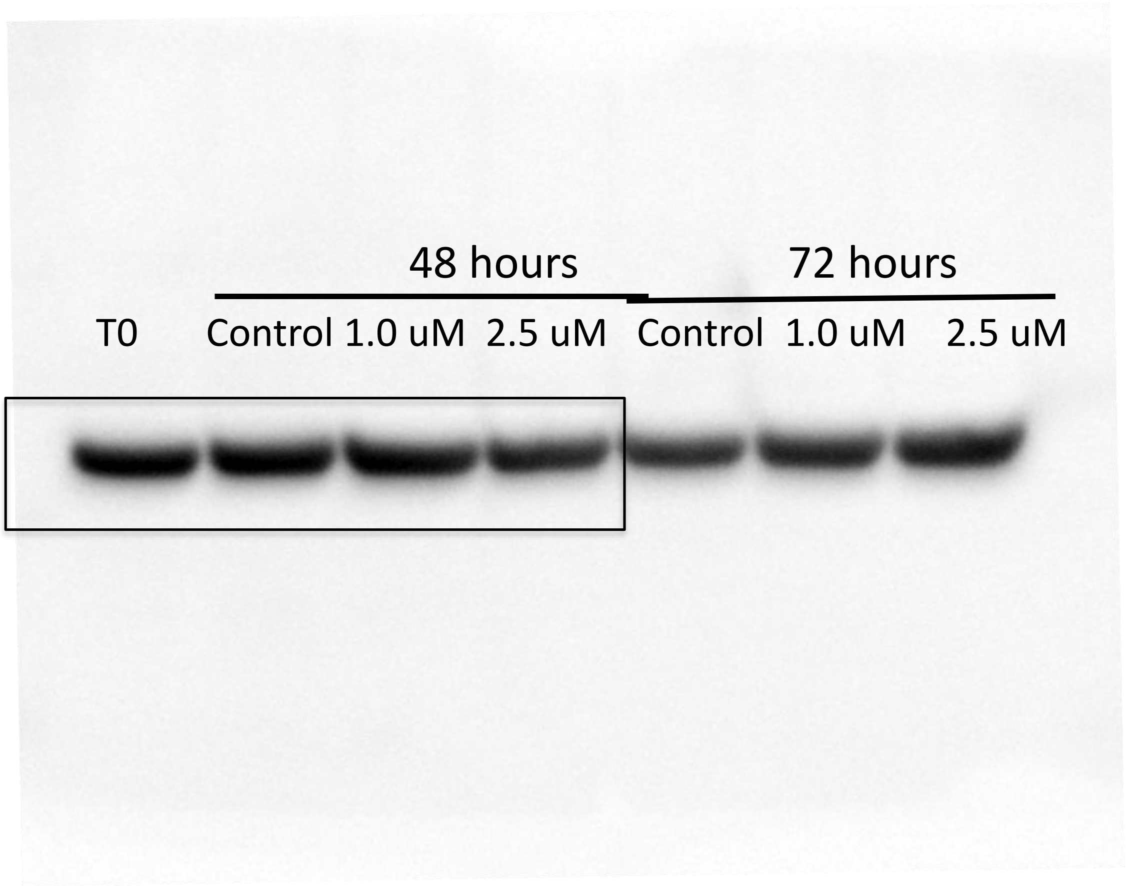

Figure S14. Figure 5A

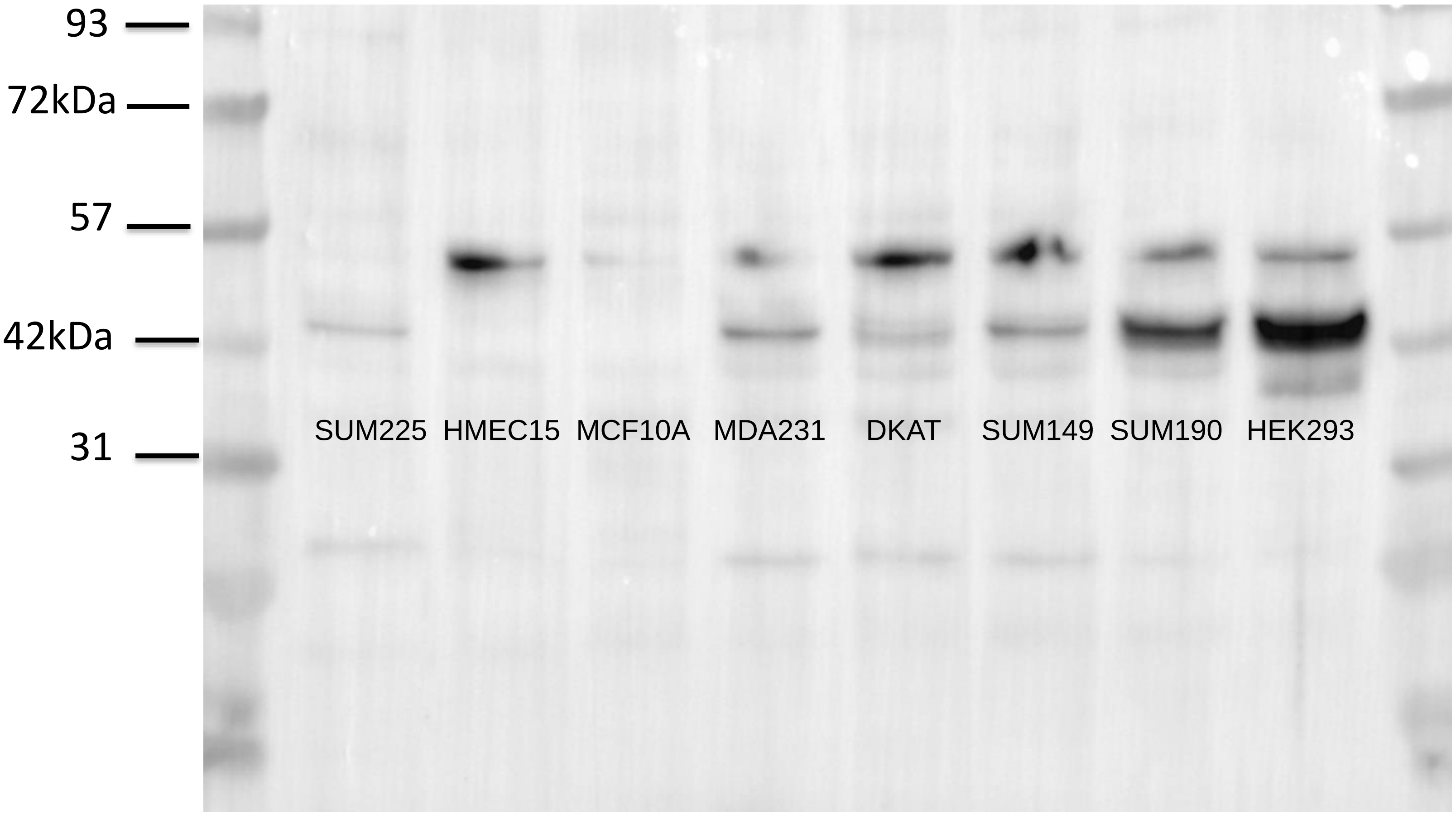

Figure S15. Figure 5A – GAPDH

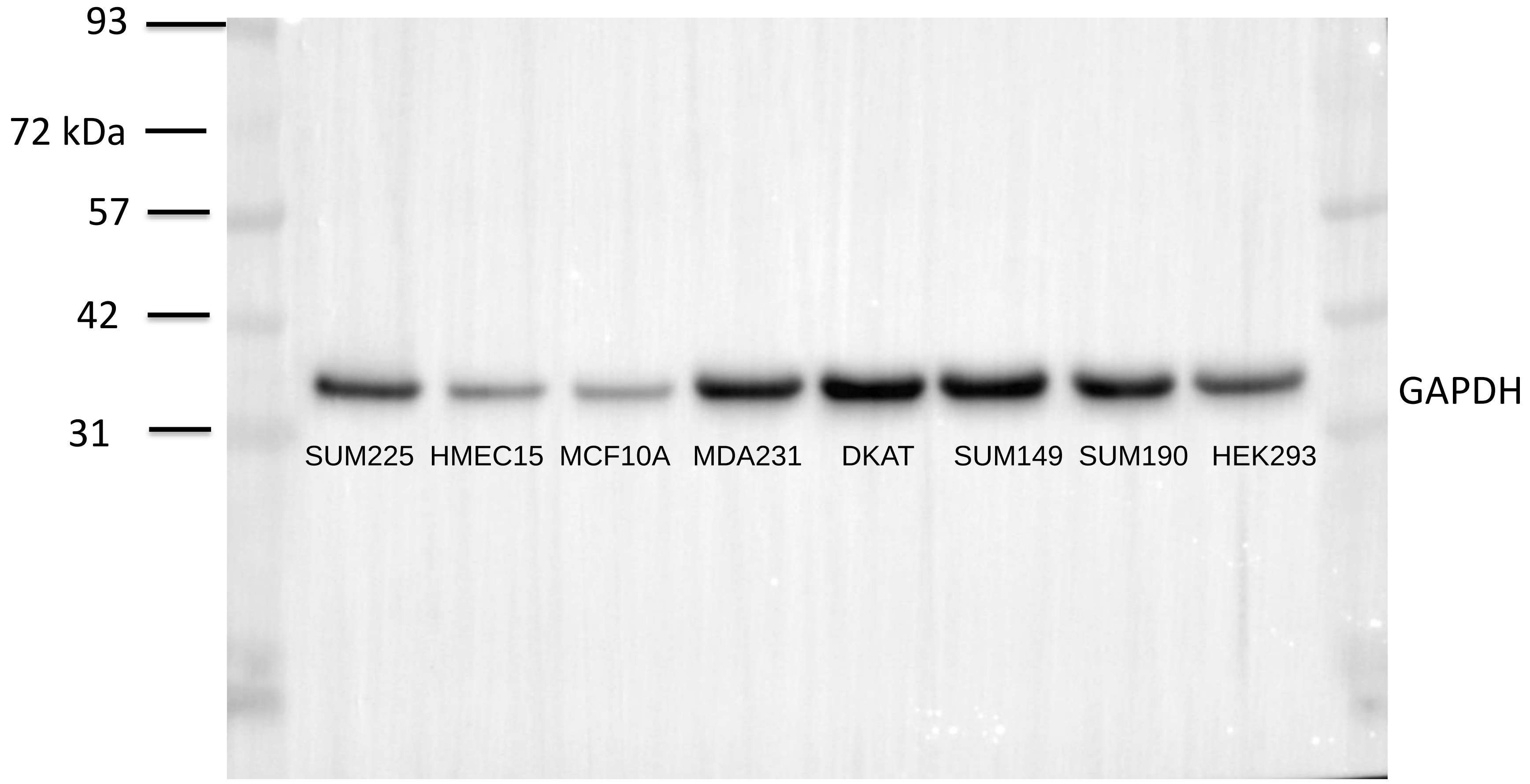

Figure S16. Current Figure 5B and former Figure 6A – TASK3 Flag

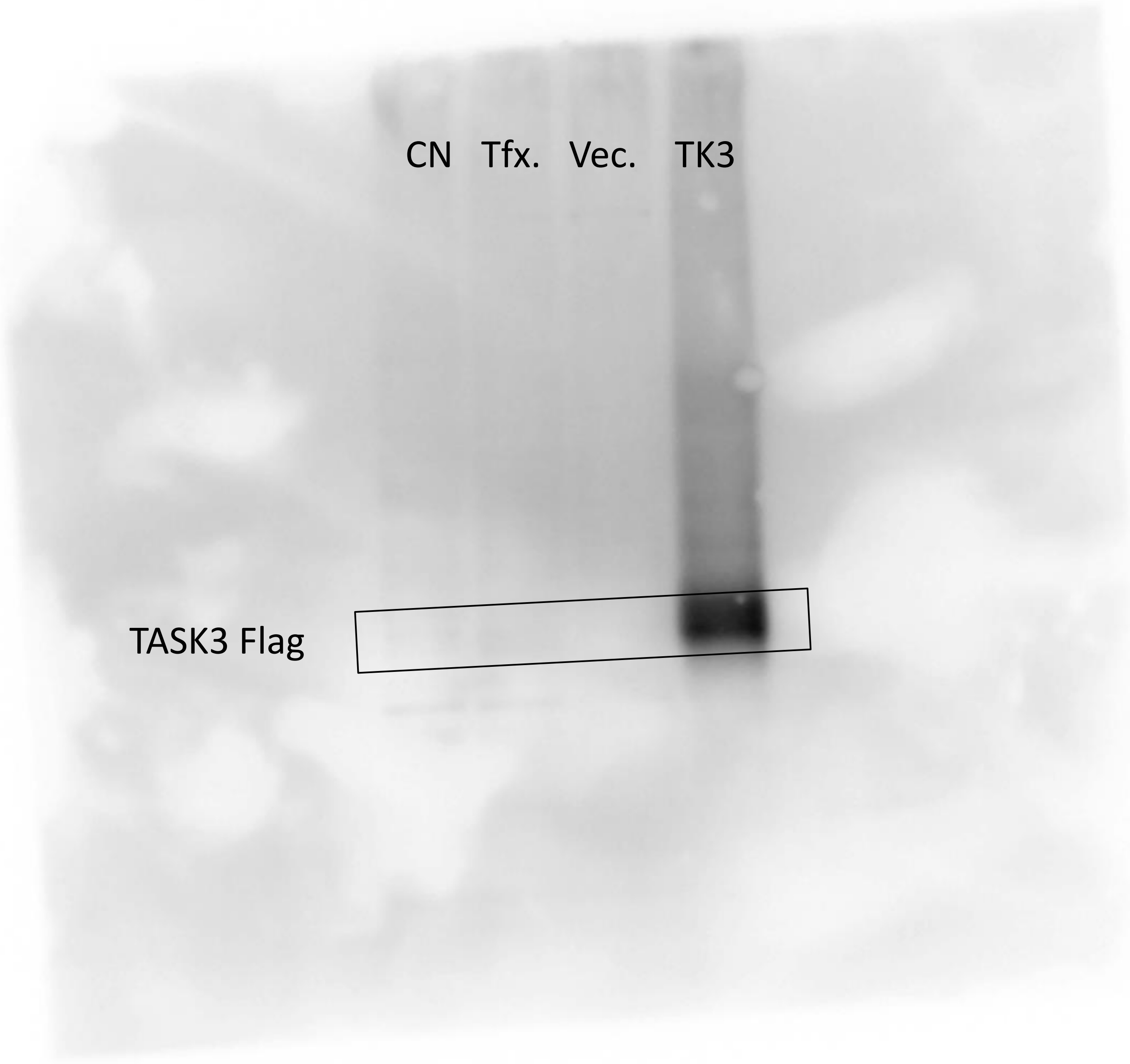

Figure S17. Current Figure 5B and former Figure 6A – TASK3 – 42KDa

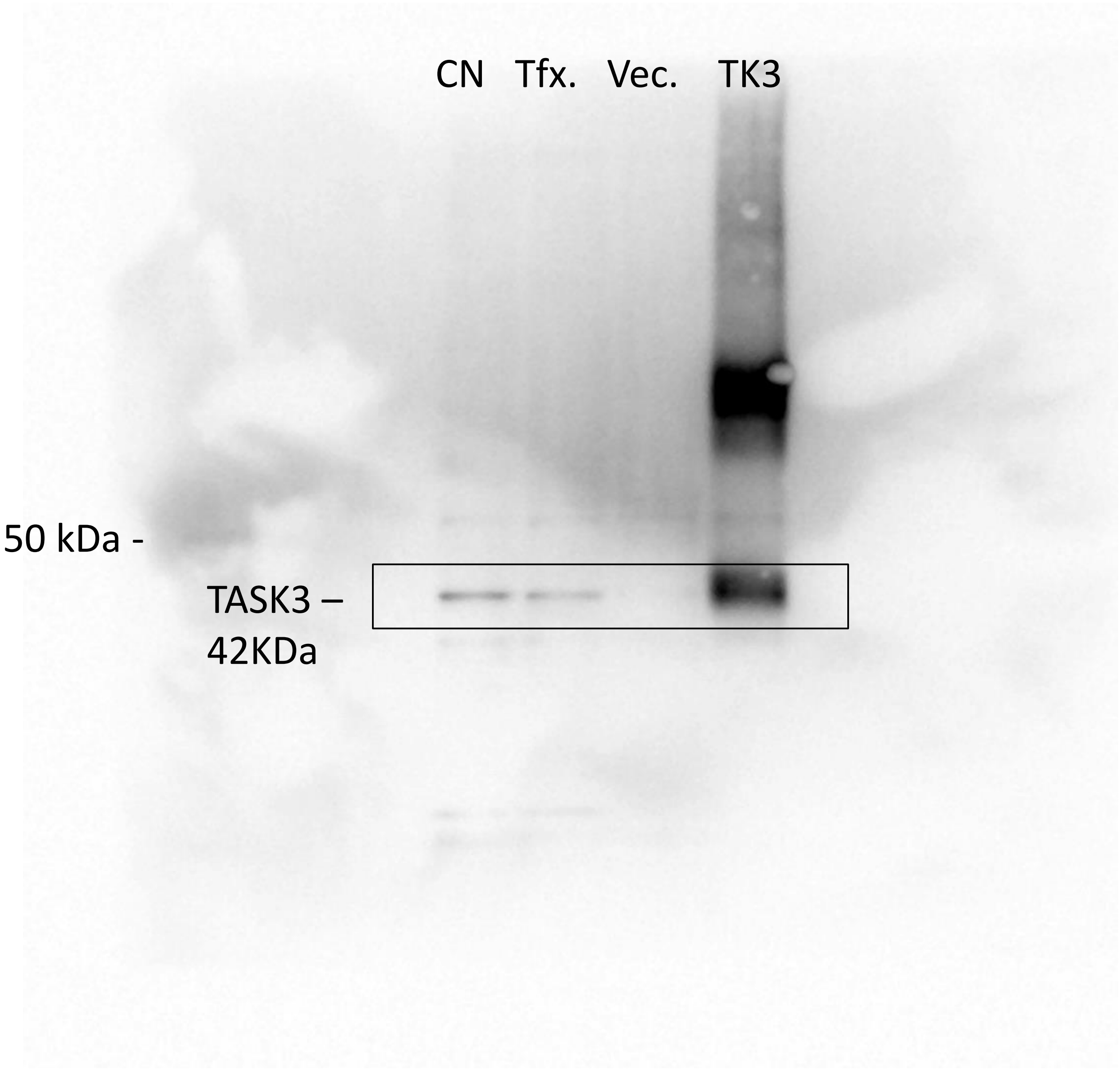

Figure S18. Figure 5C – expt 1 – MCF10A Blobe – KCNK9 Flag-Tag +

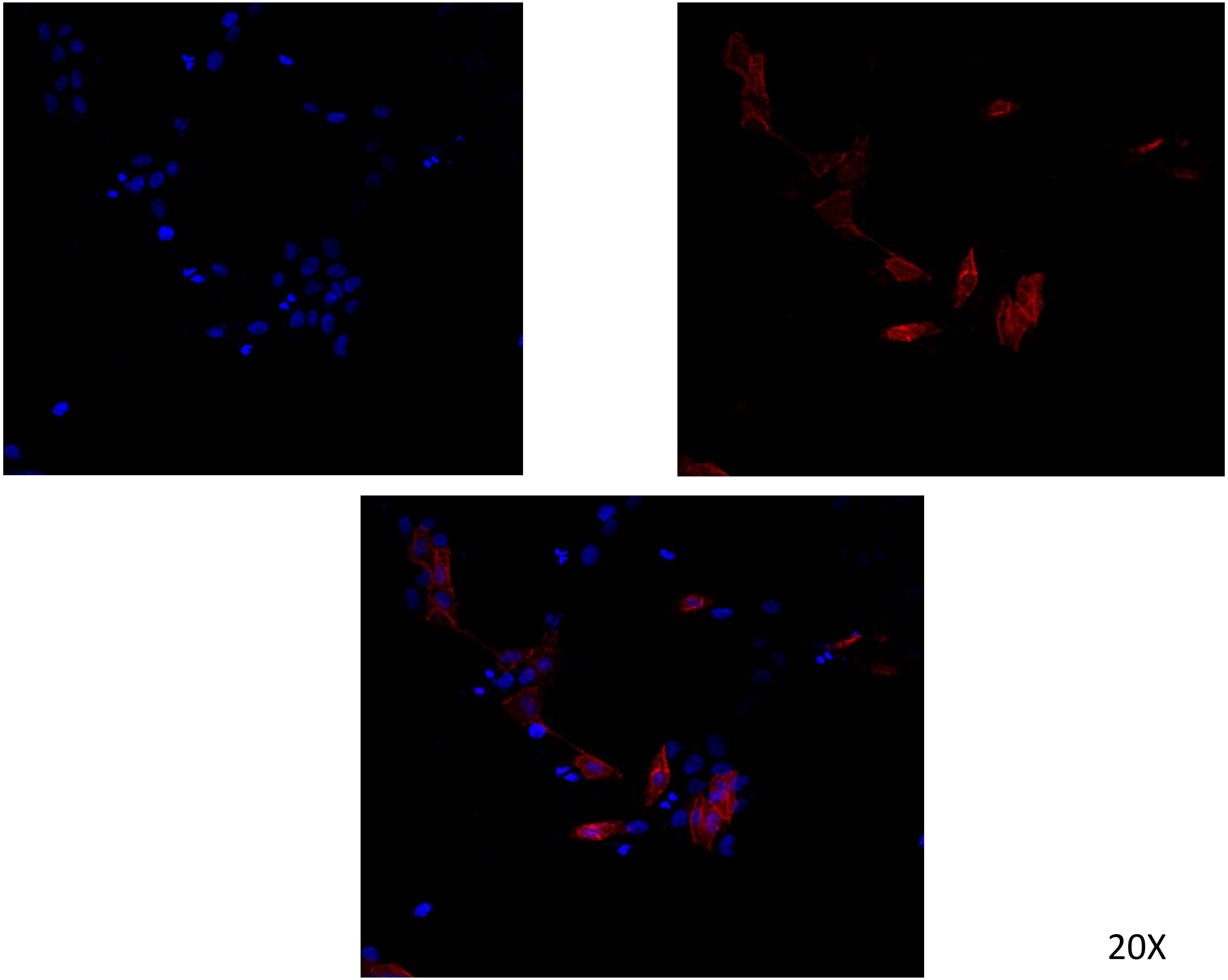

20X

Figure S18. Figure 5C – expt 1 – MCF10A Blobe – KCNK9 Flag-Tag +

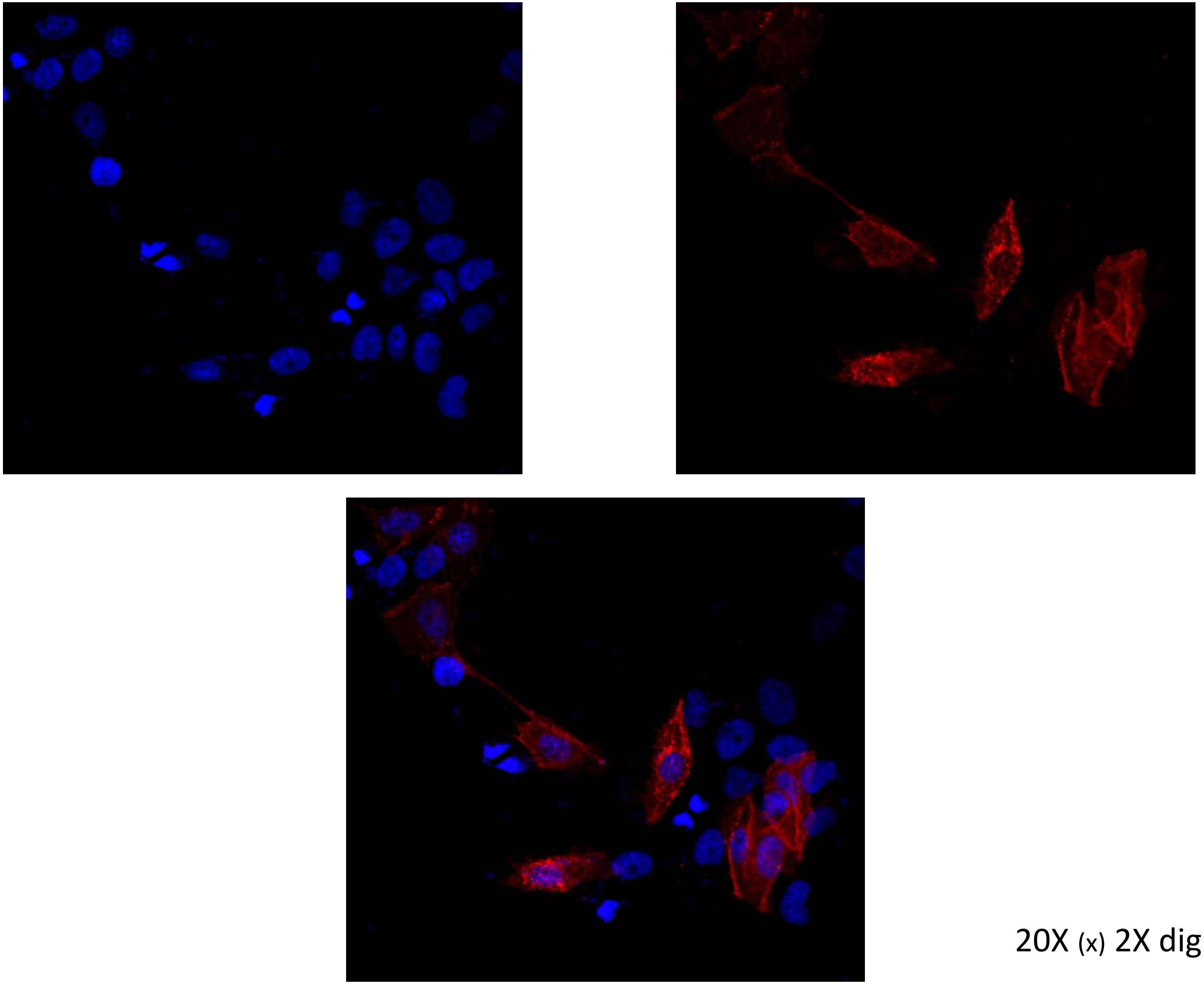

20X (x) 2X digital zoom

Figure S18. Figure 5C – expt 1 – MCF10A Blobe – KCNK9 Flag-Tag +

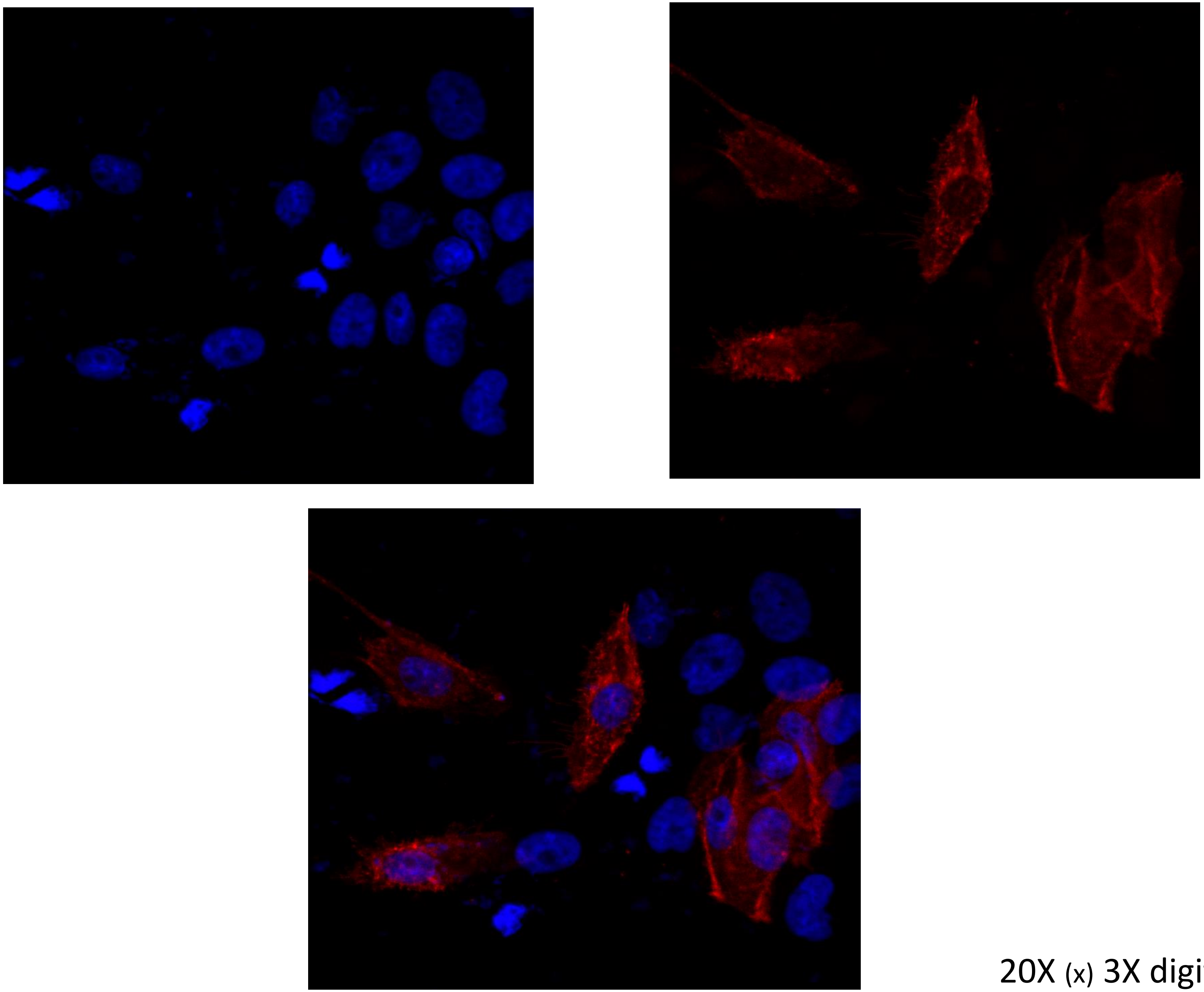

20X (x) 3X digital zoom

Figure S19. Figure 5C – expt 1 – MCF10A Blobe – Empty Flag-Tag +

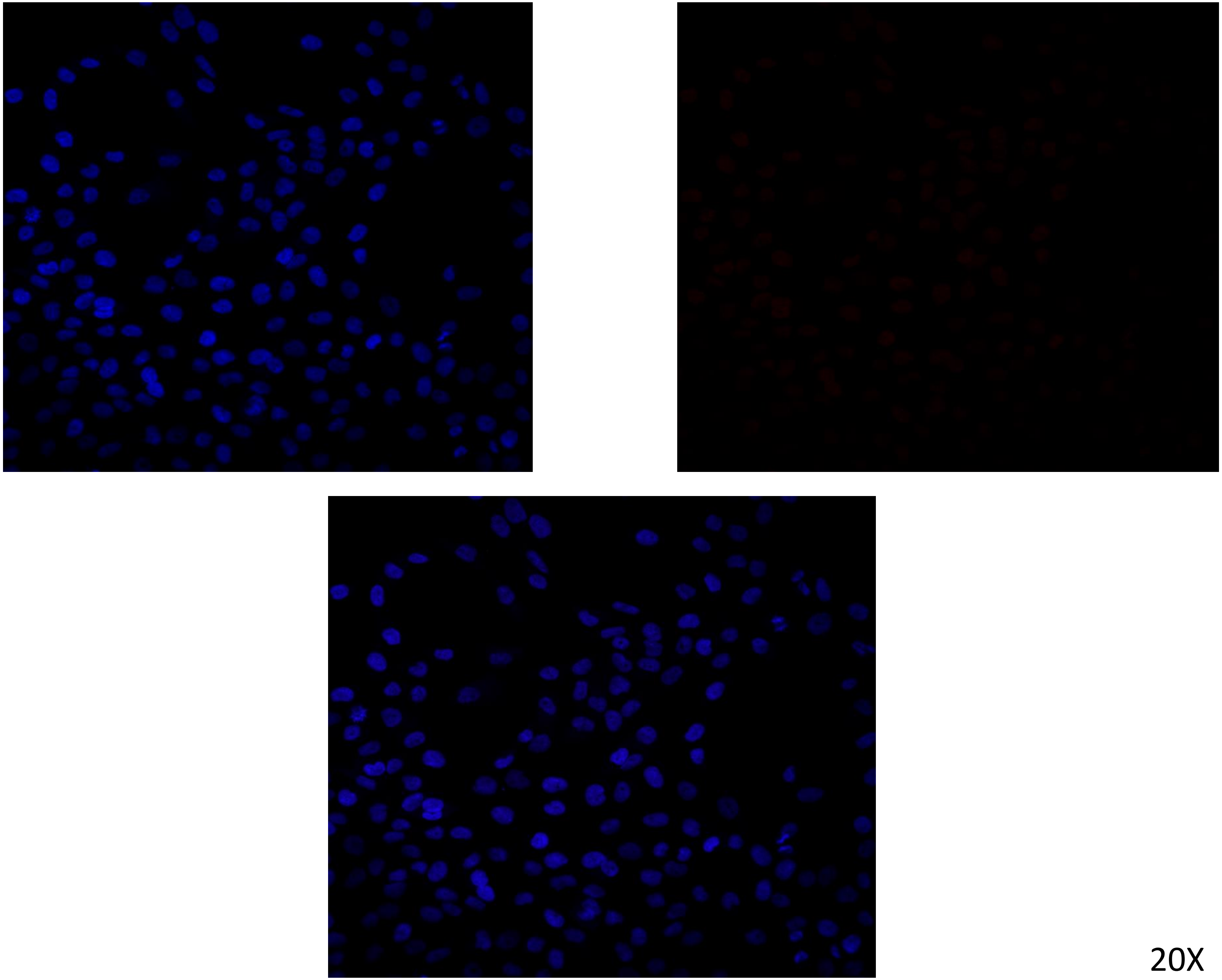

20X

Figure S19. Figure 5C – expt 1 – MCF10A Blobe – Empty Flag-Tag +

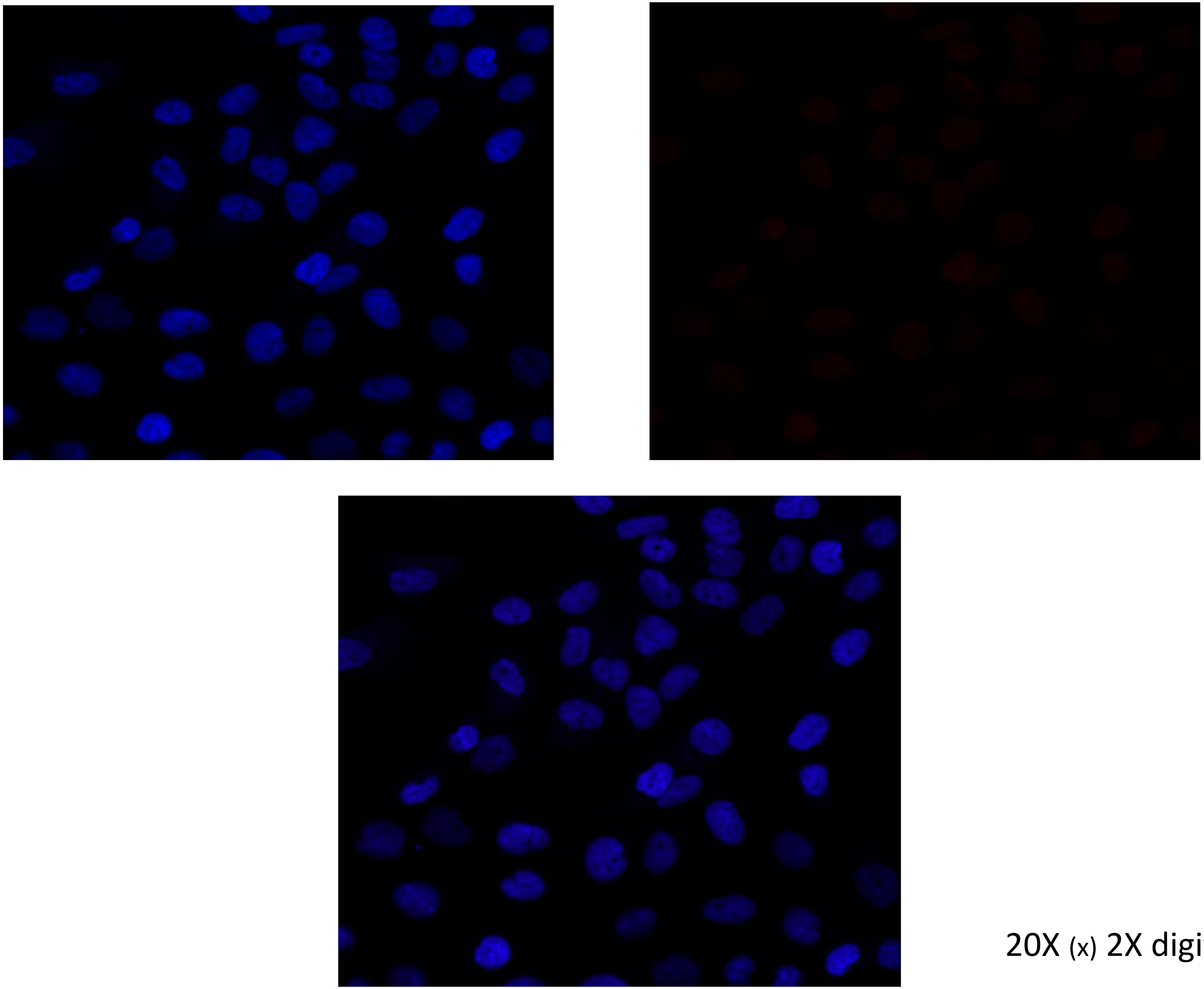

20X (x) 2X digital zoom

Figure S19. Figure 5C – expt 1 – MCF10A Blobe – Empty Flag-Tag +

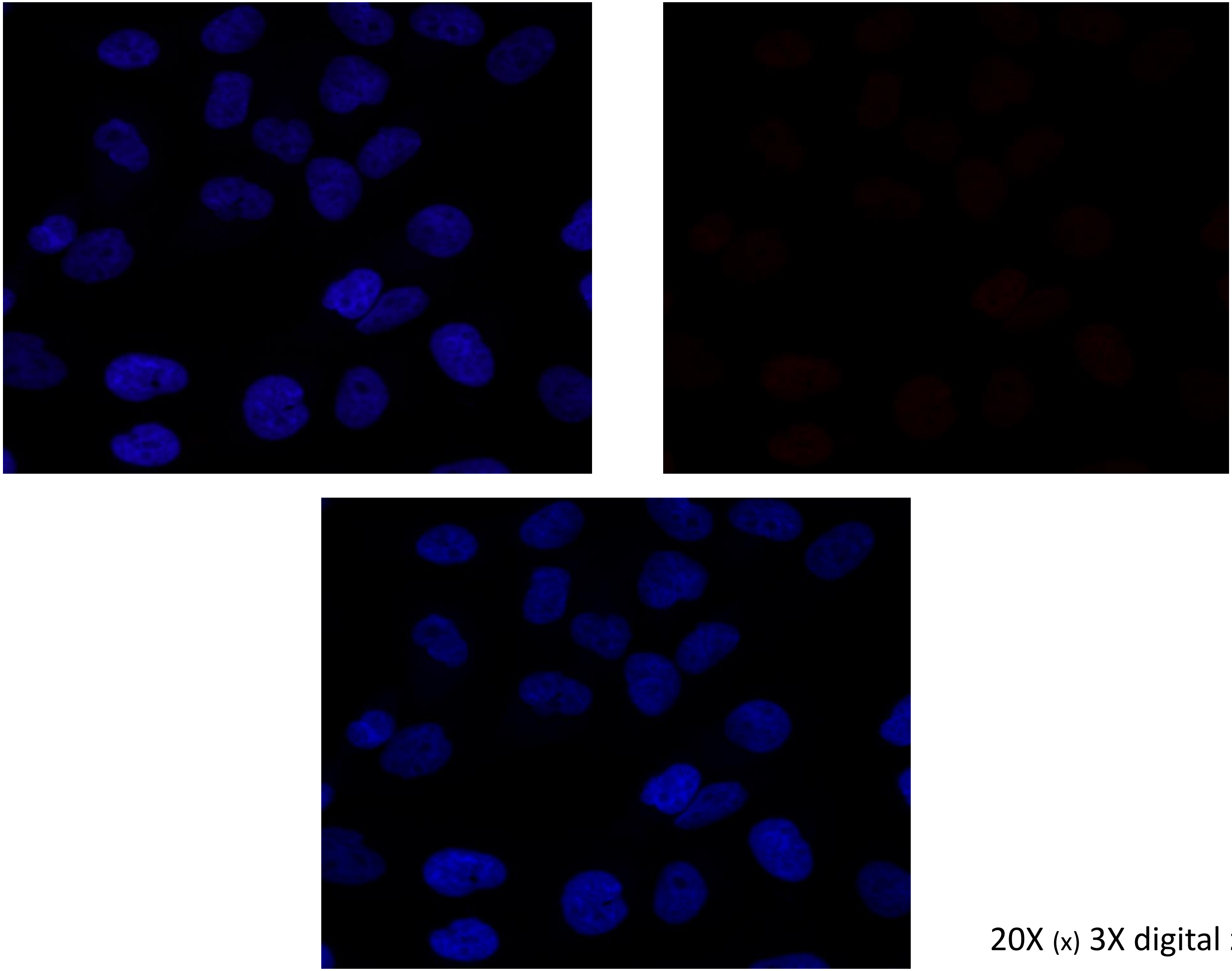

20X (x) 3X digital zoom

Figure S20. Figure 5C – expt 1 – MCF10A Blobe – Transfection Reagent alone +

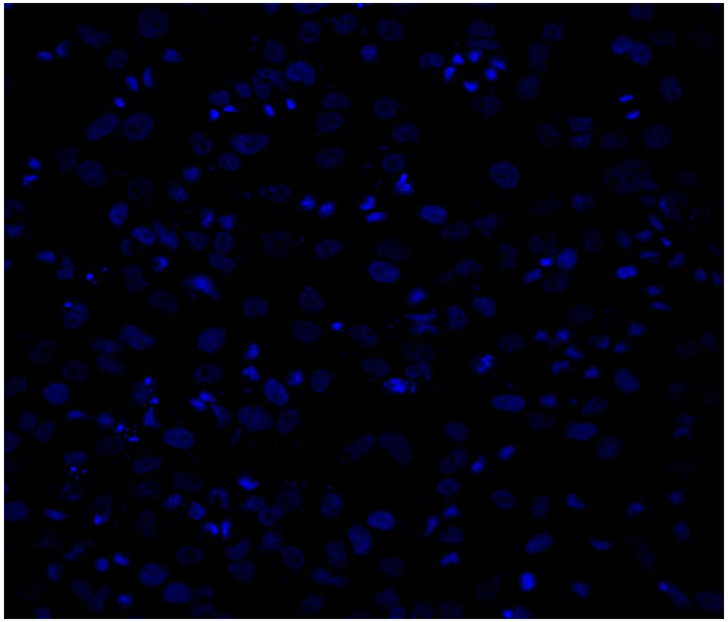

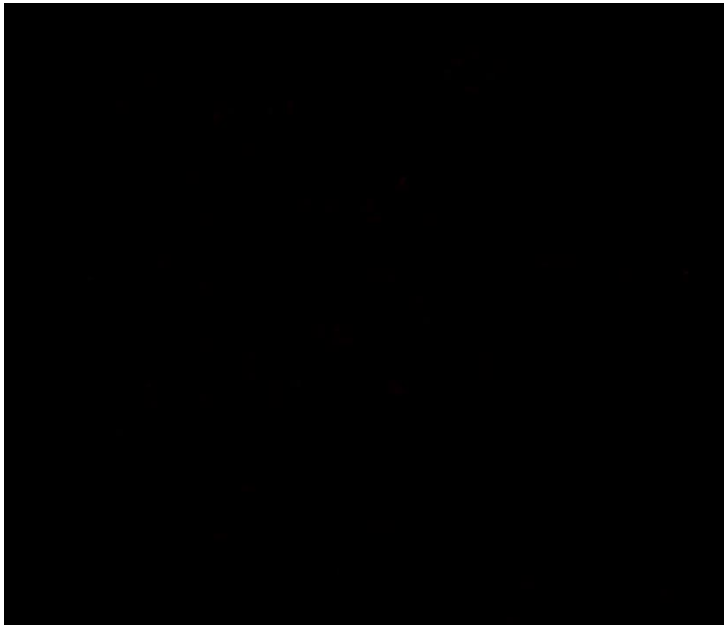

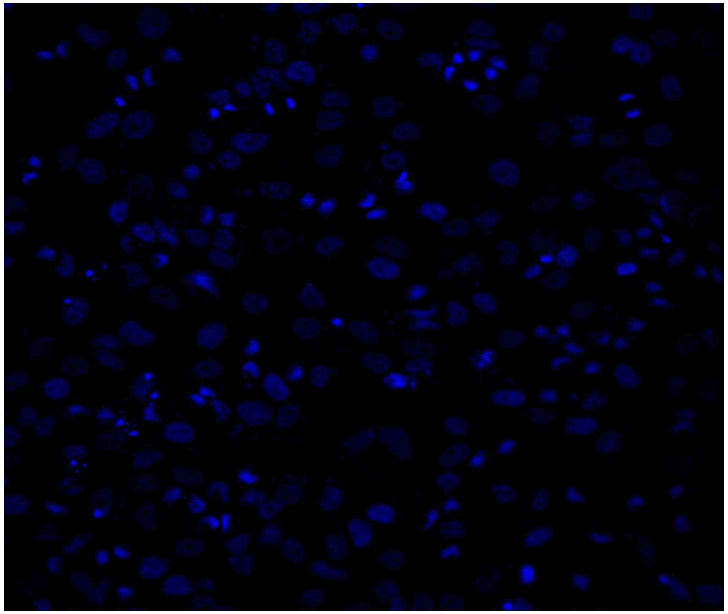

20X

Figure S20. Figure 5C – expt 1 – MCF10A Blobe – Transfection Reagent alone +

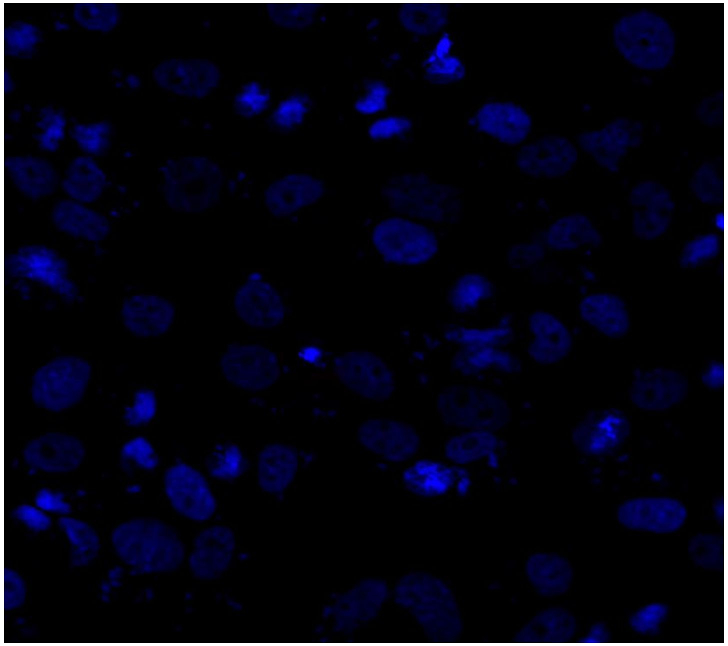

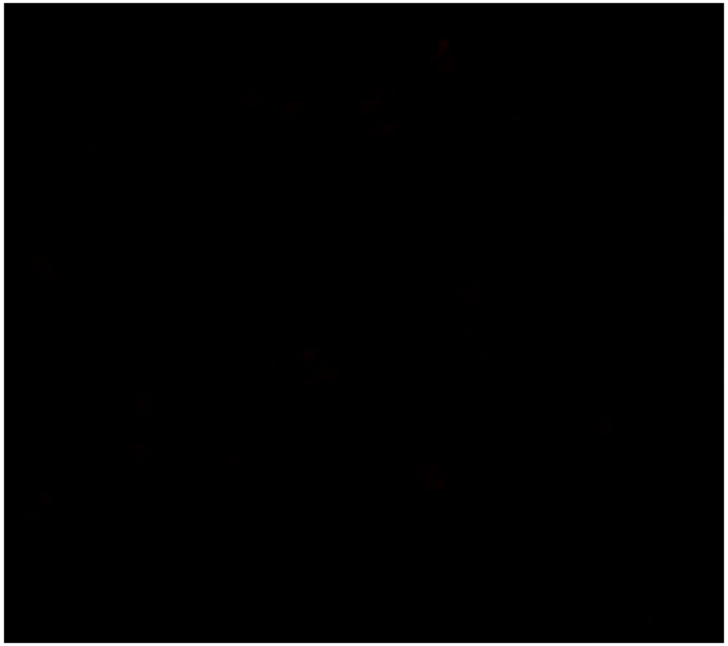

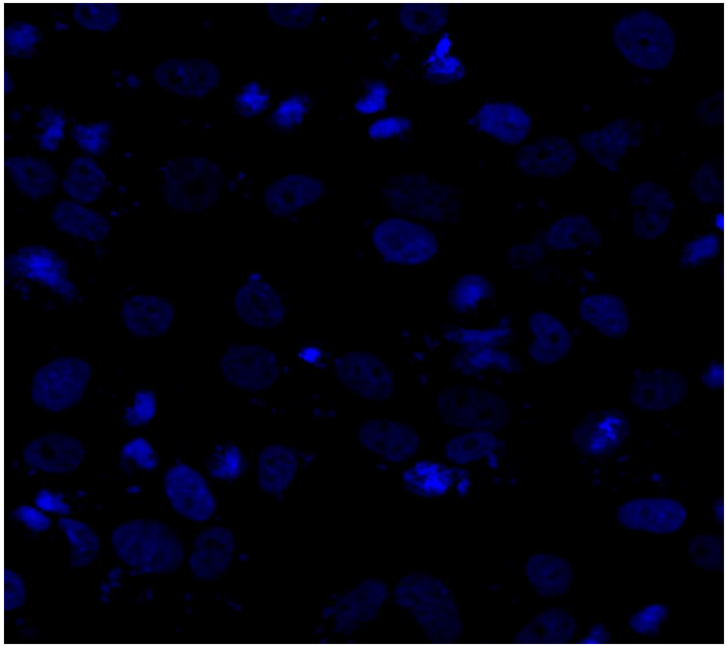

20X (x) 2X digital zoom

Figure S20. Figure 5C – expt 1 – MCF10A Blobe – Transfection Reagent alone +

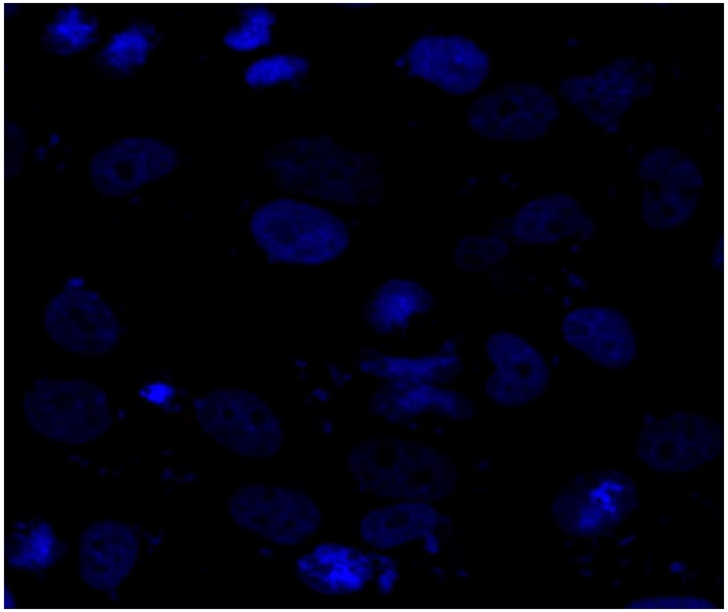

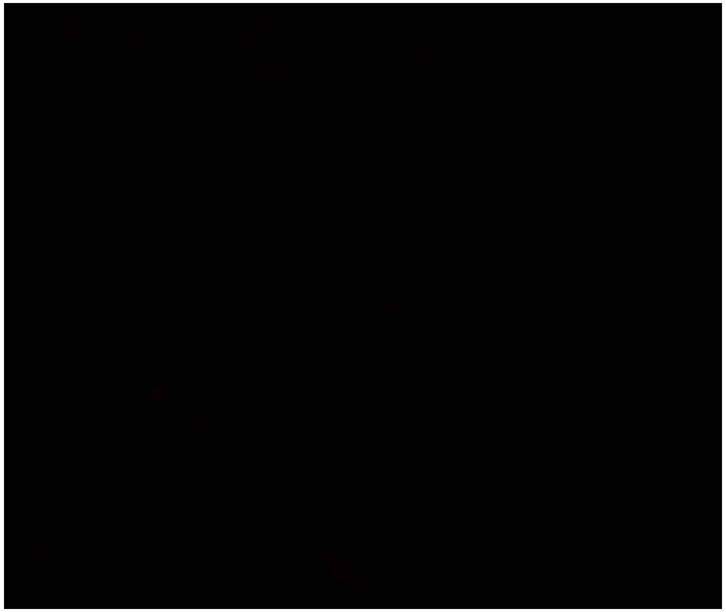

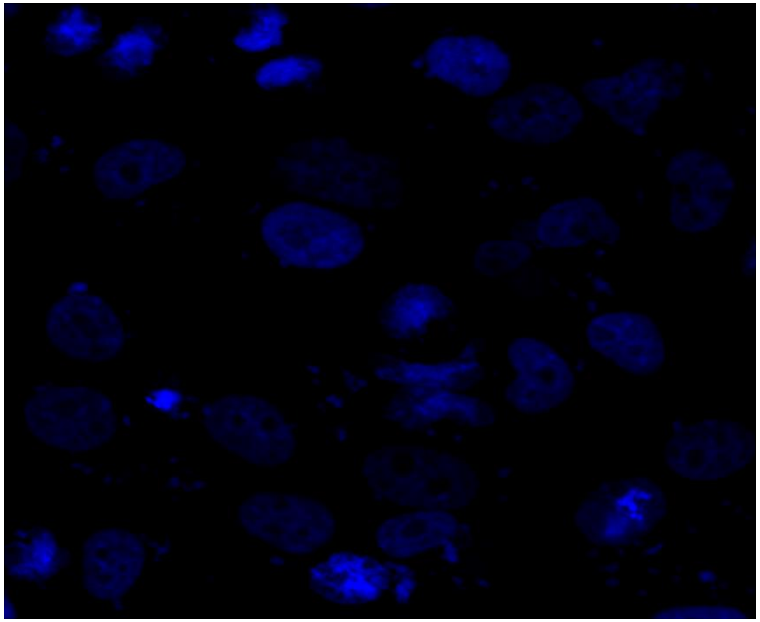

20X (x) 3X digital zoom

Figure S21. Figure 5C – expt 1 – MCF10A Blobe – Control/No Transfection +

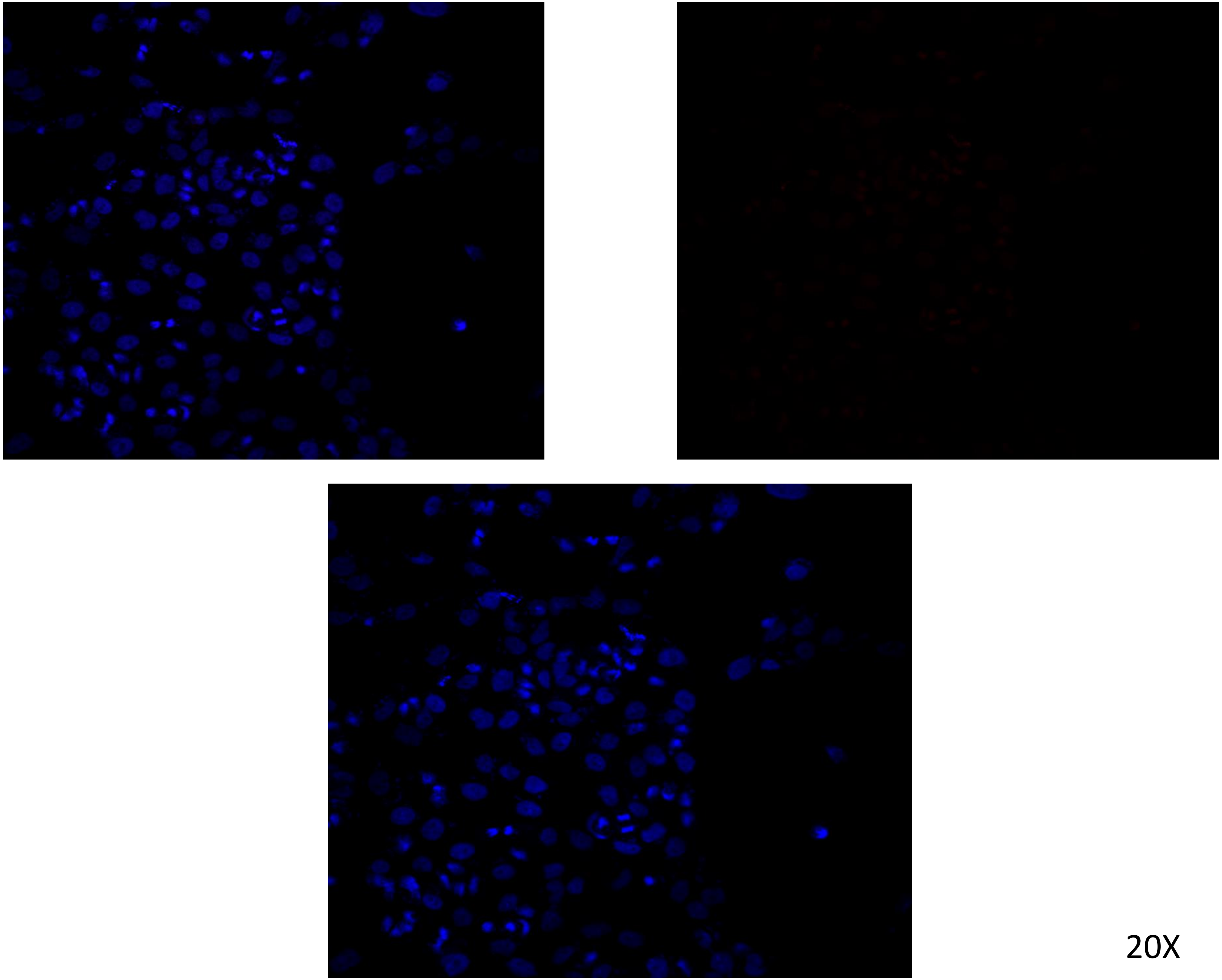

20X

Figure S21. Figure 5C – expt 1 – MCF10A Blobe – Control/No Transfection +

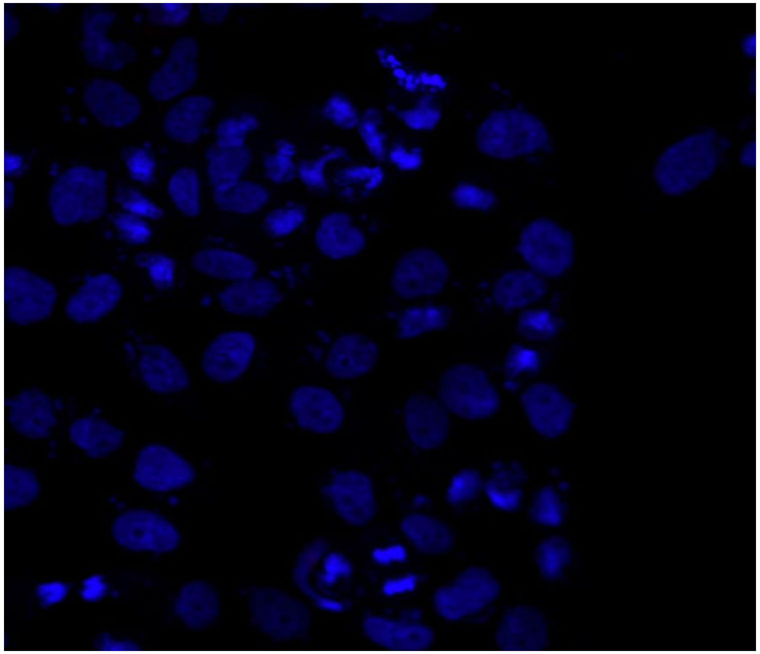

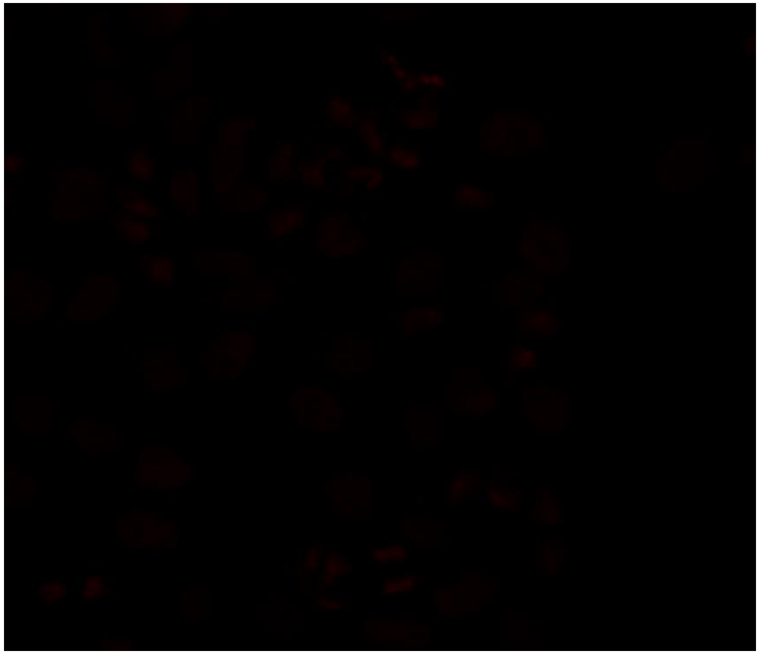

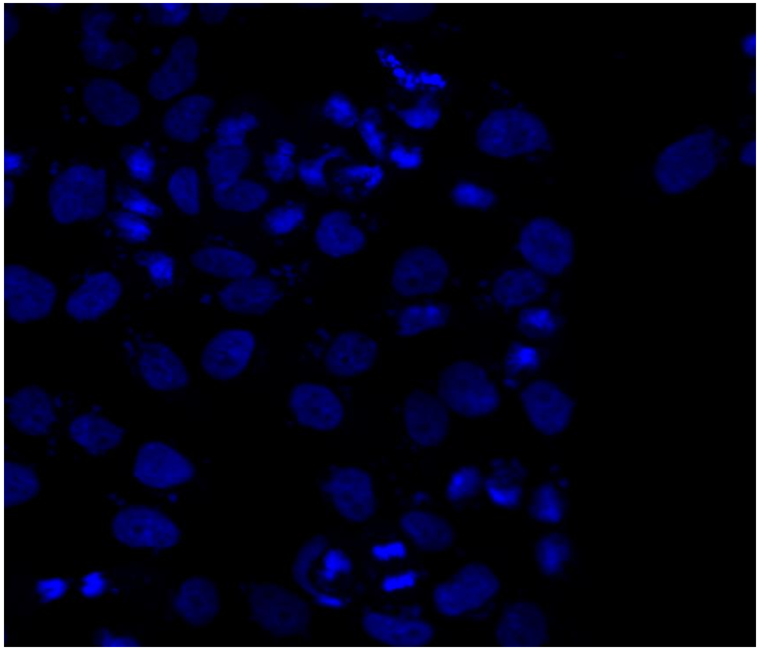

20X (x) 2X digital zoom

Figure S21. Figure 5C – expt 1 – MCF10A Blobe – Control/No Transfection +

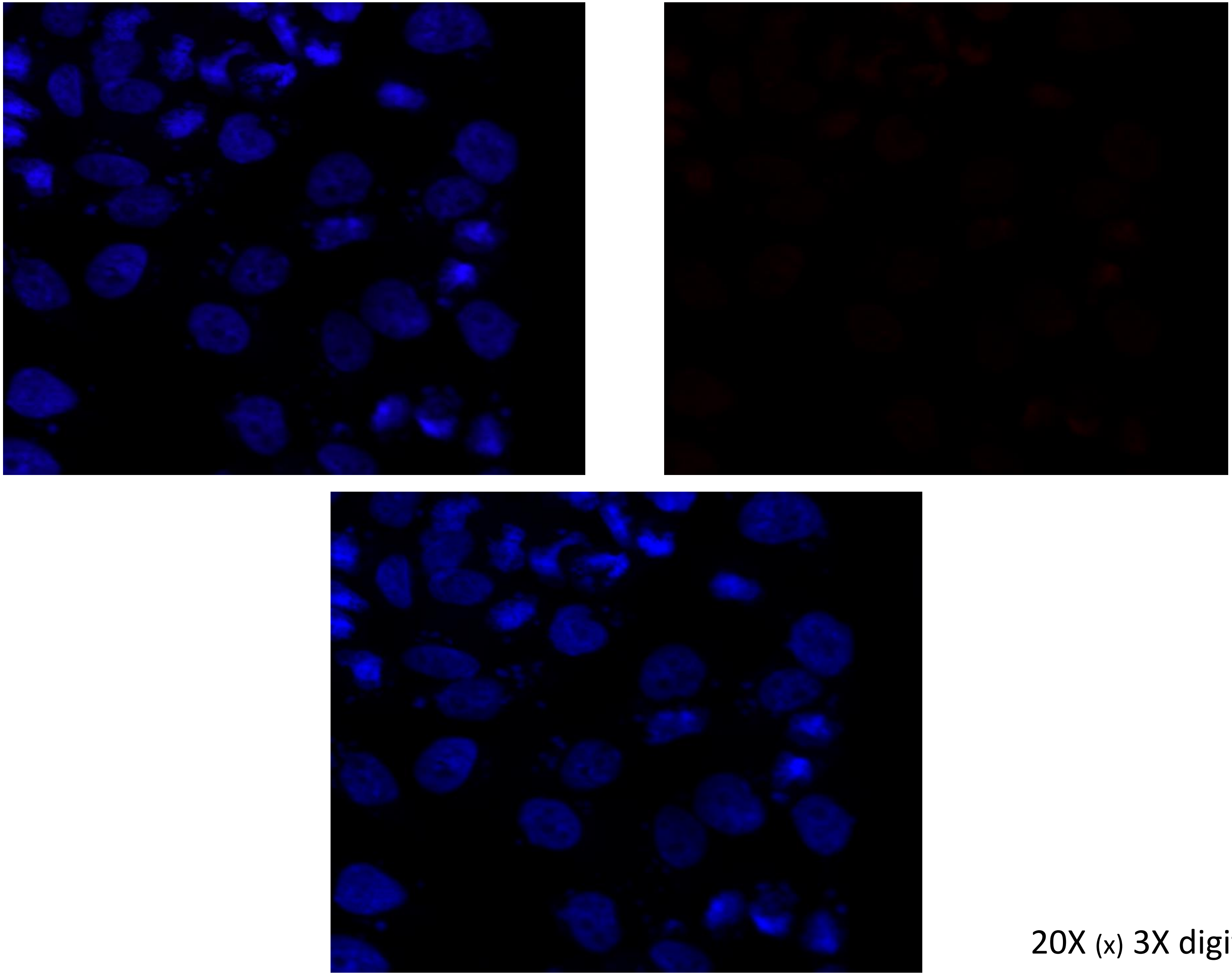

20X (x) 3X digital zoom

Figure S22. Current Figure 5B and former Figure 6A – VDAC

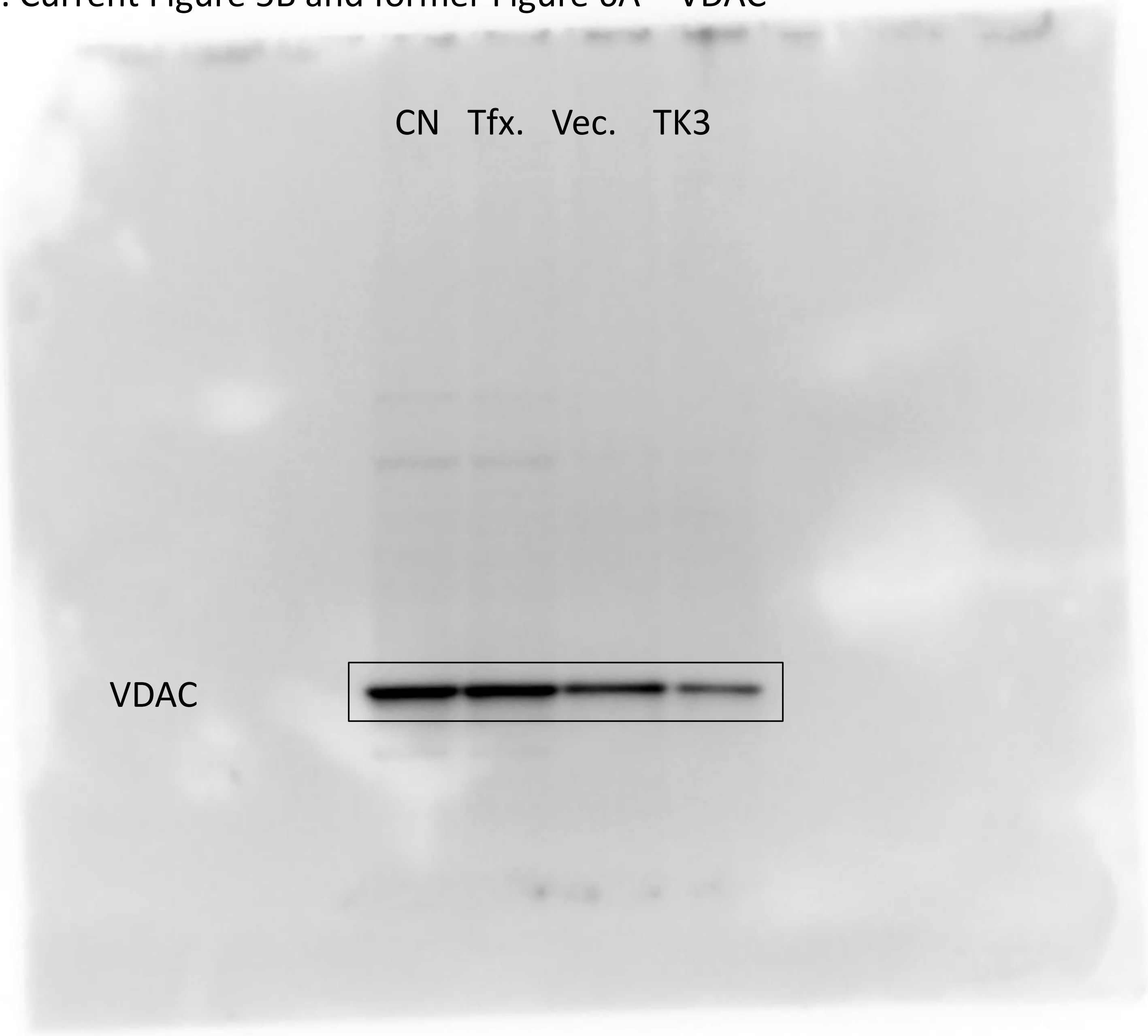

Figure S23. Current Figure 6A and Prior Figure 5D – HEK293 – TASK3 – 42KDa

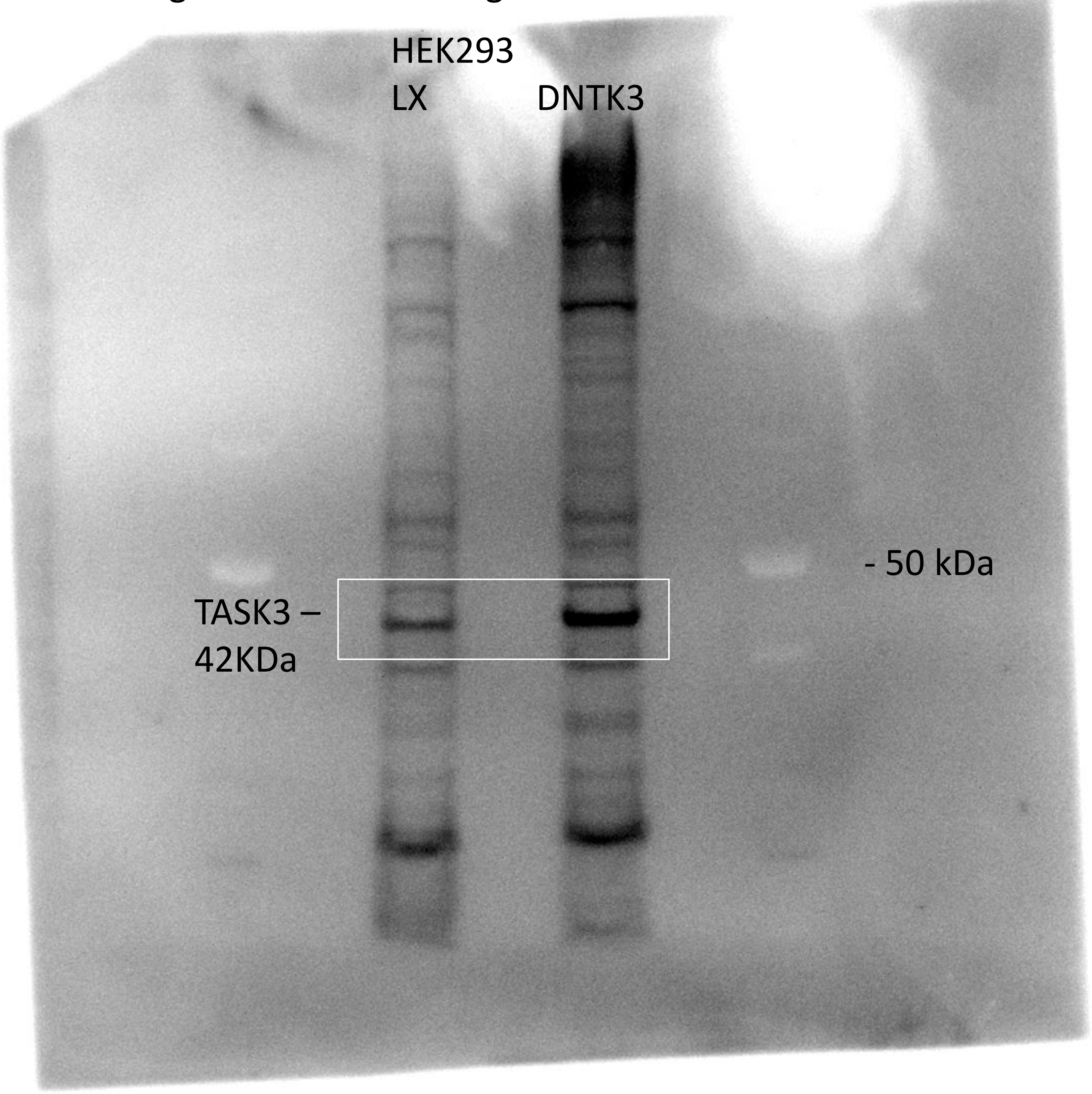

Figure S24. Current Figure 6A and Prior Figure 5D – HEK293 –GAPDH

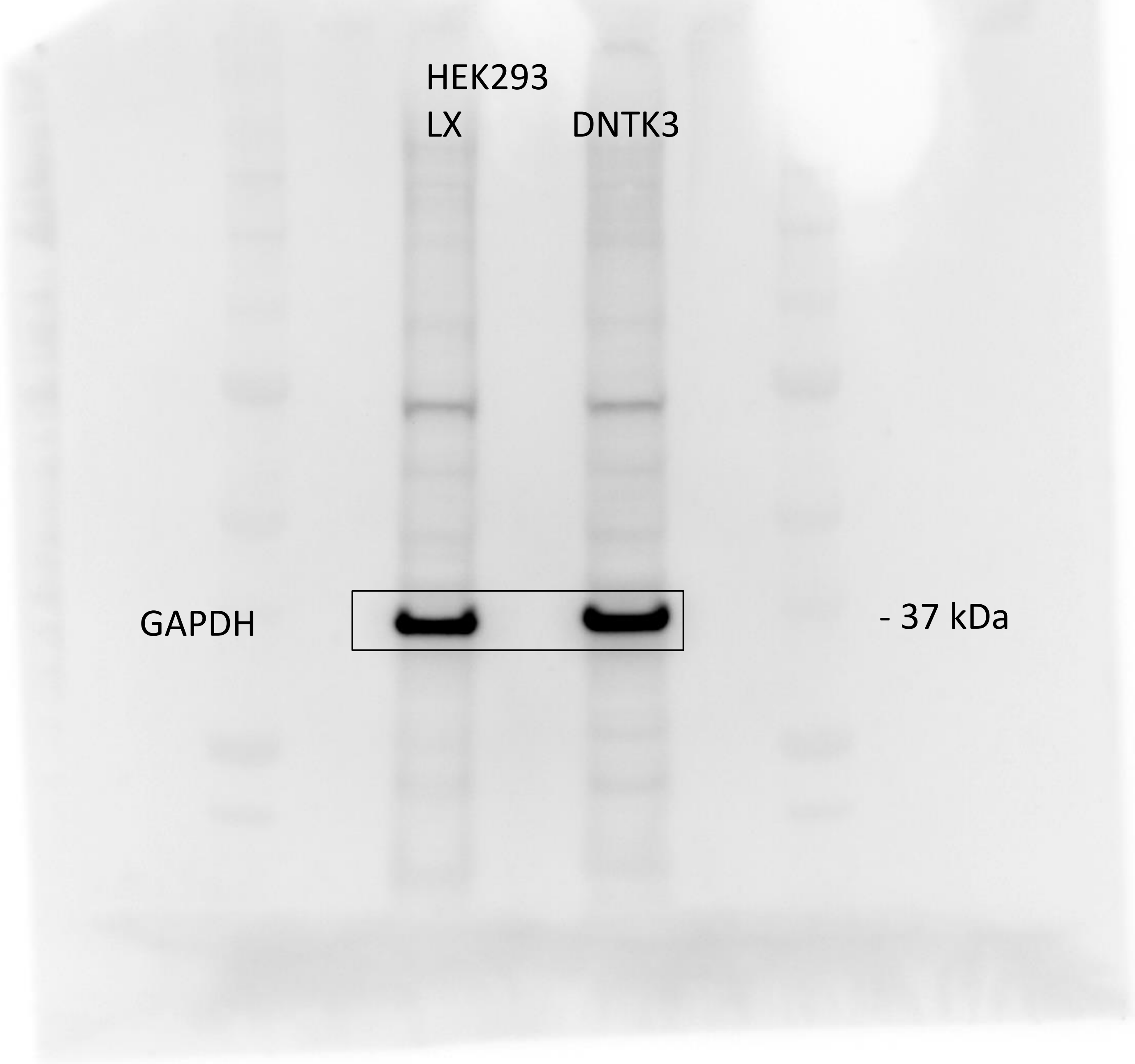

Figure S25. Current Figure 6A and Prior Figure 5D – MDA231 – TASK3 – 42KDa

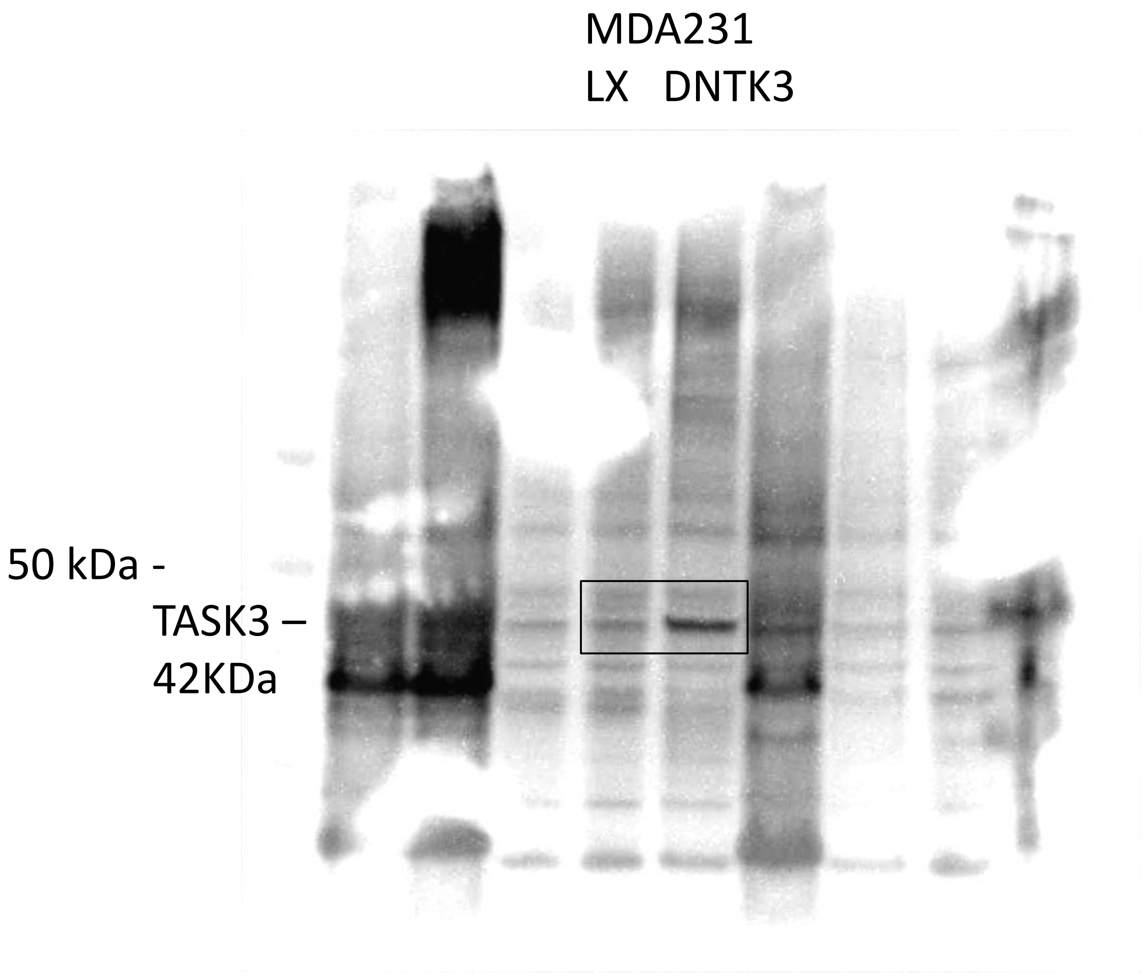

Figure S26. Current Figure 6A and Prior Figure 5D – MDA231 – GAPDH

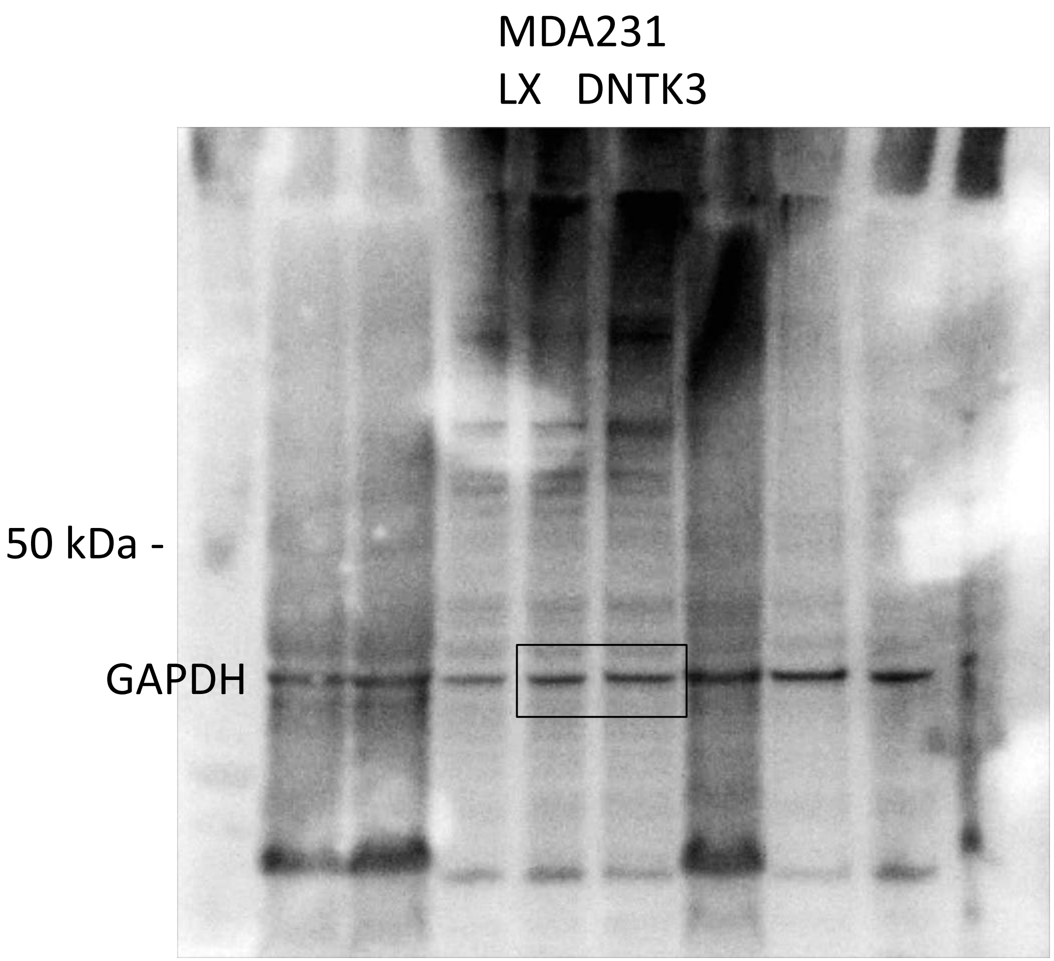

Figure S27. Current Figure 6A and Prior Figure 5D – DKAT – TASK3

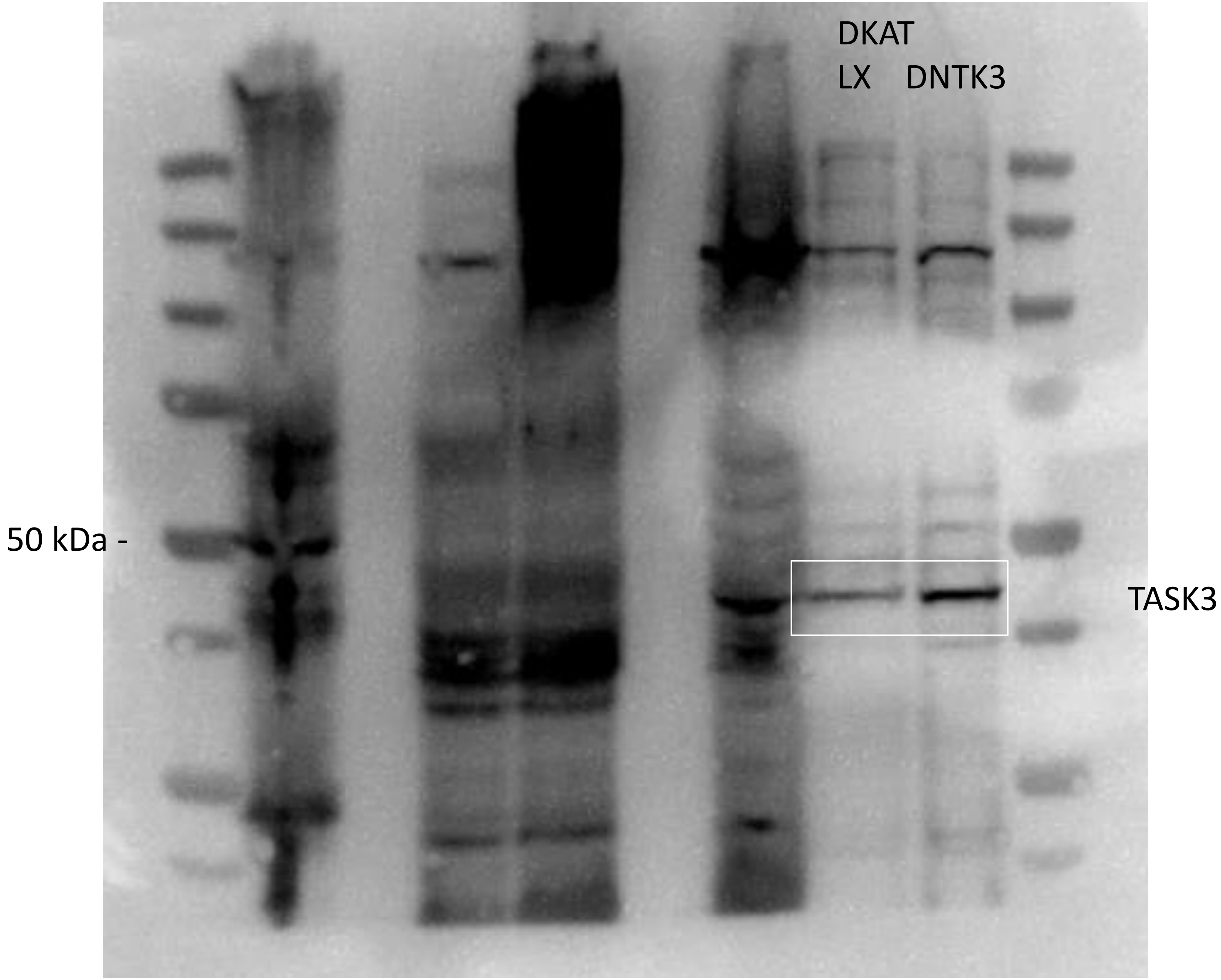

Figure S28. Current Figure 6A and Prior Figure 5D – DKAT – GAPDH

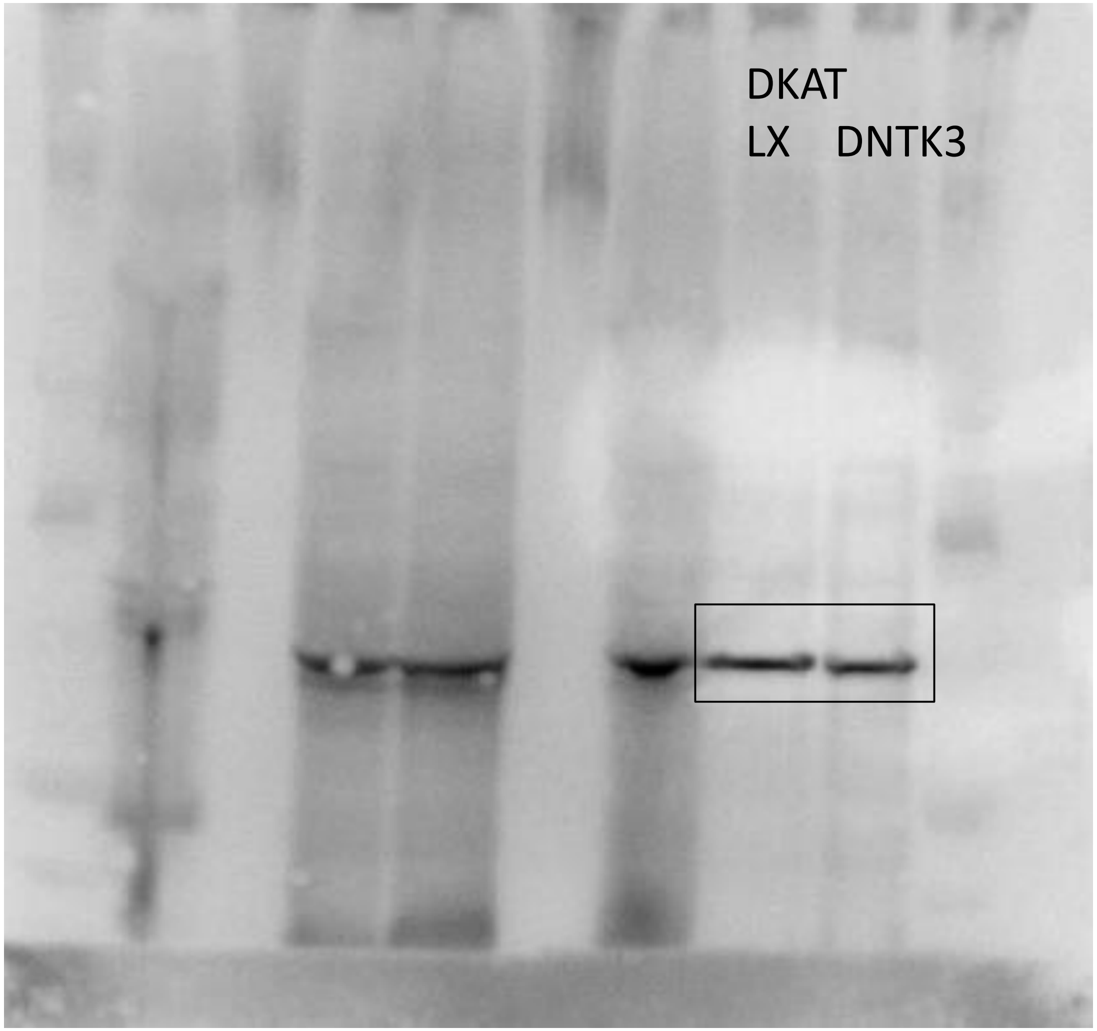

Figure S29. Current Figure 6A and Prior Figure 5D – SUM225 – TASK3 – 42KDa

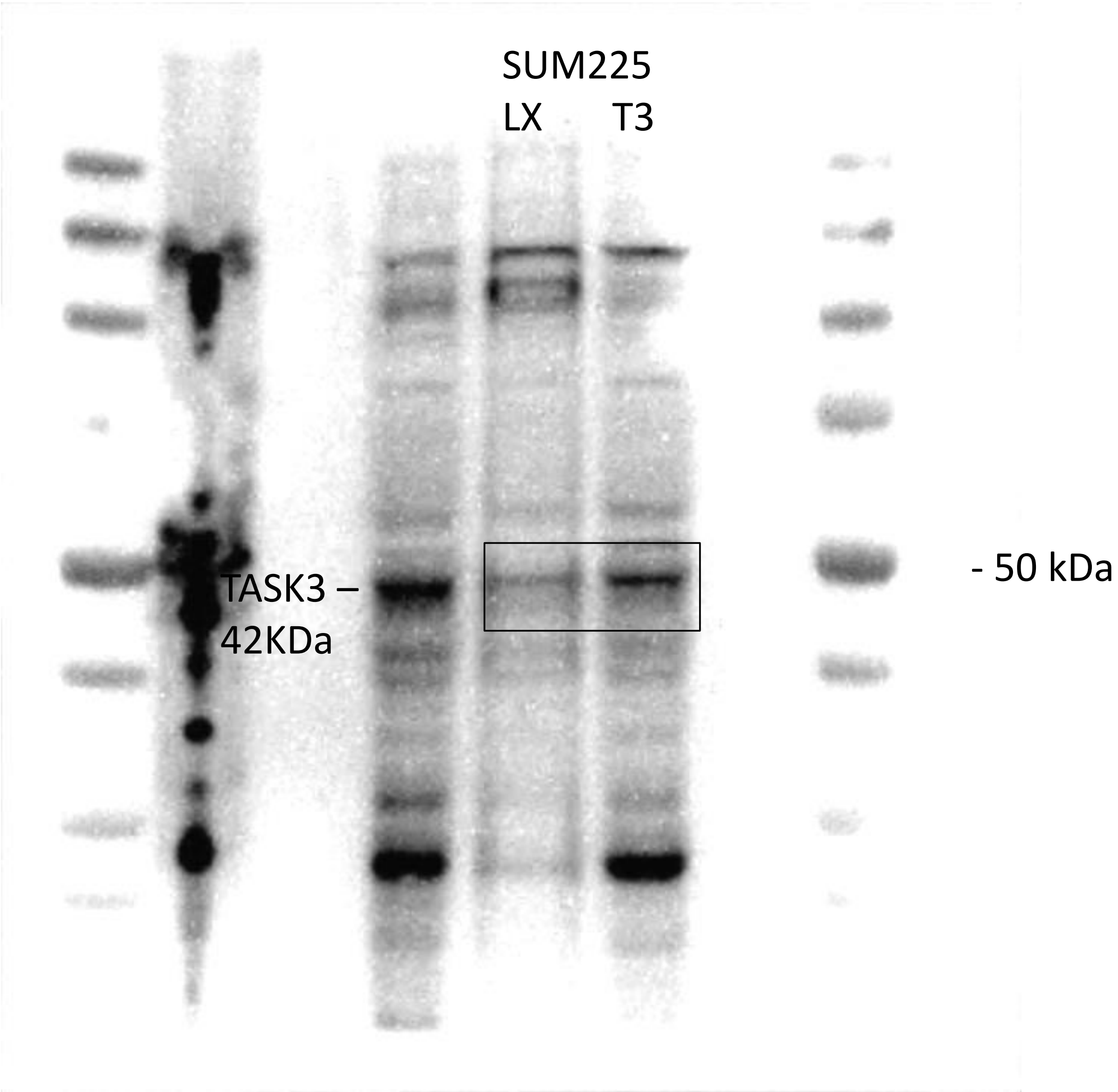

Figure S30. Current Figure 6A and Prior Figure 5D – SUM225 – GAPDH

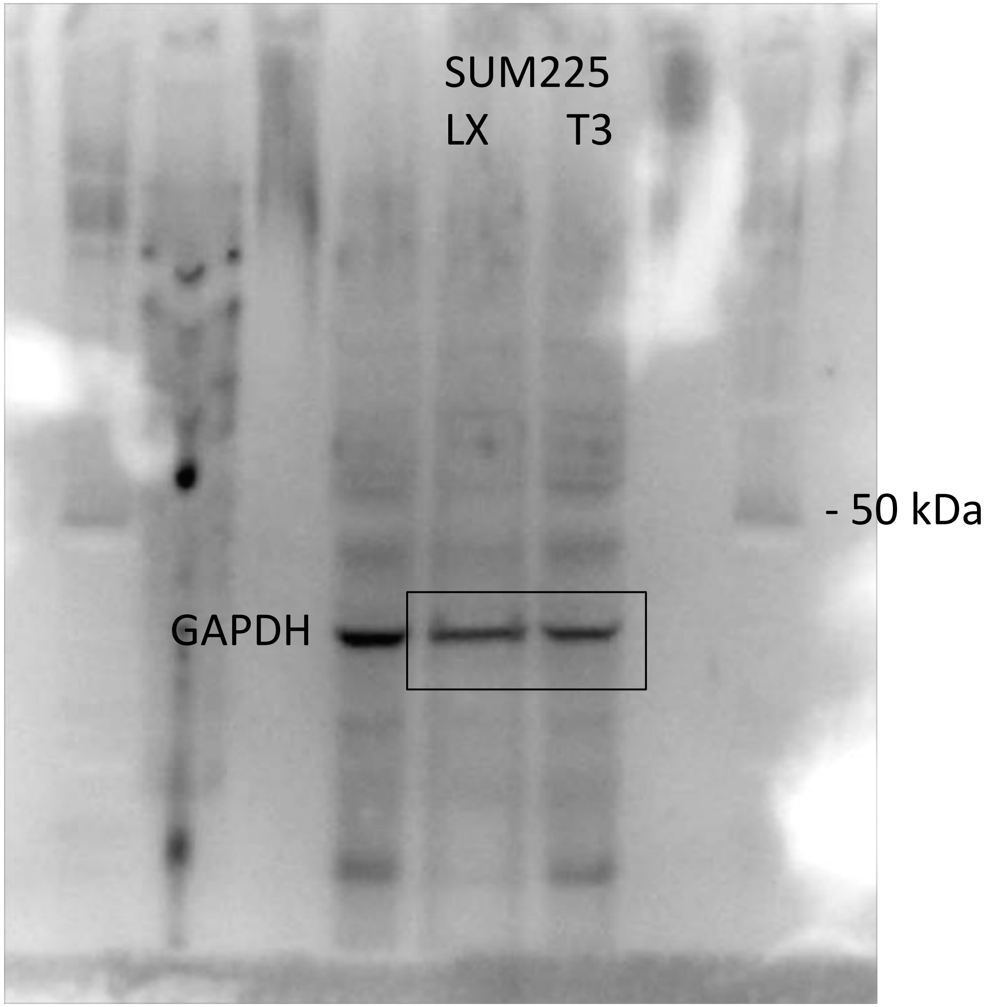

Supplement: Supplementary file 1 [file cancers-13-06031-s001.zip › cancers-1487488-supplementary.pdf]
